# Supplementary figures and images for: A new application of multiplex PCR combined with membrane biochip assay for rapid detection of 9 common pathogens in sepsis (part 1 of 2)
Source: PeerJ. 2023 May 12;11:e15325. doi: 10.7717/peerj.15325 (PMC10184654; doi:10.7717/peerj.15325)

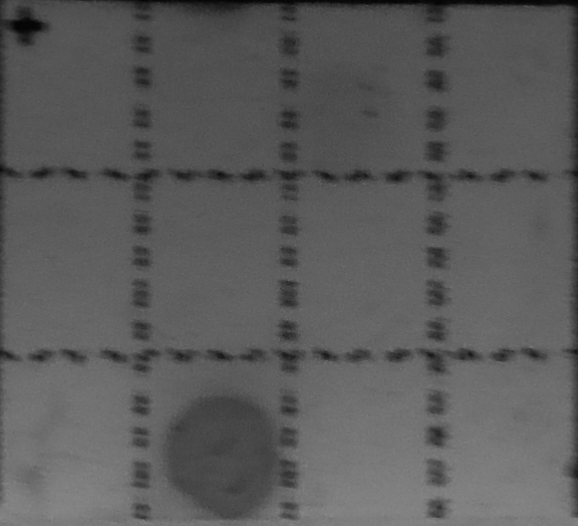

Supplement: Supplemental Information 1 [file peerj-11-15325-s001.zip › Raw Data/Results of 179 clinical samples of septicemia by membrane microarray (grayscale)-1/559752.tif]

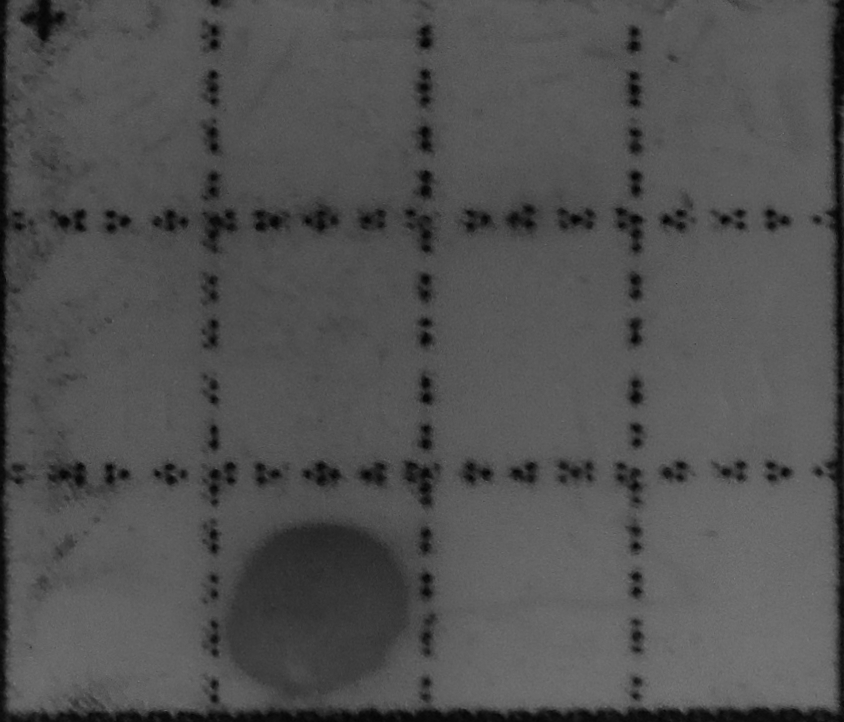

Supplement: Supplemental Information 1 [file peerj-11-15325-s001.zip › Raw Data/Results of 179 clinical samples of septicemia by membrane microarray (grayscale)-1/559792.tif]

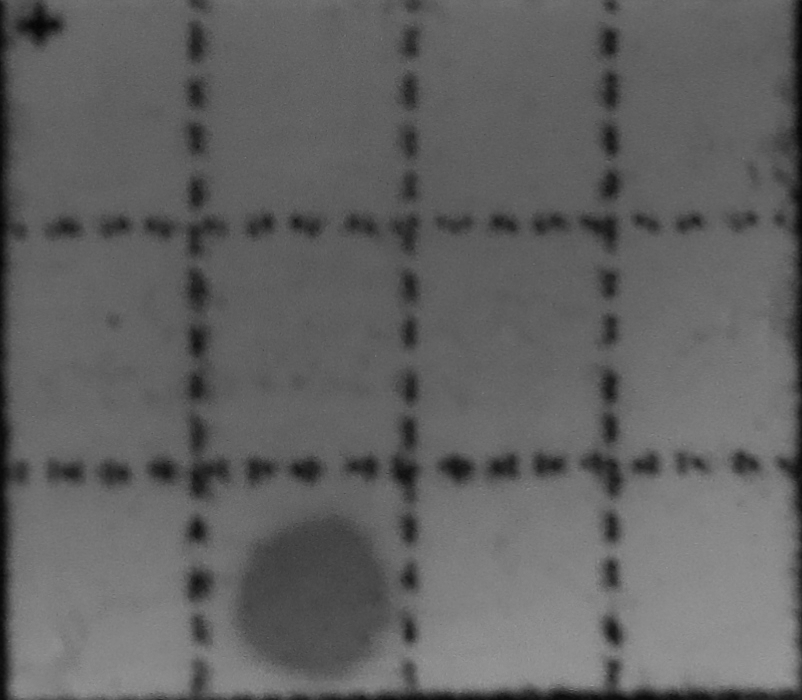

Supplement: Supplemental Information 1 [file peerj-11-15325-s001.zip › Raw Data/Results of 179 clinical samples of septicemia by membrane microarray (grayscale)-1/559927.tif]

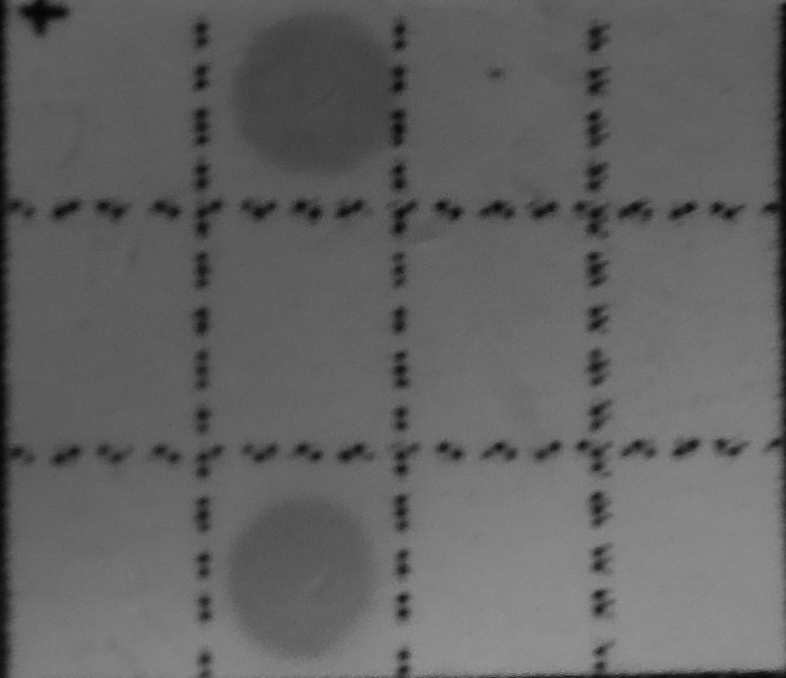

Supplement: Supplemental Information 1 [file peerj-11-15325-s001.zip › Raw Data/Results of 179 clinical samples of septicemia by membrane microarray (grayscale)-1/561683.tif]

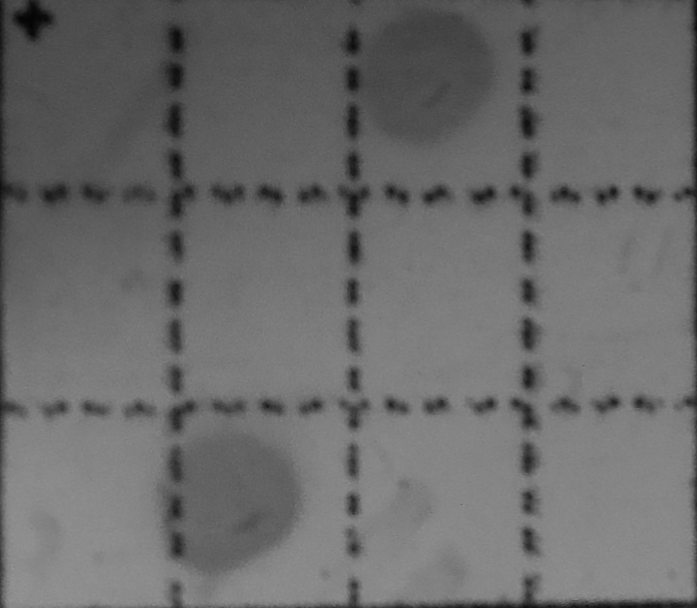

Supplement: Supplemental Information 1 [file peerj-11-15325-s001.zip › Raw Data/Results of 179 clinical samples of septicemia by membrane microarray (grayscale)-1/563119.tif]

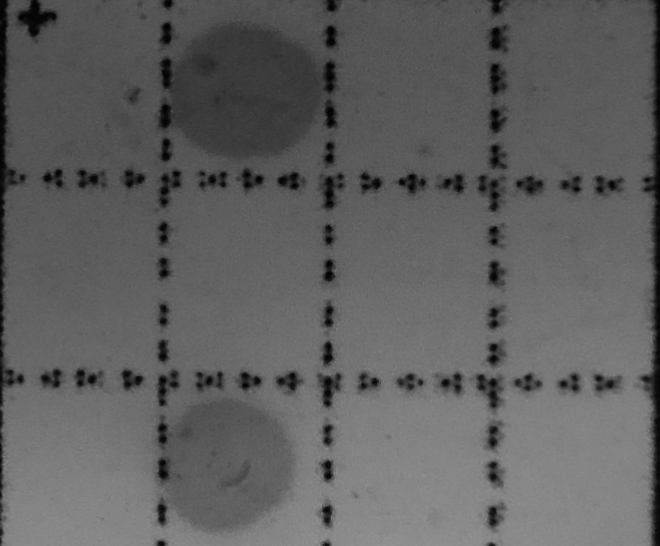

Supplement: Supplemental Information 1 [file peerj-11-15325-s001.zip › Raw Data/Results of 179 clinical samples of septicemia by membrane microarray (grayscale)-1/563625.tif]

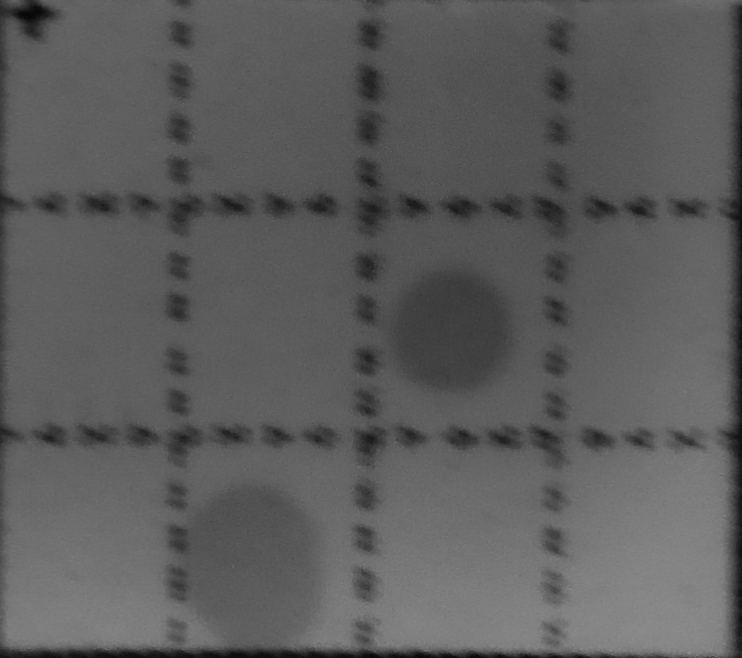

Supplement: Supplemental Information 1 [file peerj-11-15325-s001.zip › Raw Data/Results of 179 clinical samples of septicemia by membrane microarray (grayscale)-1/563701.tif]

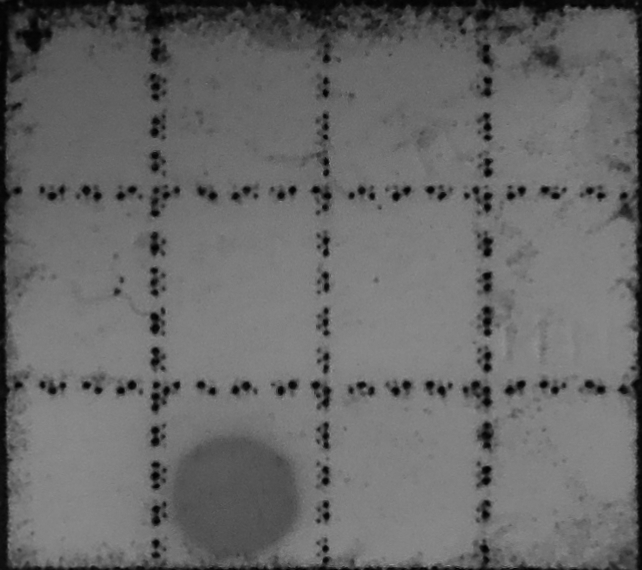

Supplement: Supplemental Information 1 [file peerj-11-15325-s001.zip › Raw Data/Results of 179 clinical samples of septicemia by membrane microarray (grayscale)-1/565287.tif]

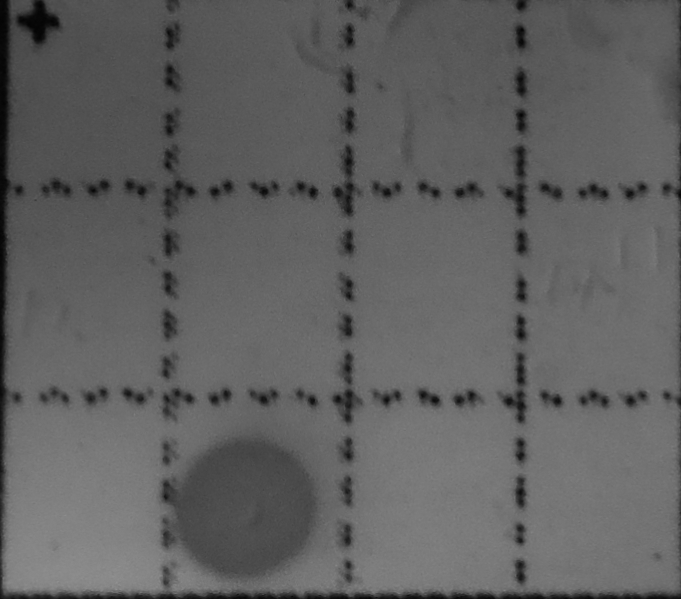

Supplement: Supplemental Information 1 [file peerj-11-15325-s001.zip › Raw Data/Results of 179 clinical samples of septicemia by membrane microarray (grayscale)-1/566716.tif]

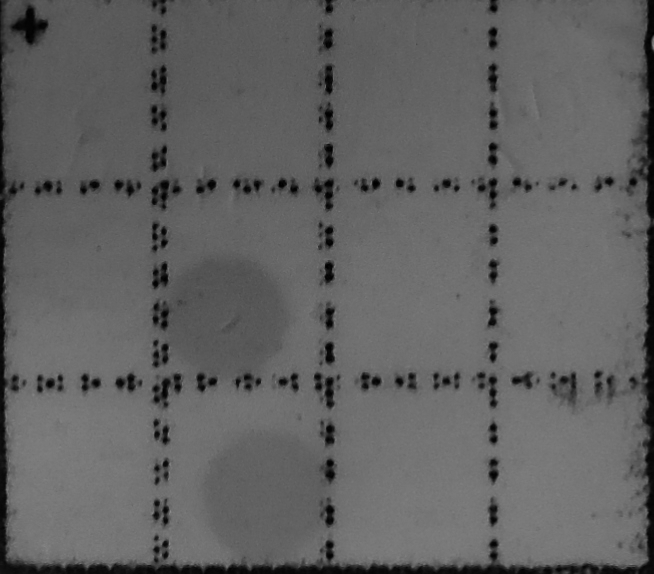

Supplement: Supplemental Information 1 [file peerj-11-15325-s001.zip › Raw Data/Results of 179 clinical samples of septicemia by membrane microarray (grayscale)-1/567226.tif]

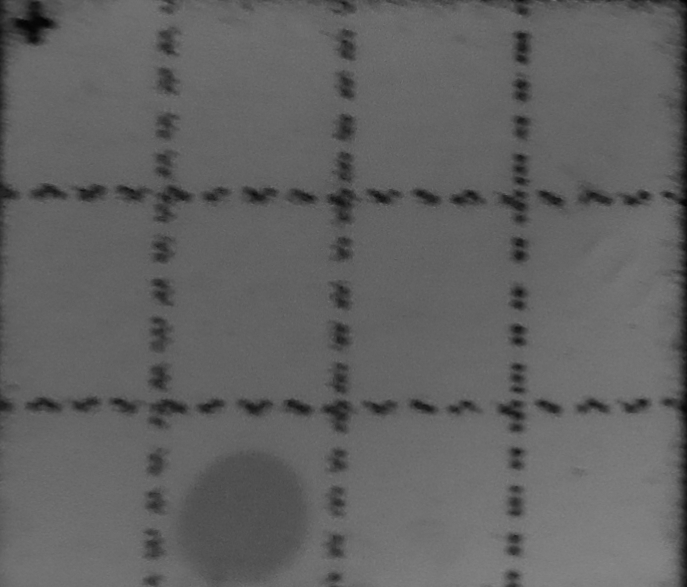

Supplement: Supplemental Information 1 [file peerj-11-15325-s001.zip › Raw Data/Results of 179 clinical samples of septicemia by membrane microarray (grayscale)-1/569216.tif]

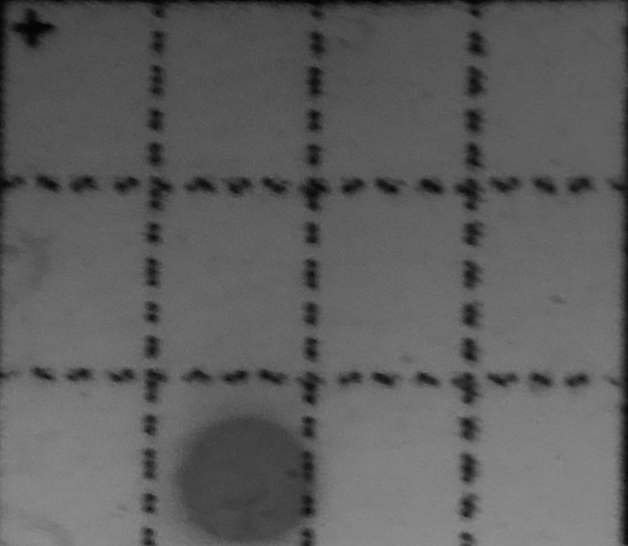

Supplement: Supplemental Information 1 [file peerj-11-15325-s001.zip › Raw Data/Results of 179 clinical samples of septicemia by membrane microarray (grayscale)-1/569510.tif]

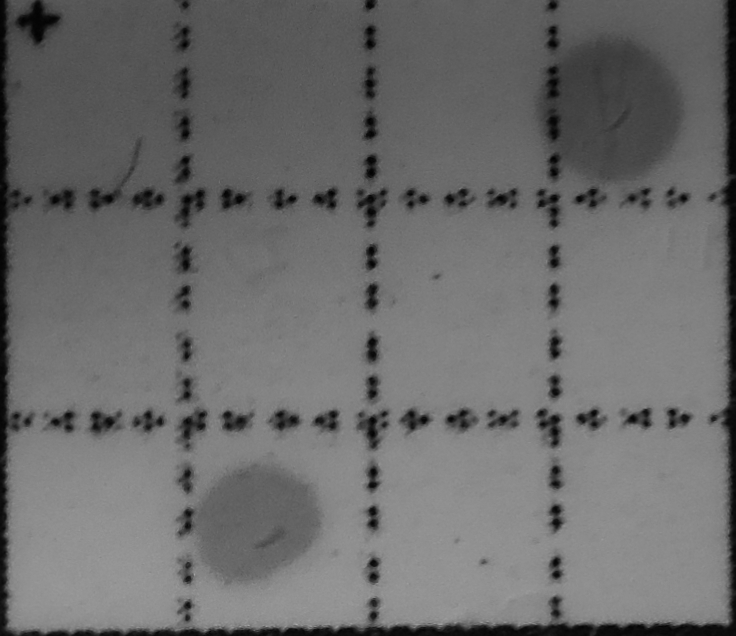

Supplement: Supplemental Information 1 [file peerj-11-15325-s001.zip › Raw Data/Results of 179 clinical samples of septicemia by membrane microarray (grayscale)-1/569935.tif]

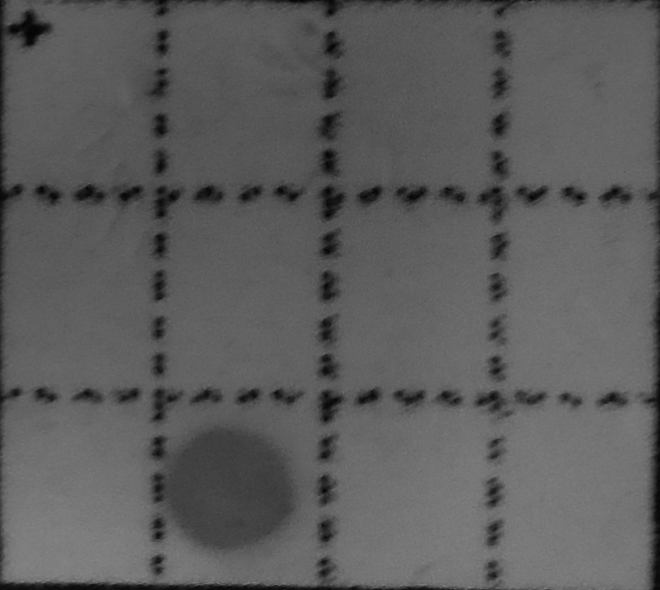

Supplement: Supplemental Information 1 [file peerj-11-15325-s001.zip › Raw Data/Results of 179 clinical samples of septicemia by membrane microarray (grayscale)-1/570575.tif]

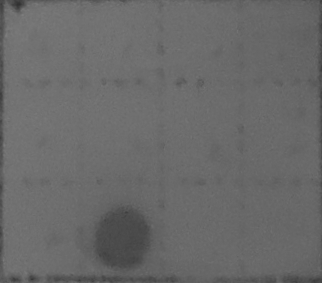

Supplement: Supplemental Information 1 [file peerj-11-15325-s001.zip › Raw Data/Results of 179 clinical samples of septicemia by membrane microarray (grayscale)-1/570892.tif]

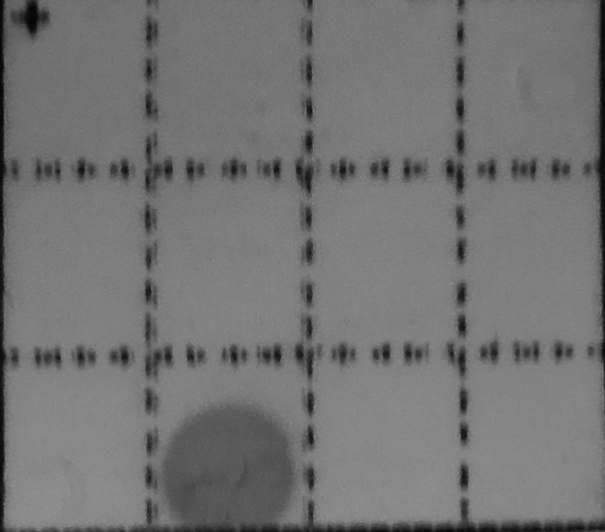

Supplement: Supplemental Information 1 [file peerj-11-15325-s001.zip › Raw Data/Results of 179 clinical samples of septicemia by membrane microarray (grayscale)-1/570900.tif]

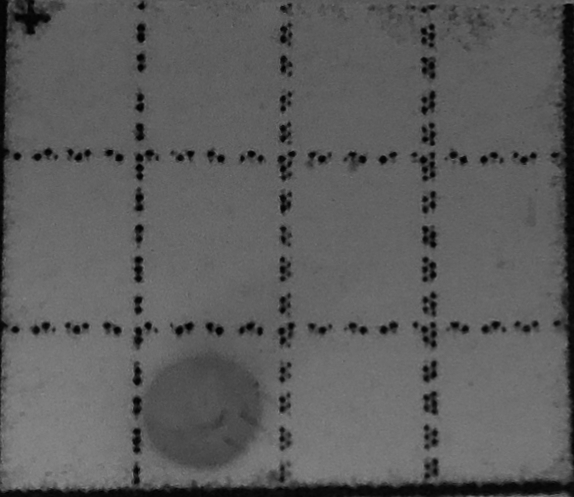

Supplement: Supplemental Information 1 [file peerj-11-15325-s001.zip › Raw Data/Results of 179 clinical samples of septicemia by membrane microarray (grayscale)-1/570902.tif]

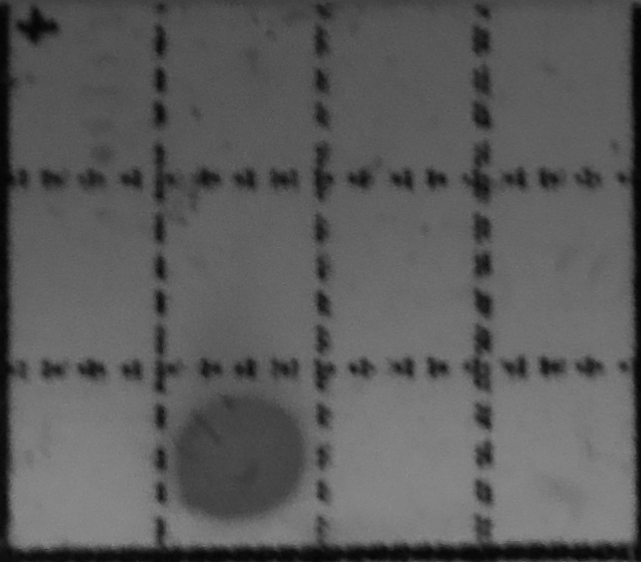

Supplement: Supplemental Information 1 [file peerj-11-15325-s001.zip › Raw Data/Results of 179 clinical samples of septicemia by membrane microarray (grayscale)-1/571245.tif]

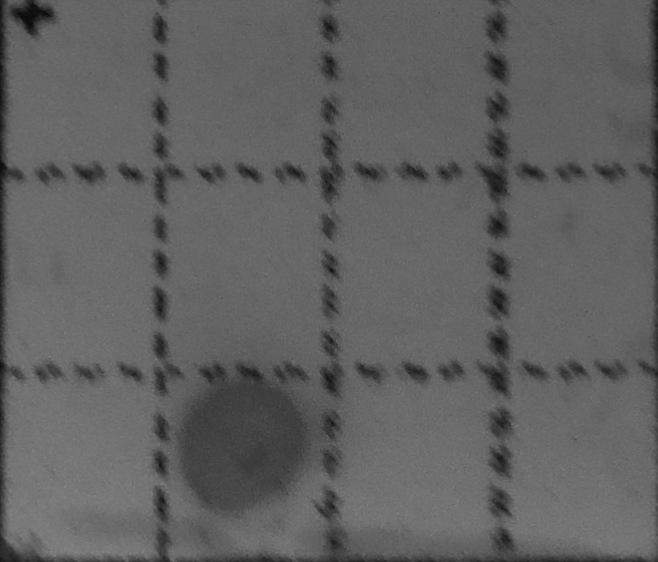

Supplement: Supplemental Information 1 [file peerj-11-15325-s001.zip › Raw Data/Results of 179 clinical samples of septicemia by membrane microarray (grayscale)-1/571730.tif]

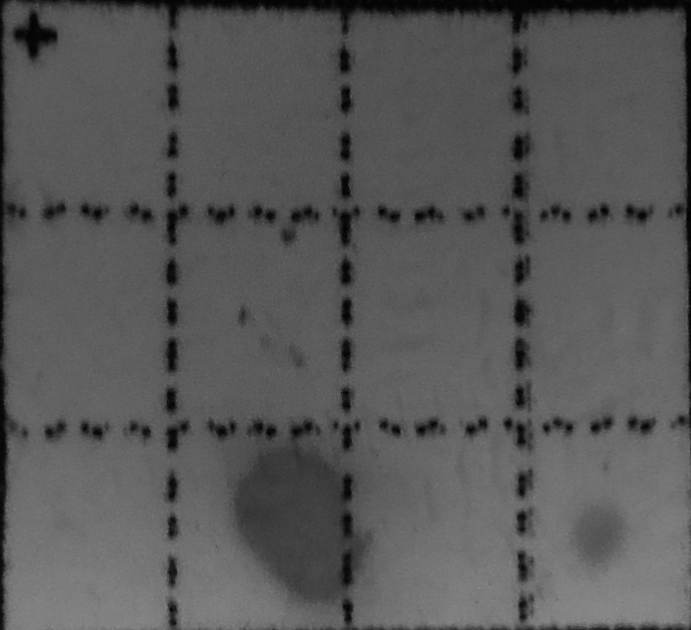

Supplement: Supplemental Information 1 [file peerj-11-15325-s001.zip › Raw Data/Results of 179 clinical samples of septicemia by membrane microarray (grayscale)-1/572409.tif]

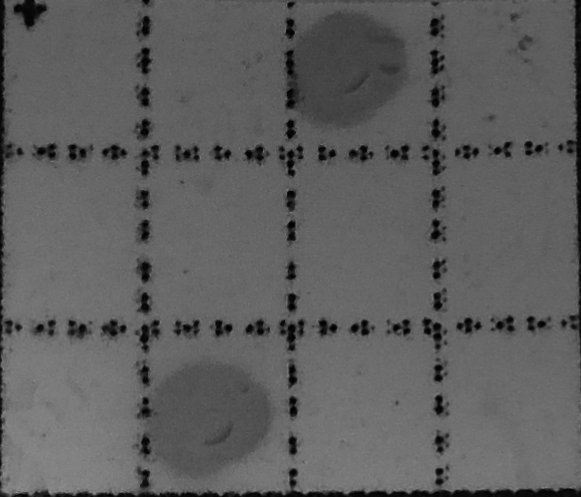

Supplement: Supplemental Information 1 [file peerj-11-15325-s001.zip › Raw Data/Results of 179 clinical samples of septicemia by membrane microarray (grayscale)-1/573116.tif]

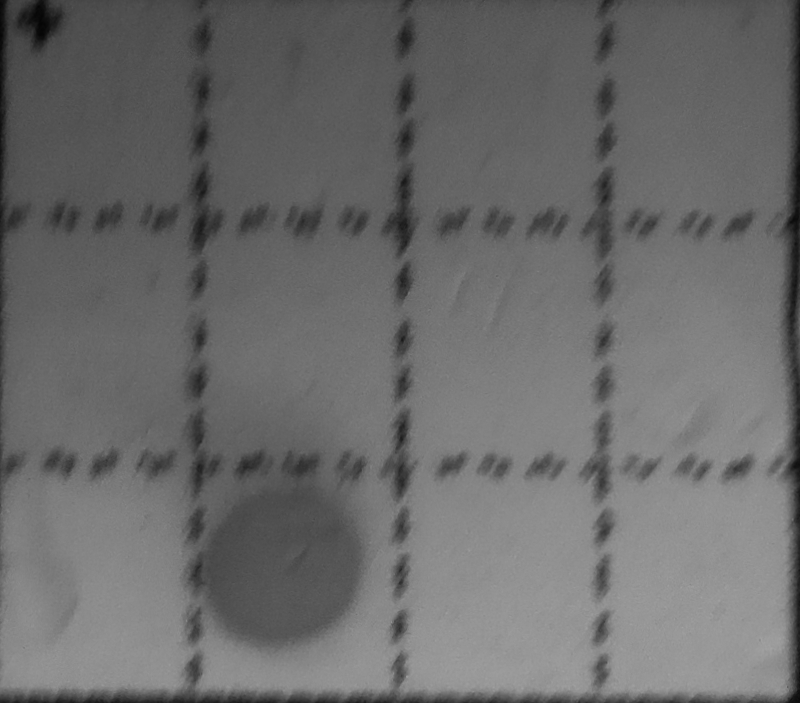

Supplement: Supplemental Information 1 [file peerj-11-15325-s001.zip › Raw Data/Results of 179 clinical samples of septicemia by membrane microarray (grayscale)-1/573429.tif]

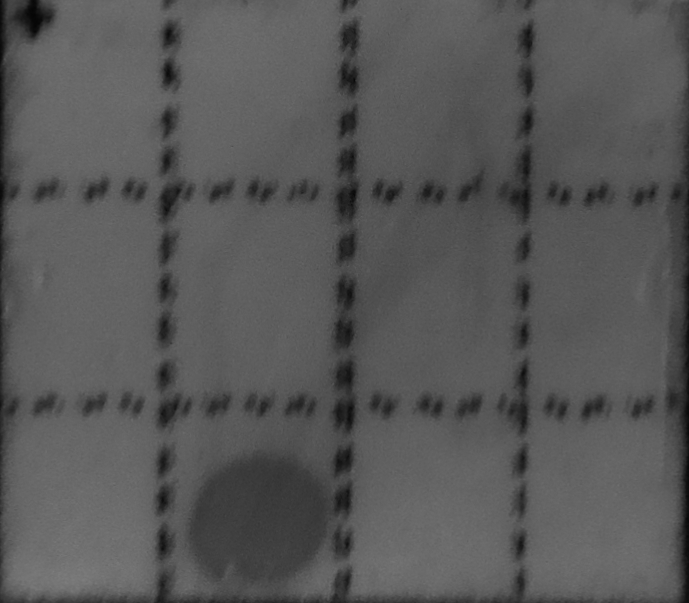

Supplement: Supplemental Information 1 [file peerj-11-15325-s001.zip › Raw Data/Results of 179 clinical samples of septicemia by membrane microarray (grayscale)-1/573701.tif]

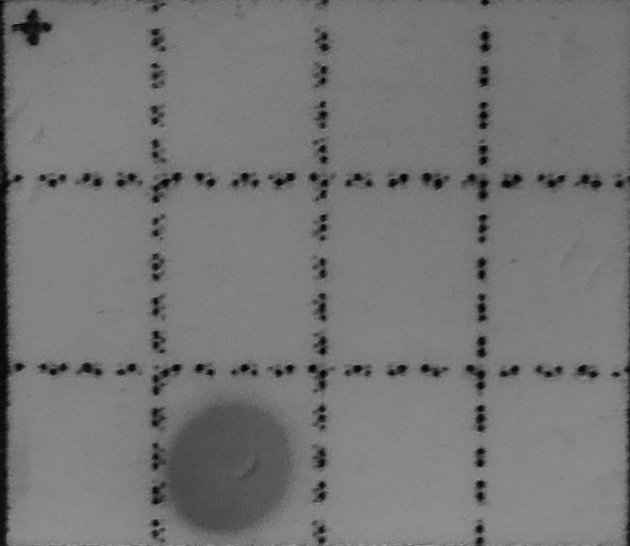

Supplement: Supplemental Information 1 [file peerj-11-15325-s001.zip › Raw Data/Results of 179 clinical samples of septicemia by membrane microarray (grayscale)-1/573741.tif]

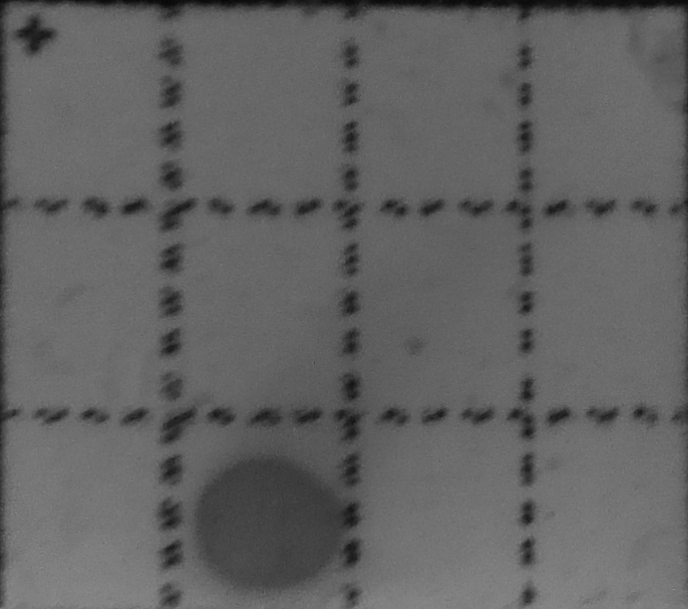

Supplement: Supplemental Information 1 [file peerj-11-15325-s001.zip › Raw Data/Results of 179 clinical samples of septicemia by membrane microarray (grayscale)-1/574108.tif]

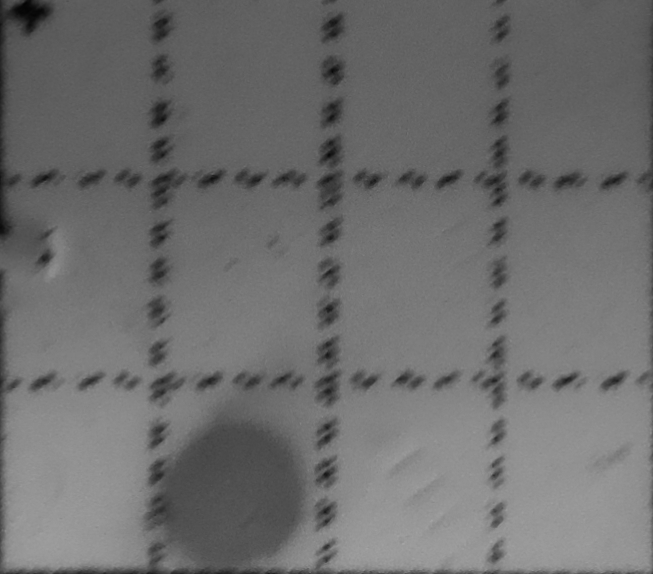

Supplement: Supplemental Information 1 [file peerj-11-15325-s001.zip › Raw Data/Results of 179 clinical samples of septicemia by membrane microarray (grayscale)-1/574503.tif]

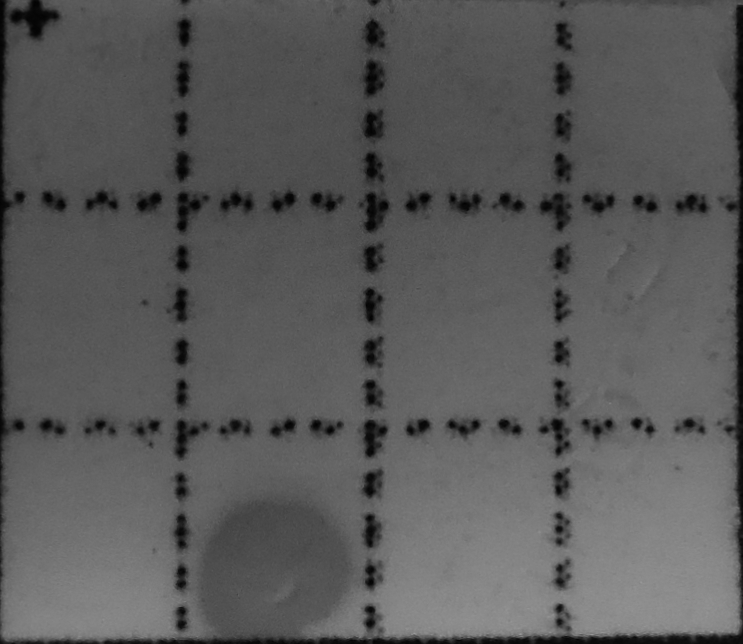

Supplement: Supplemental Information 1 [file peerj-11-15325-s001.zip › Raw Data/Results of 179 clinical samples of septicemia by membrane microarray (grayscale)-1/576048.tif]

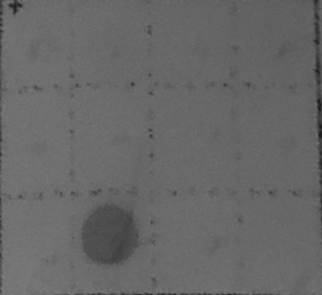

Supplement: Supplemental Information 1 [file peerj-11-15325-s001.zip › Raw Data/Results of 179 clinical samples of septicemia by membrane microarray (grayscale)-1/576657.tif]

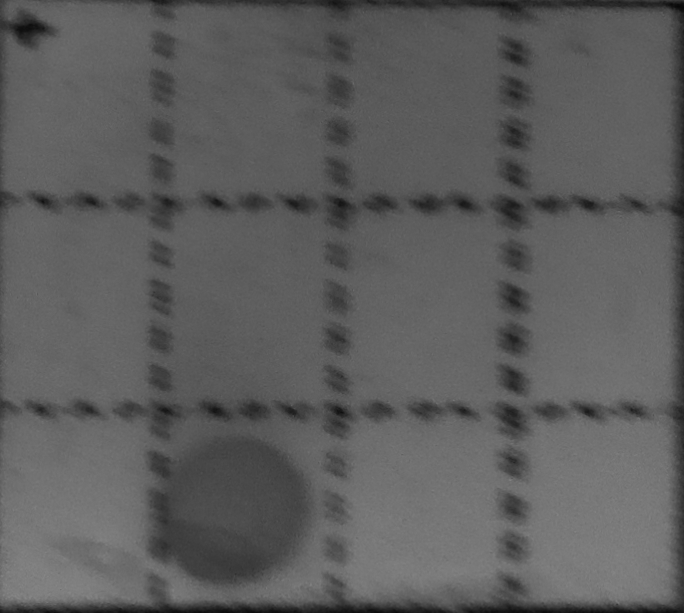

Supplement: Supplemental Information 1 [file peerj-11-15325-s001.zip › Raw Data/Results of 179 clinical samples of septicemia by membrane microarray (grayscale)-1/576742.tif]

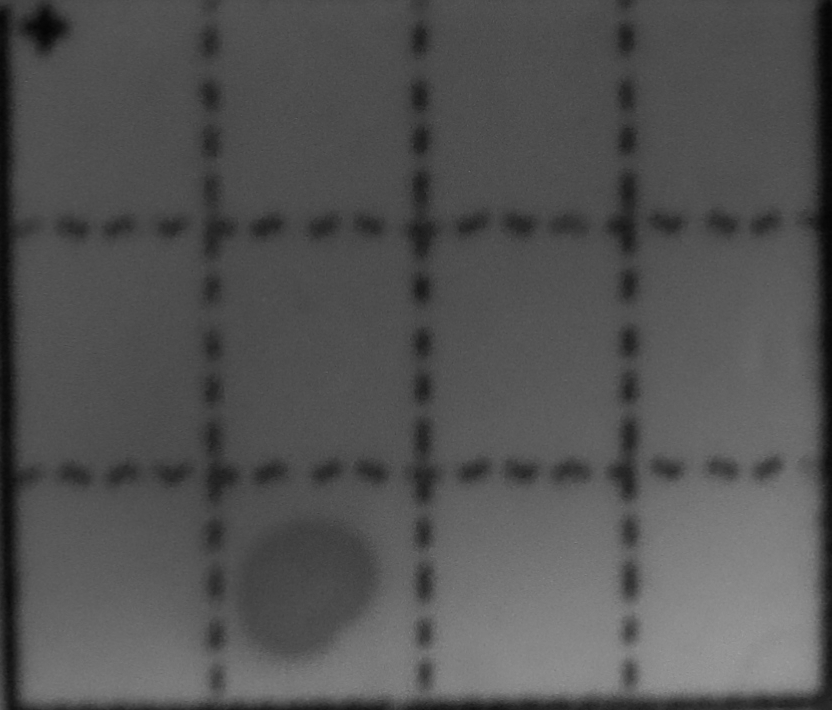

Supplement: Supplemental Information 1 [file peerj-11-15325-s001.zip › Raw Data/Results of 179 clinical samples of septicemia by membrane microarray (grayscale)-1/576815.tif]

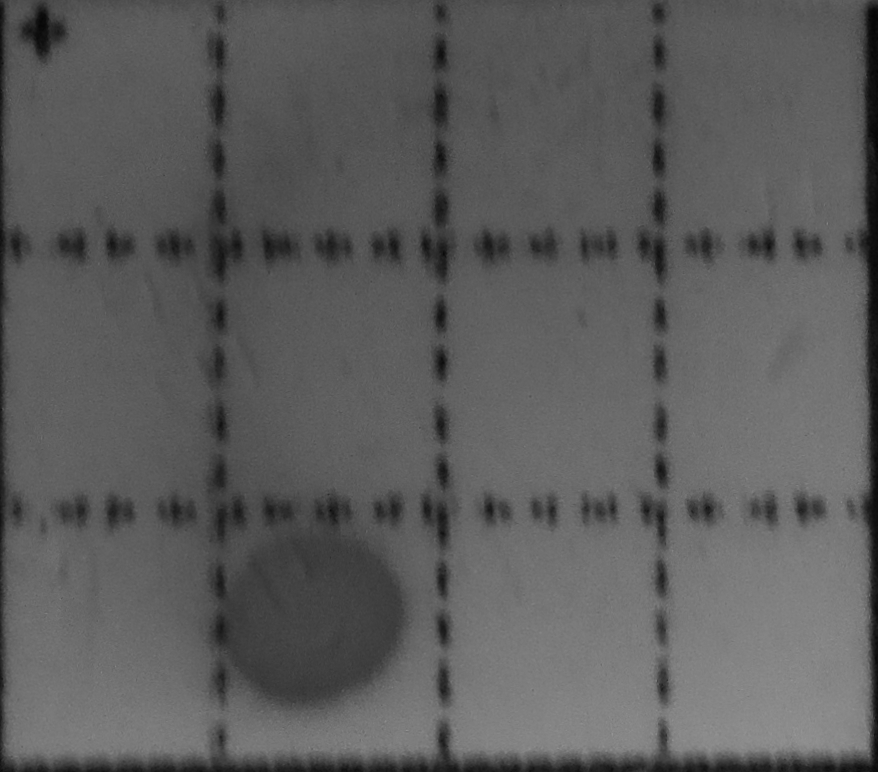

Supplement: Supplemental Information 1 [file peerj-11-15325-s001.zip › Raw Data/Results of 179 clinical samples of septicemia by membrane microarray (grayscale)-1/576869.tif]

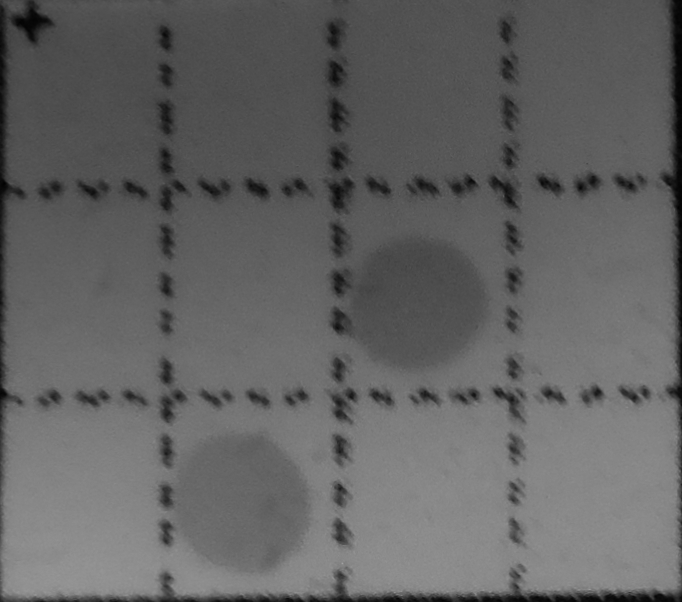

Supplement: Supplemental Information 1 [file peerj-11-15325-s001.zip › Raw Data/Results of 179 clinical samples of septicemia by membrane microarray (grayscale)-1/577754.tif]

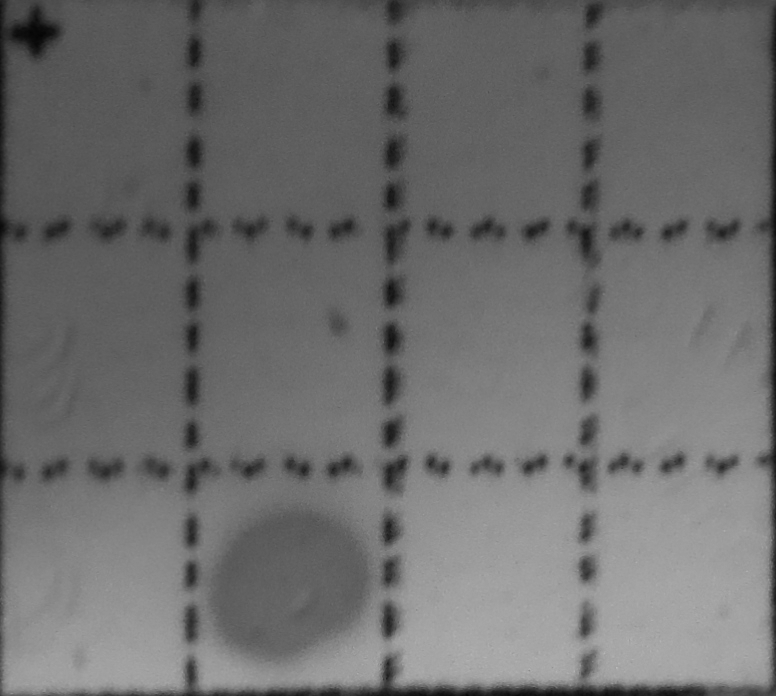

Supplement: Supplemental Information 1 [file peerj-11-15325-s001.zip › Raw Data/Results of 179 clinical samples of septicemia by membrane microarray (grayscale)-1/578603.tif]

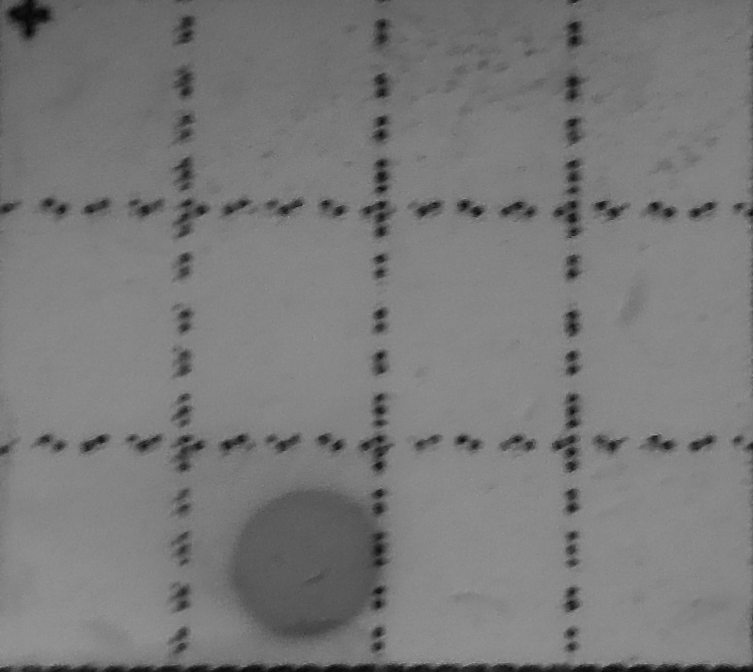

Supplement: Supplemental Information 1 [file peerj-11-15325-s001.zip › Raw Data/Results of 179 clinical samples of septicemia by membrane microarray (grayscale)-1/578651.tif]

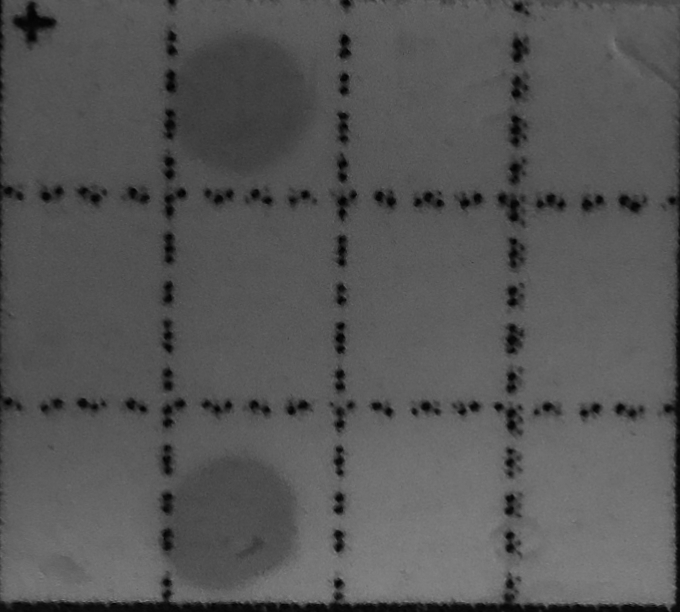

Supplement: Supplemental Information 1 [file peerj-11-15325-s001.zip › Raw Data/Results of 179 clinical samples of septicemia by membrane microarray (grayscale)-1/578692.tif]

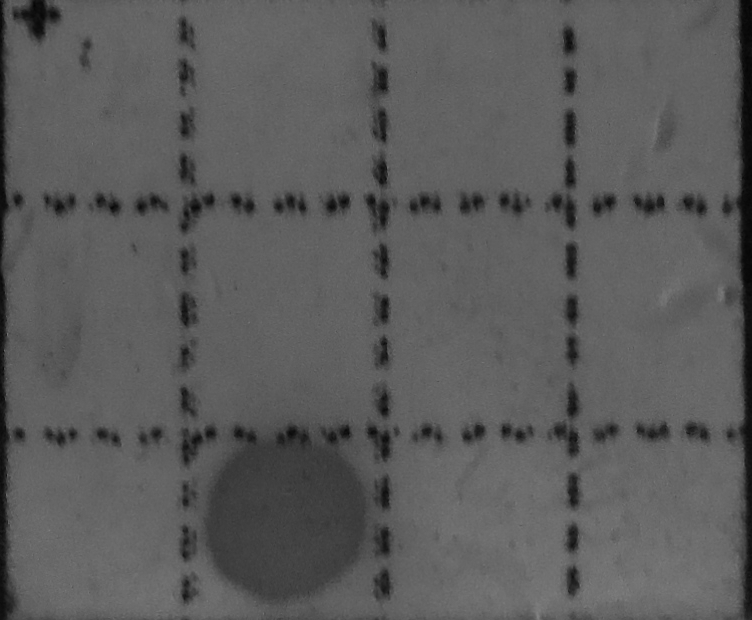

Supplement: Supplemental Information 1 [file peerj-11-15325-s001.zip › Raw Data/Results of 179 clinical samples of septicemia by membrane microarray (grayscale)-1/578816.tif]

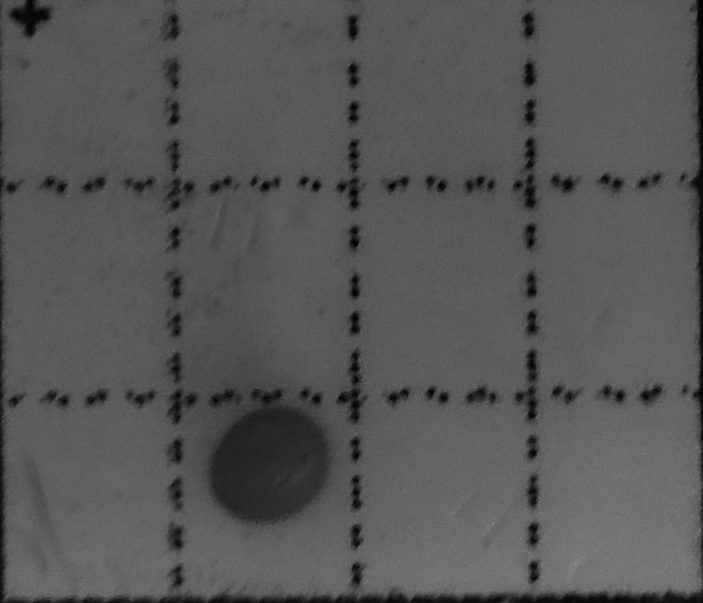

Supplement: Supplemental Information 1 [file peerj-11-15325-s001.zip › Raw Data/Results of 179 clinical samples of septicemia by membrane microarray (grayscale)-1/579254.tif]

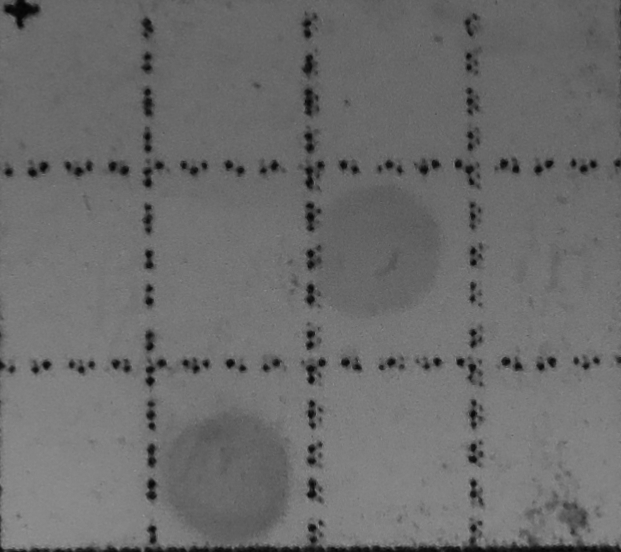

Supplement: Supplemental Information 1 [file peerj-11-15325-s001.zip › Raw Data/Results of 179 clinical samples of septicemia by membrane microarray (grayscale)-1/579729.tif]

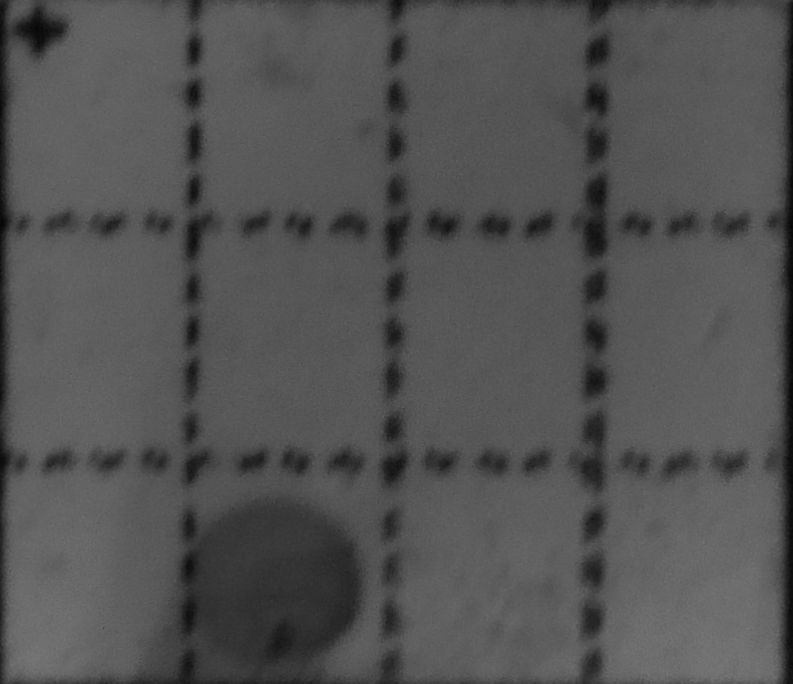

Supplement: Supplemental Information 1 [file peerj-11-15325-s001.zip › Raw Data/Results of 179 clinical samples of septicemia by membrane microarray (grayscale)-1/580060.tif]

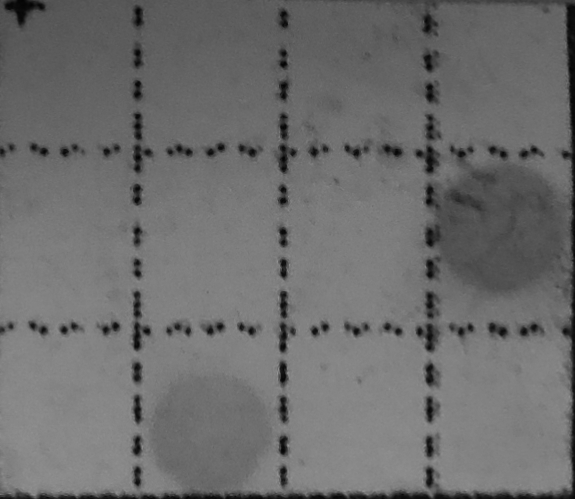

Supplement: Supplemental Information 1 [file peerj-11-15325-s001.zip › Raw Data/Results of 179 clinical samples of septicemia by membrane microarray (grayscale)-1/580802.tif]

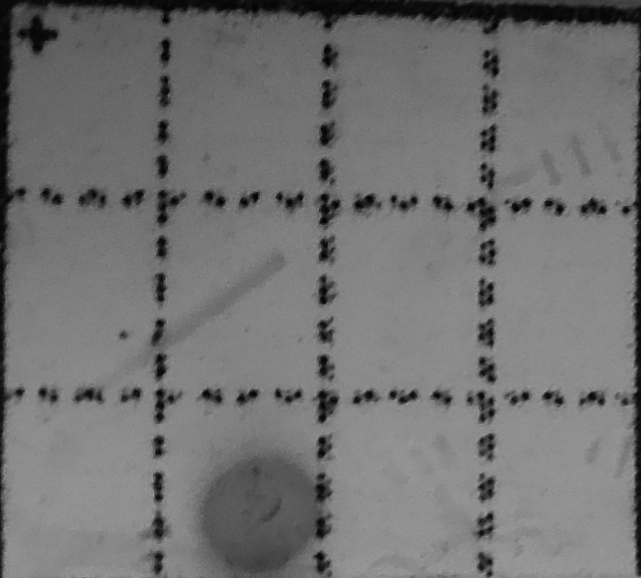

Supplement: Supplemental Information 1 [file peerj-11-15325-s001.zip › Raw Data/Results of 179 clinical samples of septicemia by membrane microarray (grayscale)-1/581311.tif]

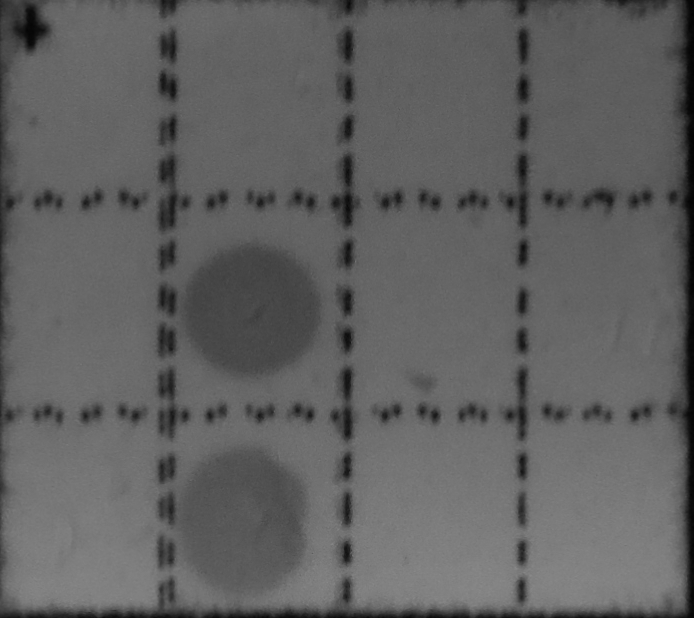

Supplement: Supplemental Information 1 [file peerj-11-15325-s001.zip › Raw Data/Results of 179 clinical samples of septicemia by membrane microarray (grayscale)-1/582268.tif]

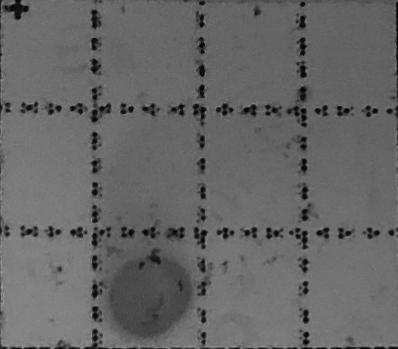

Supplement: Supplemental Information 1 [file peerj-11-15325-s001.zip › Raw Data/Results of 179 clinical samples of septicemia by membrane microarray (grayscale)-1/582702.tif]

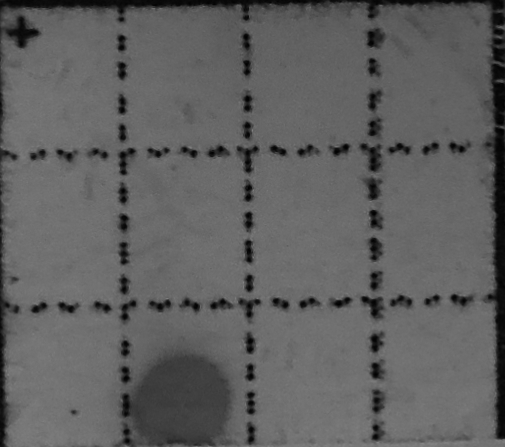

Supplement: Supplemental Information 1 [file peerj-11-15325-s001.zip › Raw Data/Results of 179 clinical samples of septicemia by membrane microarray (grayscale)-1/582740.tif]

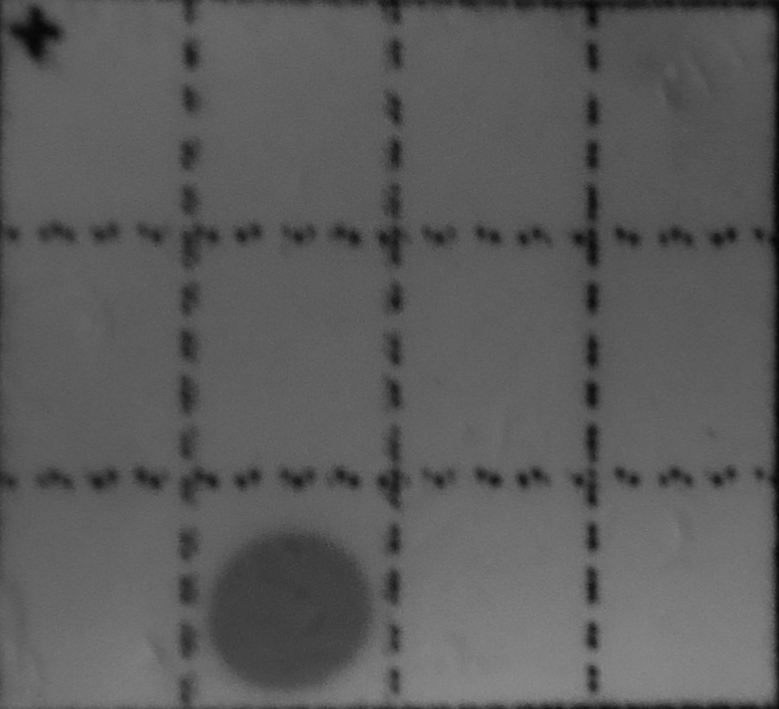

Supplement: Supplemental Information 1 [file peerj-11-15325-s001.zip › Raw Data/Results of 179 clinical samples of septicemia by membrane microarray (grayscale)-1/582797.tif]

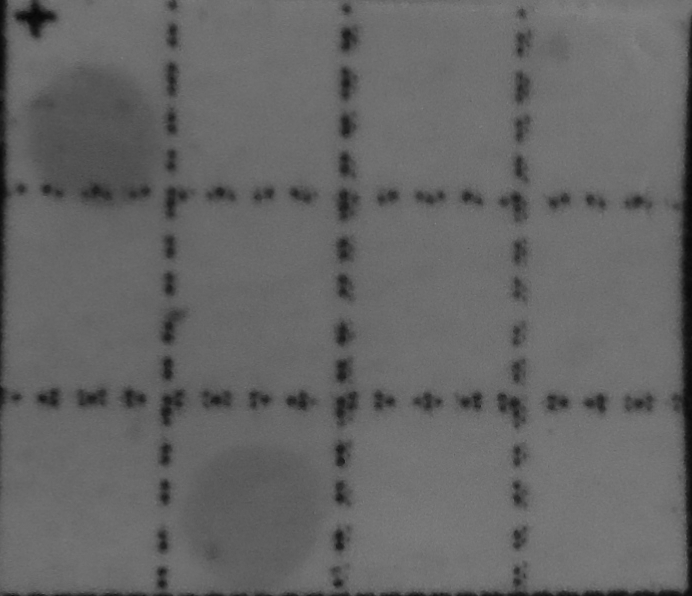

Supplement: Supplemental Information 1 [file peerj-11-15325-s001.zip › Raw Data/Results of 179 clinical samples of septicemia by membrane microarray (grayscale)-1/584063.tif]

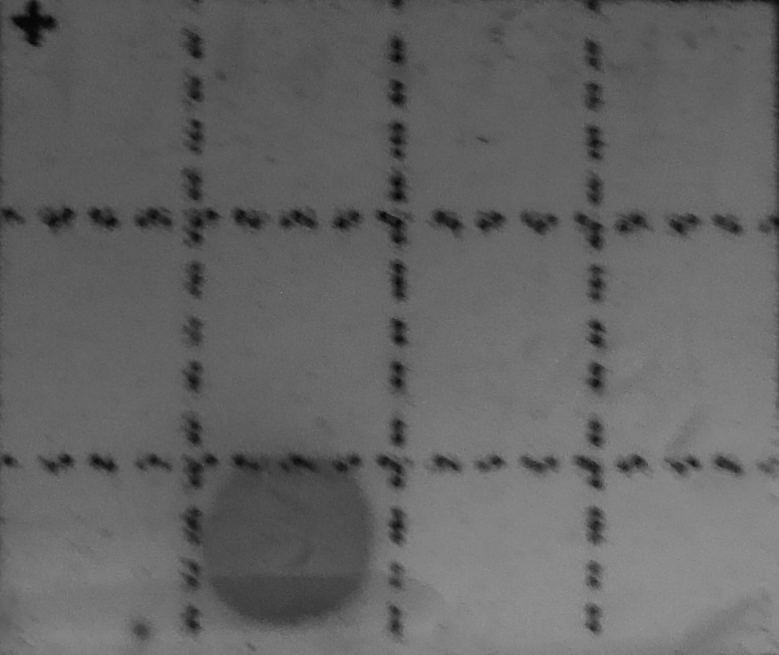

Supplement: Supplemental Information 1 [file peerj-11-15325-s001.zip › Raw Data/Results of 179 clinical samples of septicemia by membrane microarray (grayscale)-1/584368.tif]

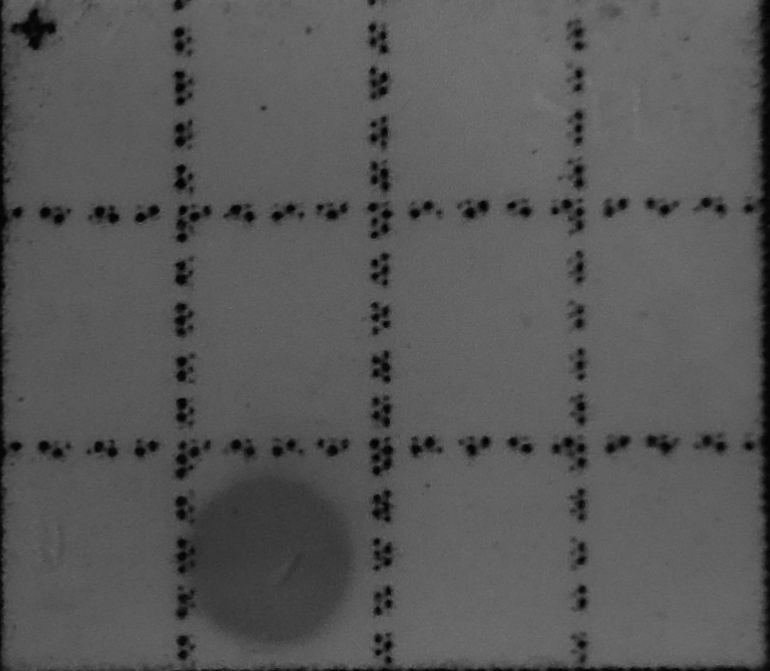

Supplement: Supplemental Information 1 [file peerj-11-15325-s001.zip › Raw Data/Results of 179 clinical samples of septicemia by membrane microarray (grayscale)-1/584502.tif]

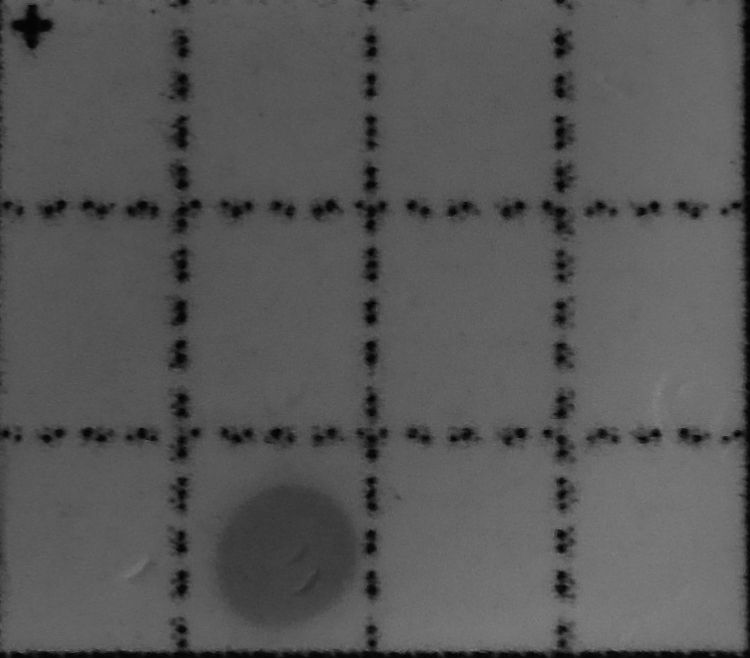

Supplement: Supplemental Information 1 [file peerj-11-15325-s001.zip › Raw Data/Results of 179 clinical samples of septicemia by membrane microarray (grayscale)-1/585791.tif]

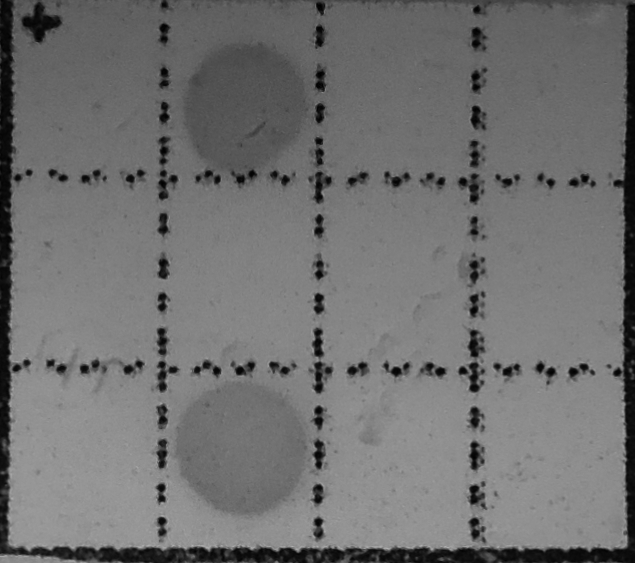

Supplement: Supplemental Information 1 [file peerj-11-15325-s001.zip › Raw Data/Results of 179 clinical samples of septicemia by membrane microarray (grayscale)-1/585866.tif]

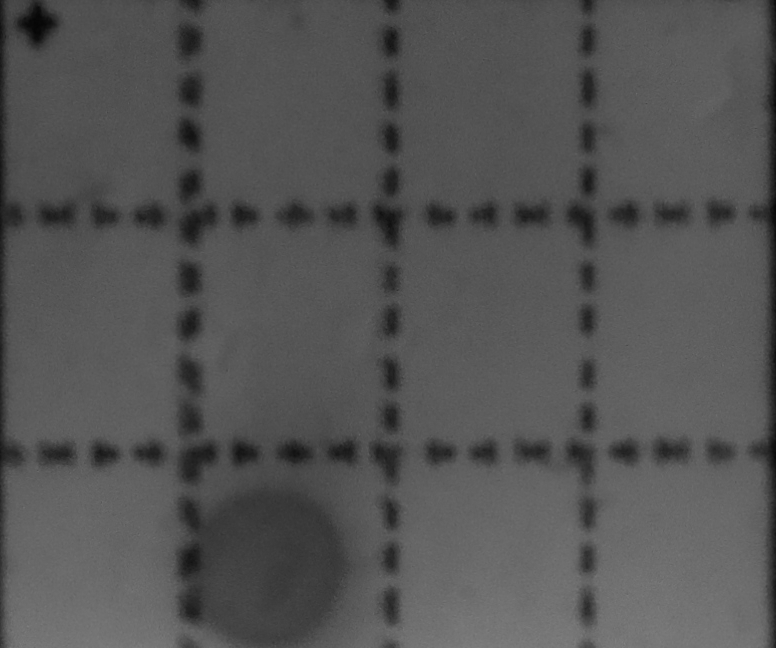

Supplement: Supplemental Information 1 [file peerj-11-15325-s001.zip › Raw Data/Results of 179 clinical samples of septicemia by membrane microarray (grayscale)-1/586552.tif]

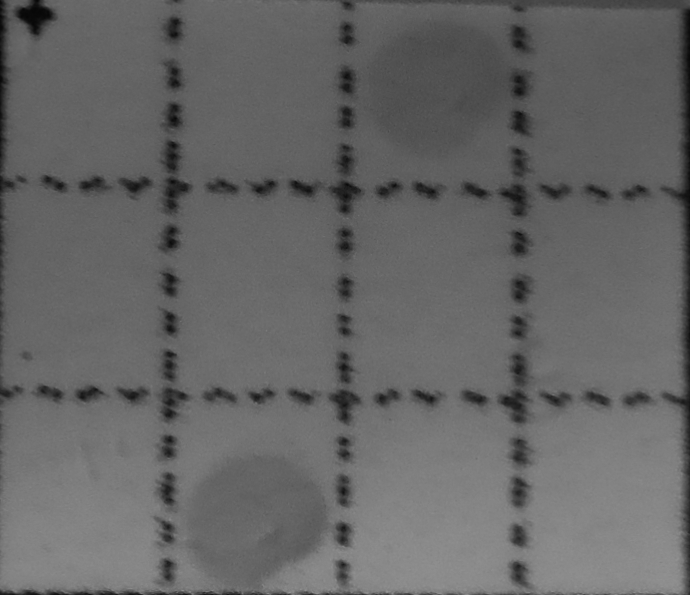

Supplement: Supplemental Information 1 [file peerj-11-15325-s001.zip › Raw Data/Results of 179 clinical samples of septicemia by membrane microarray (grayscale)-1/587392.tif]

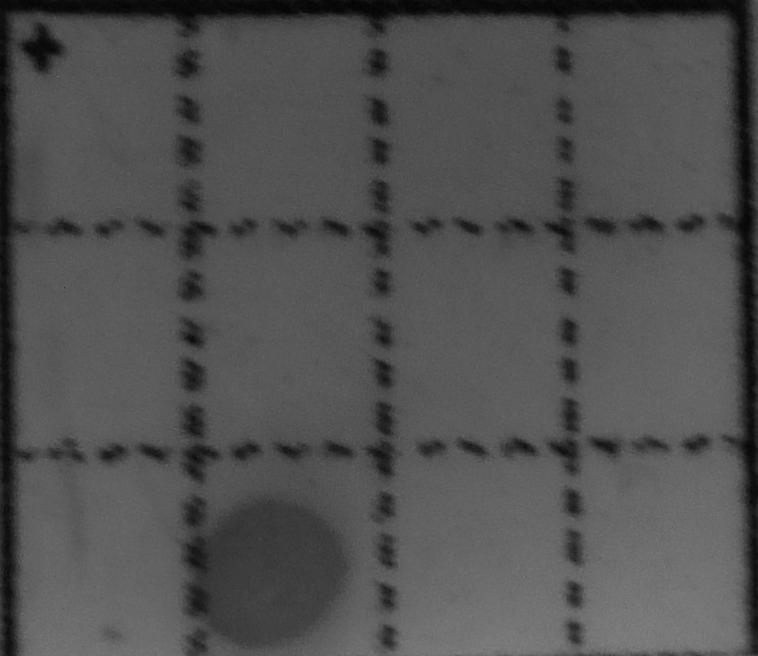

Supplement: Supplemental Information 1 [file peerj-11-15325-s001.zip › Raw Data/Results of 179 clinical samples of septicemia by membrane microarray (grayscale)-1/587531.tif]

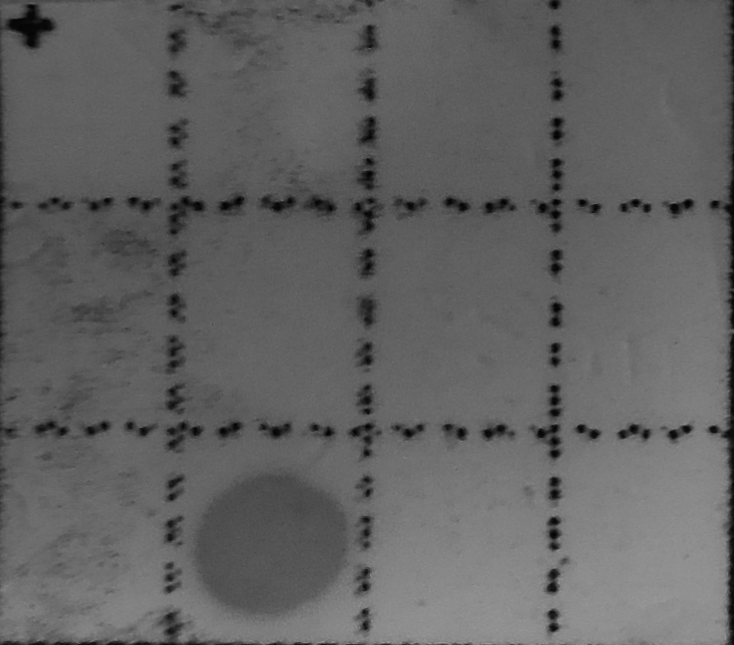

Supplement: Supplemental Information 1 [file peerj-11-15325-s001.zip › Raw Data/Results of 179 clinical samples of septicemia by membrane microarray (grayscale)-1/588003.tif]

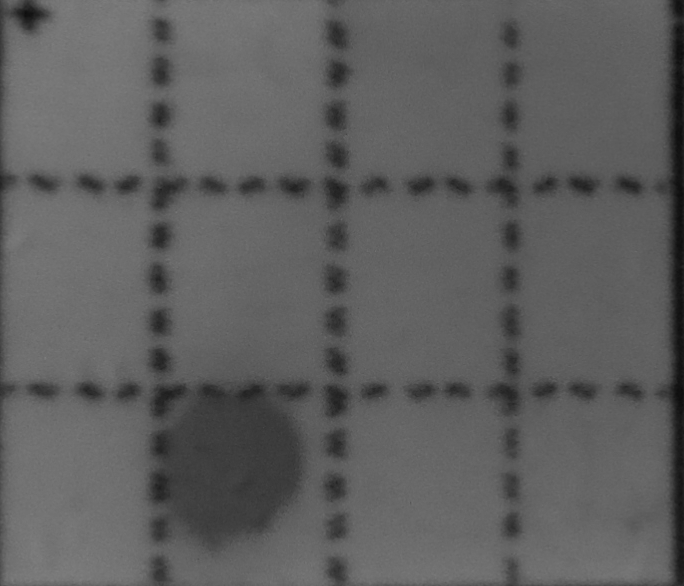

Supplement: Supplemental Information 1 [file peerj-11-15325-s001.zip › Raw Data/Results of 179 clinical samples of septicemia by membrane microarray (grayscale)-1/588227.tif]

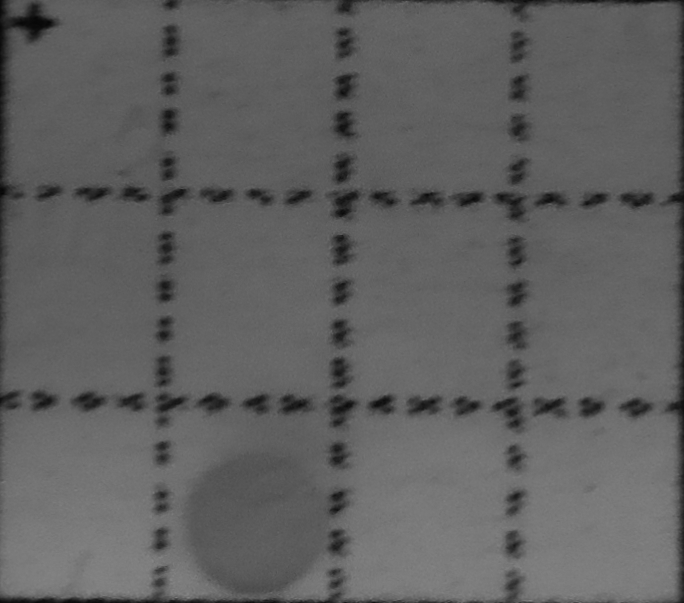

Supplement: Supplemental Information 1 [file peerj-11-15325-s001.zip › Raw Data/Results of 179 clinical samples of septicemia by membrane microarray (grayscale)-1/589143.tif]

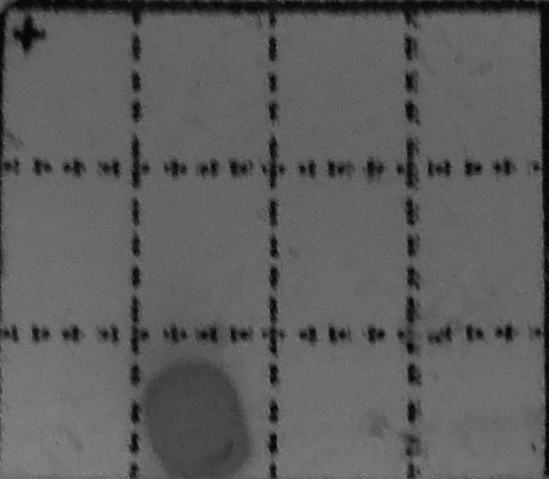

Supplement: Supplemental Information 1 [file peerj-11-15325-s001.zip › Raw Data/Results of 179 clinical samples of septicemia by membrane microarray (grayscale)-1/590933.tif]

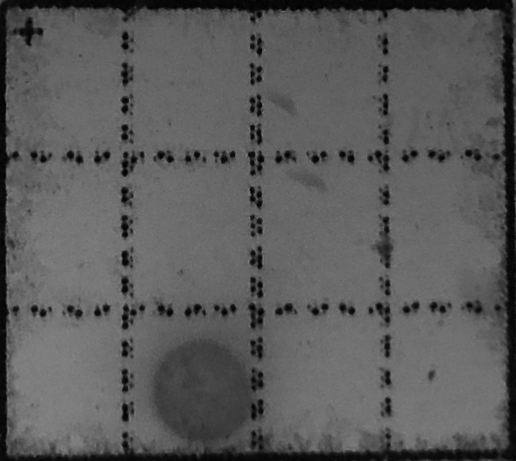

Supplement: Supplemental Information 1 [file peerj-11-15325-s001.zip › Raw Data/Results of 179 clinical samples of septicemia by membrane microarray (grayscale)-1/591502.tif]

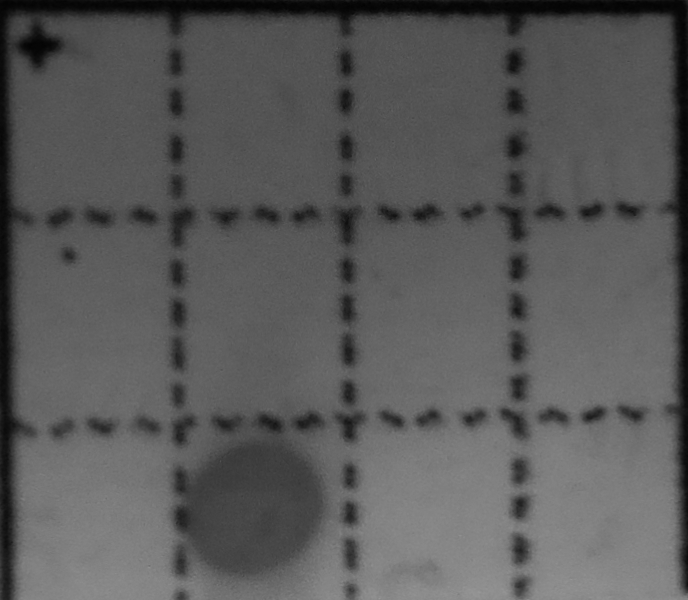

Supplement: Supplemental Information 1 [file peerj-11-15325-s001.zip › Raw Data/Results of 179 clinical samples of septicemia by membrane microarray (grayscale)-1/591850.tif]

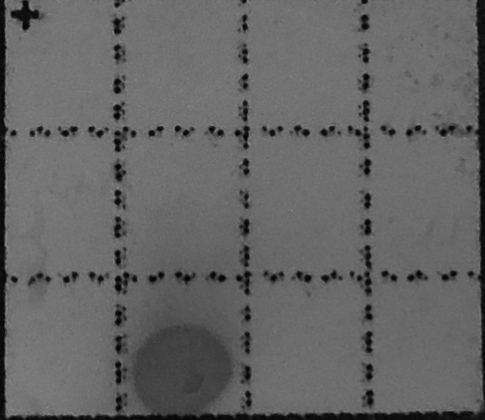

Supplement: Supplemental Information 1 [file peerj-11-15325-s001.zip › Raw Data/Results of 179 clinical samples of septicemia by membrane microarray (grayscale)-1/592121.tif]

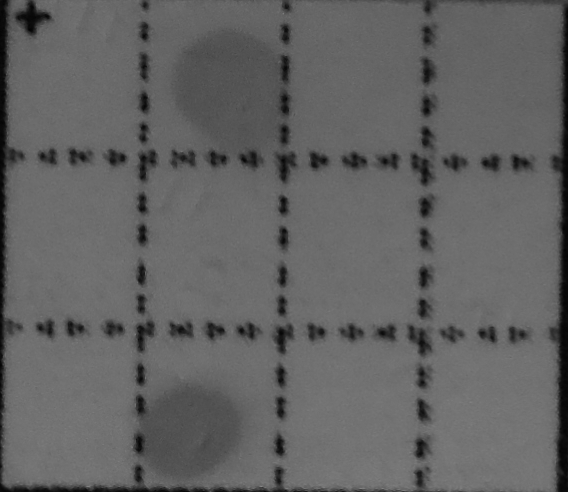

Supplement: Supplemental Information 1 [file peerj-11-15325-s001.zip › Raw Data/Results of 179 clinical samples of septicemia by membrane microarray (grayscale)-1/592290.tif]

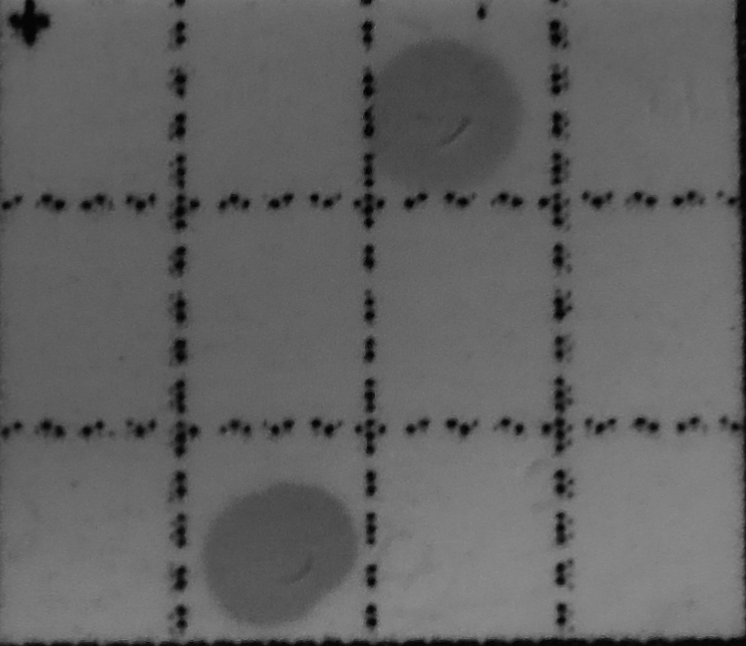

Supplement: Supplemental Information 1 [file peerj-11-15325-s001.zip › Raw Data/Results of 179 clinical samples of septicemia by membrane microarray (grayscale)-1/592536.tif]

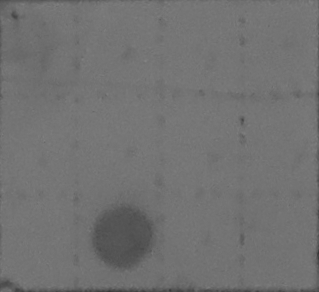

Supplement: Supplemental Information 1 [file peerj-11-15325-s001.zip › Raw Data/Results of 179 clinical samples of septicemia by membrane microarray (grayscale)-1/592705.tif]

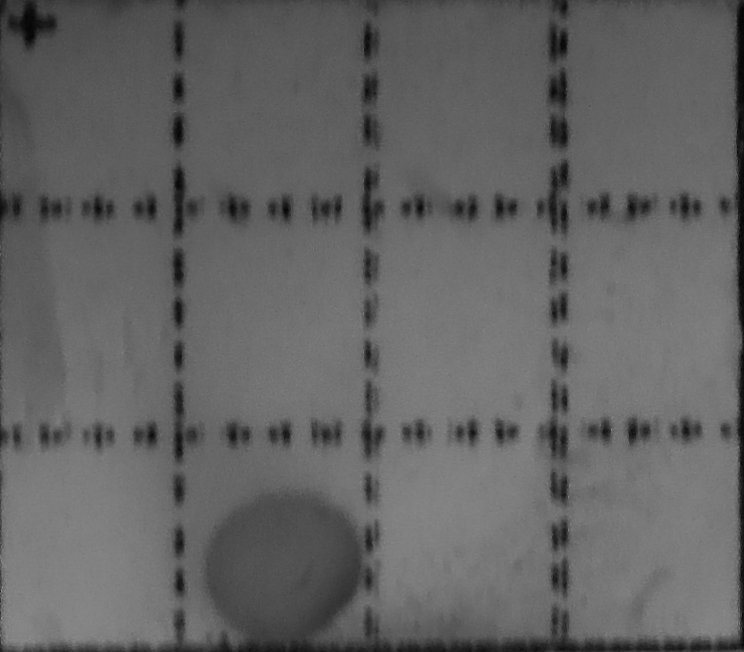

Supplement: Supplemental Information 1 [file peerj-11-15325-s001.zip › Raw Data/Results of 179 clinical samples of septicemia by membrane microarray (grayscale)-1/592773.tif]

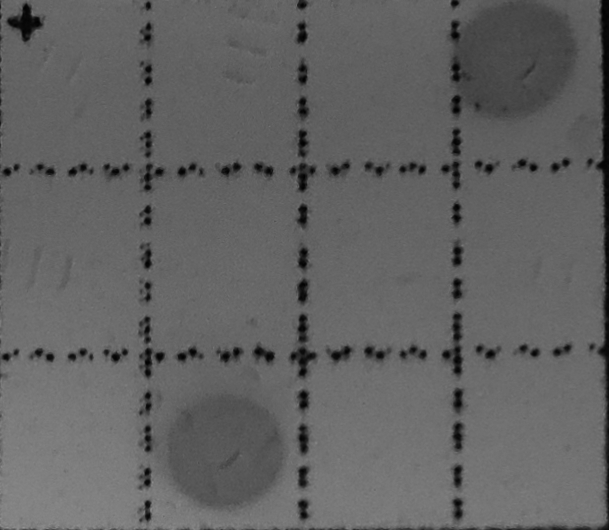

Supplement: Supplemental Information 1 [file peerj-11-15325-s001.zip › Raw Data/Results of 179 clinical samples of septicemia by membrane microarray (grayscale)-1/592800.tif]

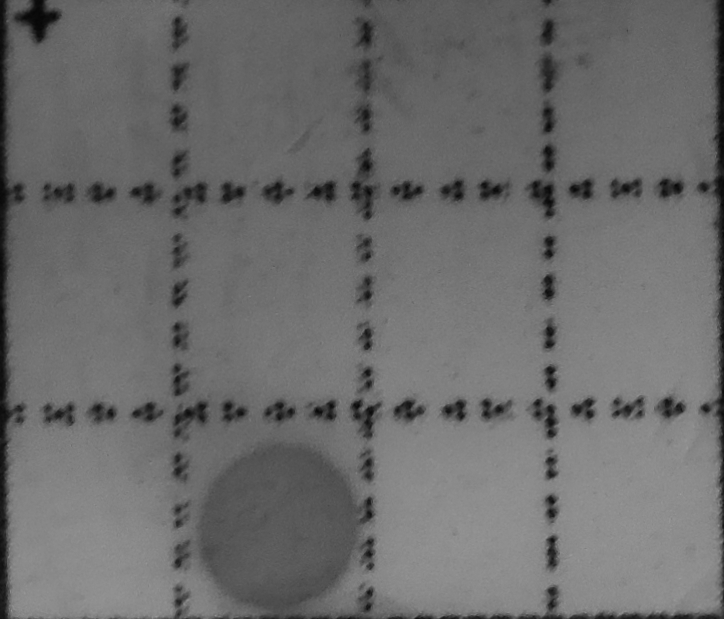

Supplement: Supplemental Information 1 [file peerj-11-15325-s001.zip › Raw Data/Results of 179 clinical samples of septicemia by membrane microarray (grayscale)-1/593689.tif]

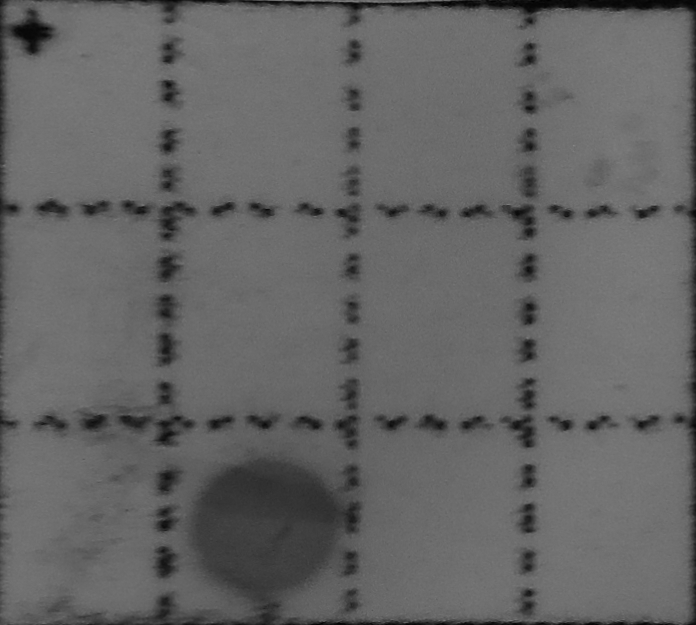

Supplement: Supplemental Information 1 [file peerj-11-15325-s001.zip › Raw Data/Results of 179 clinical samples of septicemia by membrane microarray (grayscale)-1/593914.tif]

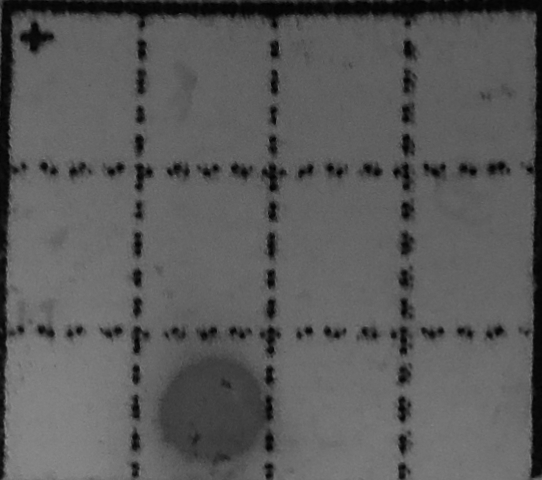

Supplement: Supplemental Information 1 [file peerj-11-15325-s001.zip › Raw Data/Results of 179 clinical samples of septicemia by membrane microarray (grayscale)-1/595301.tif]

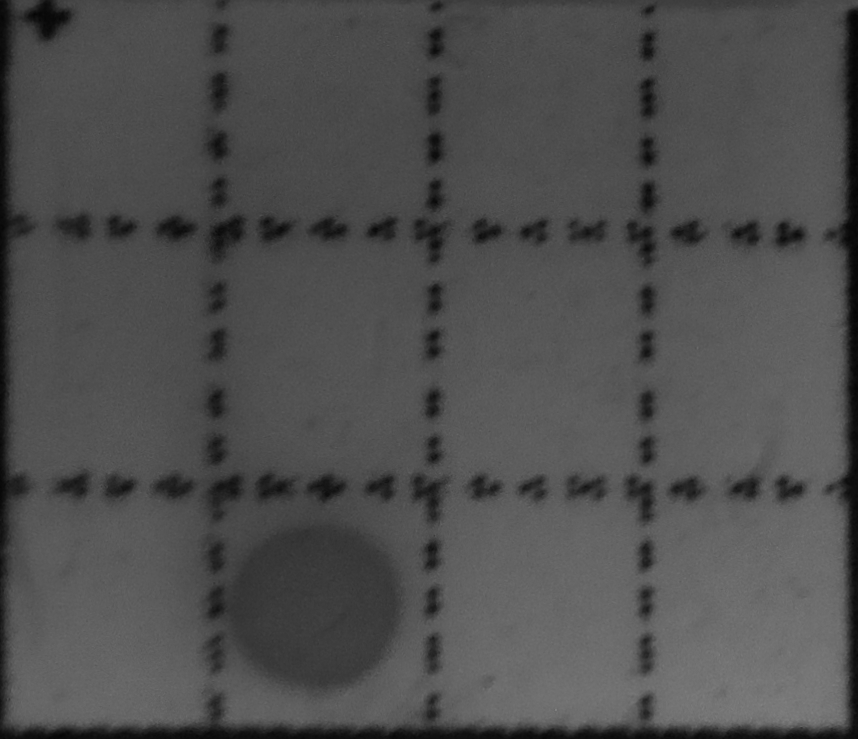

Supplement: Supplemental Information 1 [file peerj-11-15325-s001.zip › Raw Data/Results of 179 clinical samples of septicemia by membrane microarray (grayscale)-1/597146.tif]

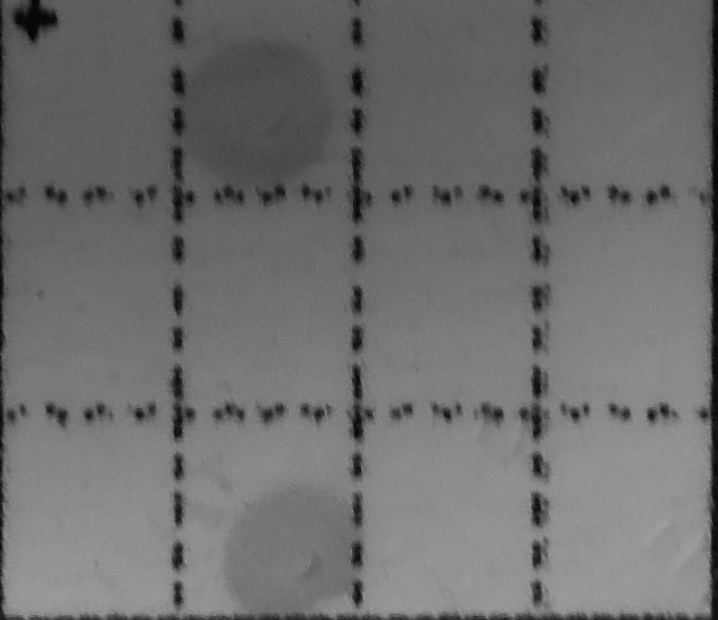

Supplement: Supplemental Information 1 [file peerj-11-15325-s001.zip › Raw Data/Results of 179 clinical samples of septicemia by membrane microarray (grayscale)-1/597339.tif]

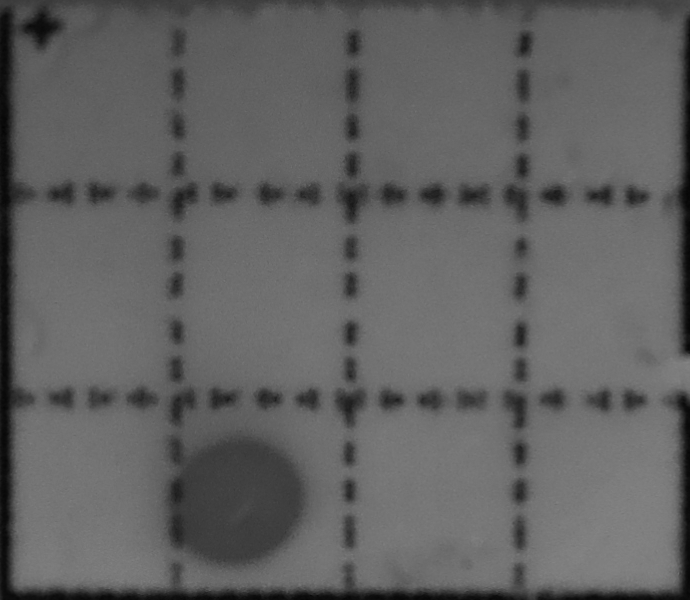

Supplement: Supplemental Information 1 [file peerj-11-15325-s001.zip › Raw Data/Results of 179 clinical samples of septicemia by membrane microarray (grayscale)-1/597971.tif]

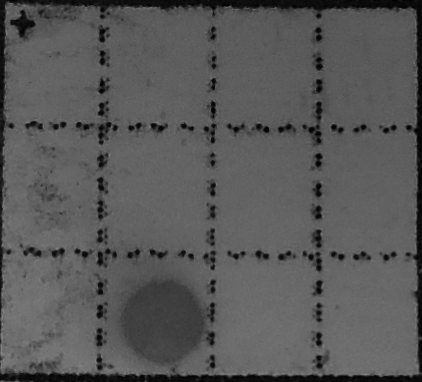

Supplement: Supplemental Information 1 [file peerj-11-15325-s001.zip › Raw Data/Results of 179 clinical samples of septicemia by membrane microarray (grayscale)-1/598393.tif]

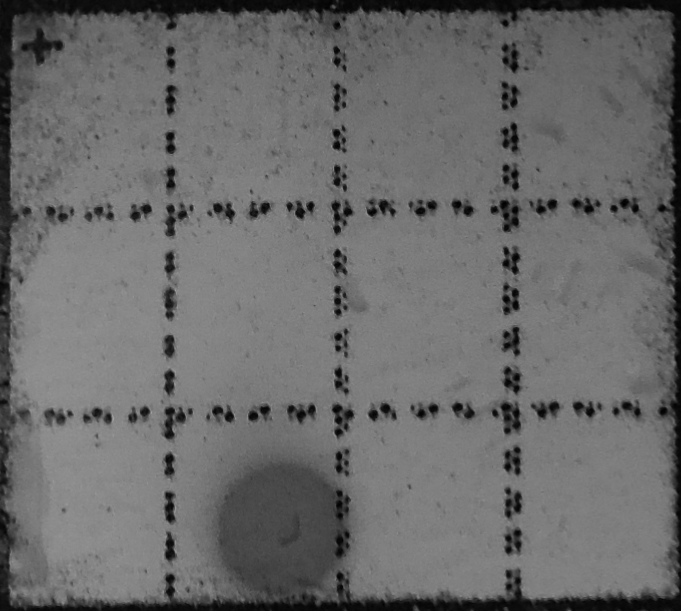

Supplement: Supplemental Information 1 [file peerj-11-15325-s001.zip › Raw Data/Results of 179 clinical samples of septicemia by membrane microarray (grayscale)-1/598819.tif]

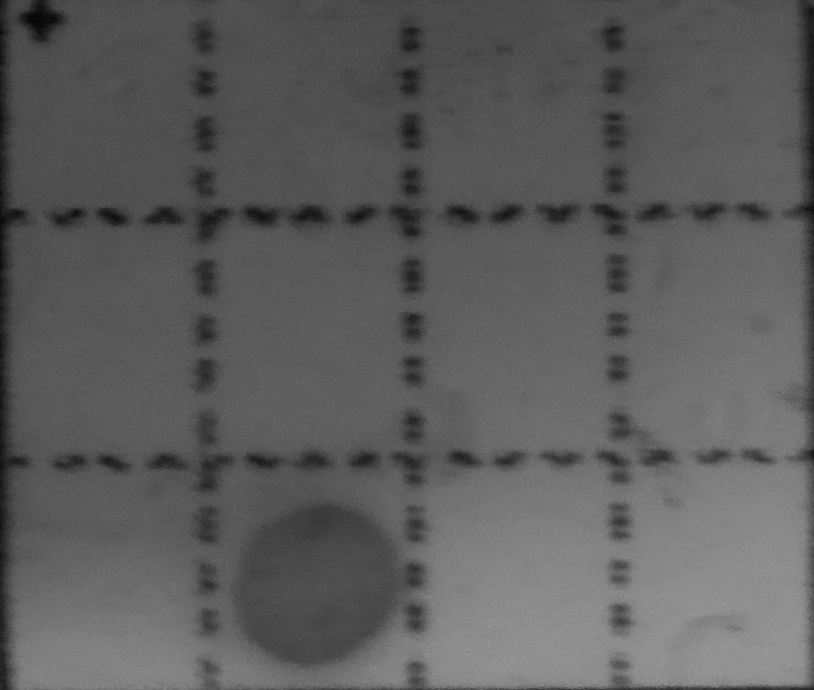

Supplement: Supplemental Information 1 [file peerj-11-15325-s001.zip › Raw Data/Results of 179 clinical samples of septicemia by membrane microarray (grayscale)-1/598964.tif]

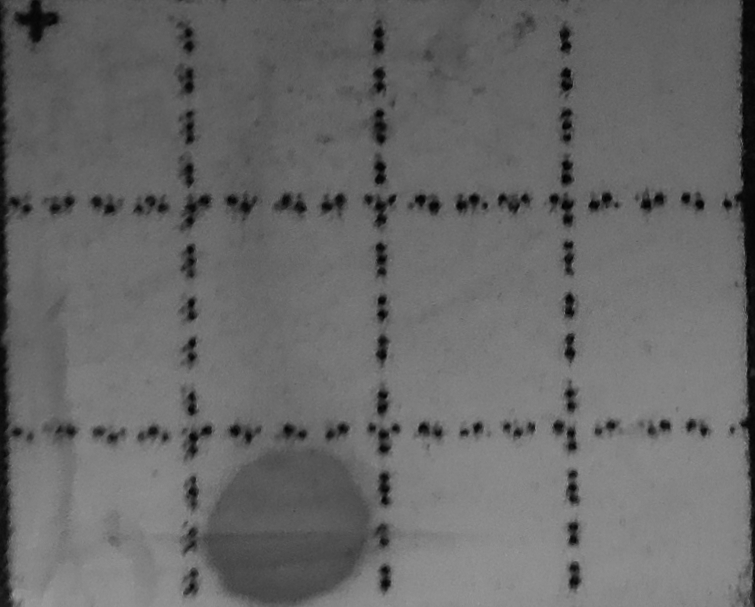

Supplement: Supplemental Information 1 [file peerj-11-15325-s001.zip › Raw Data/Results of 179 clinical samples of septicemia by membrane microarray (grayscale)-1/599257.tif]

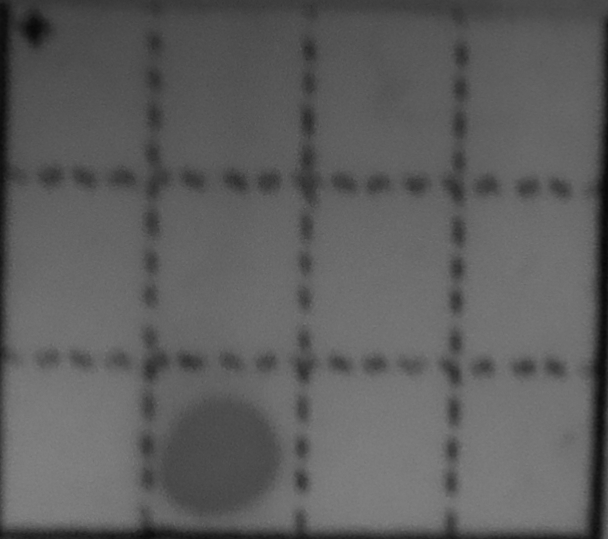

Supplement: Supplemental Information 1 [file peerj-11-15325-s001.zip › Raw Data/Results of 179 clinical samples of septicemia by membrane microarray (grayscale)-1/599975.tif]

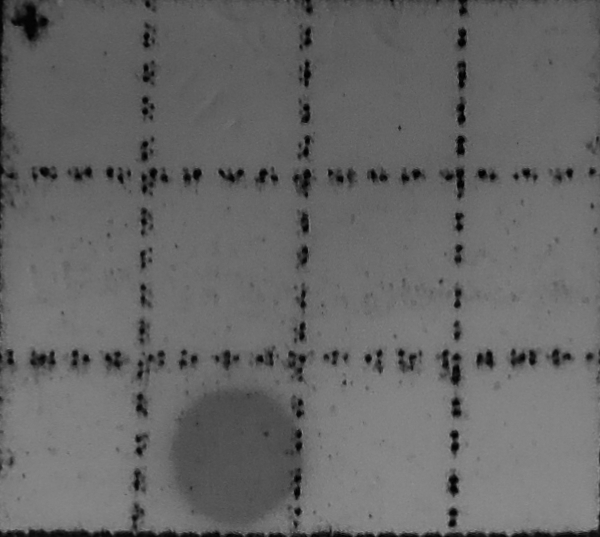

Supplement: Supplemental Information 1 [file peerj-11-15325-s001.zip › Raw Data/Results of 179 clinical samples of septicemia by membrane microarray (grayscale)-1/600157.tif]

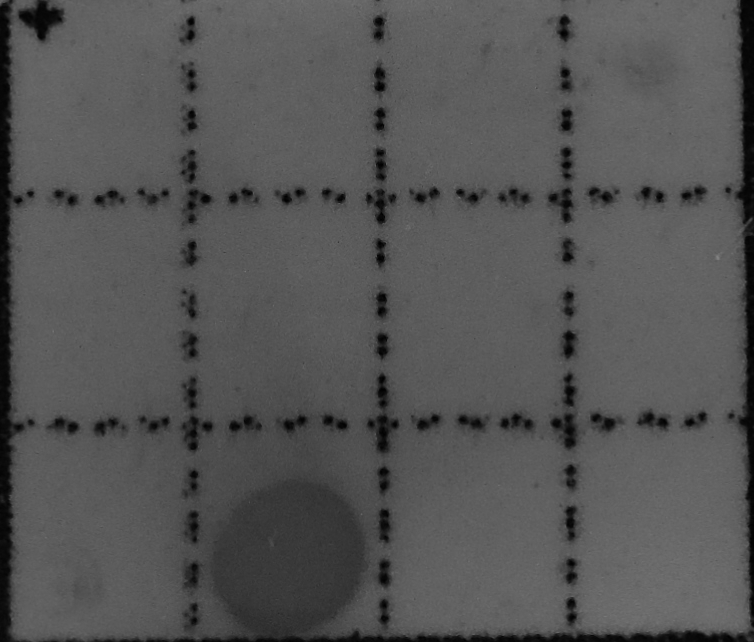

Supplement: Supplemental Information 1 [file peerj-11-15325-s001.zip › Raw Data/Results of 179 clinical samples of septicemia by membrane microarray (grayscale)-1/600194.tif]

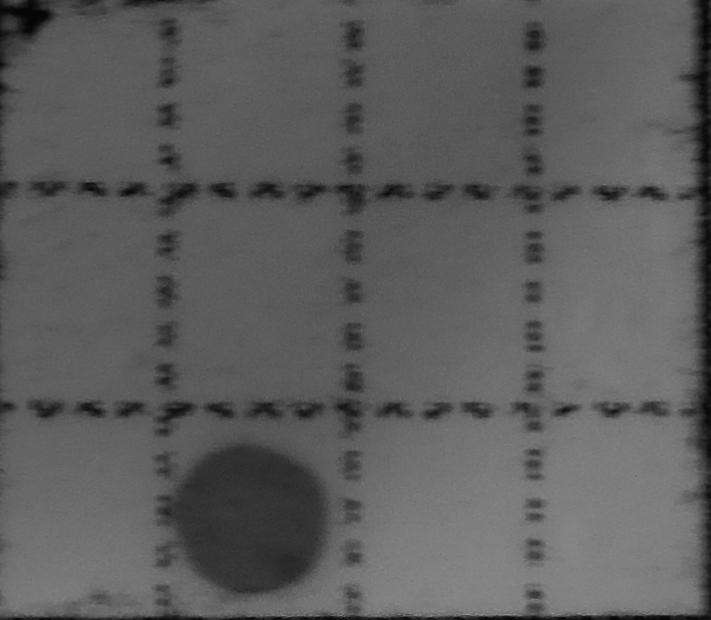

Supplement: Supplemental Information 1 [file peerj-11-15325-s001.zip › Raw Data/Results of 179 clinical samples of septicemia by membrane microarray (grayscale)-1/600264.tif]

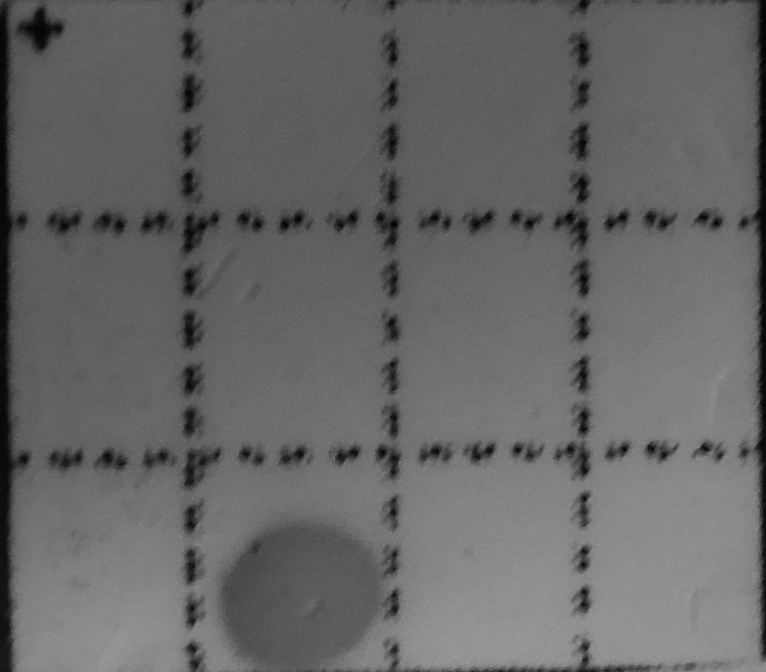

Supplement: Supplemental Information 1 [file peerj-11-15325-s001.zip › Raw Data/Results of 179 clinical samples of septicemia by membrane microarray (grayscale)-1/600436.tif]

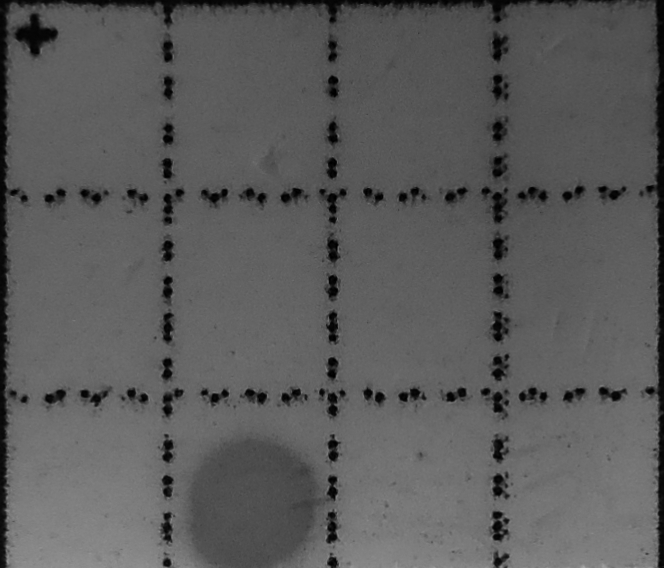

Supplement: Supplemental Information 1 [file peerj-11-15325-s001.zip › Raw Data/Results of 179 clinical samples of septicemia by membrane microarray (grayscale)-1/601138.tif]

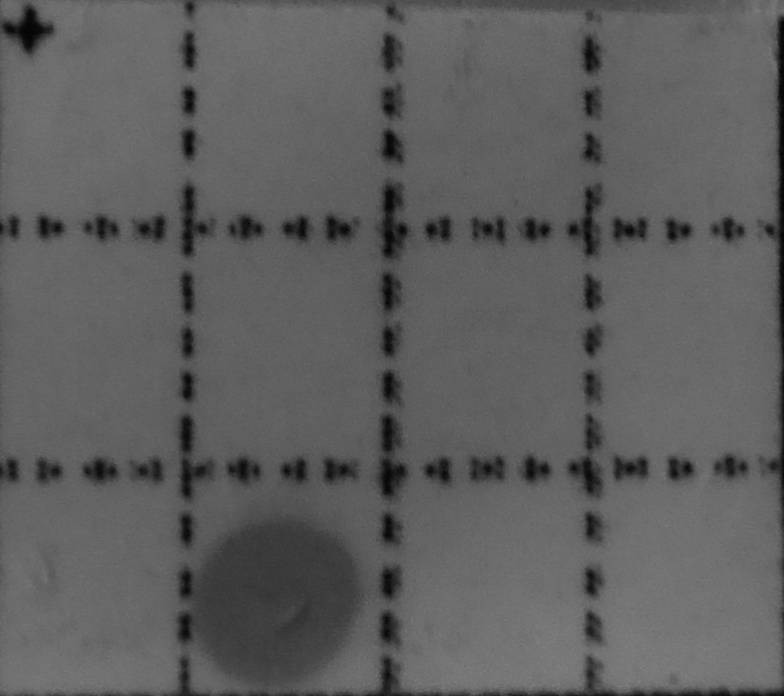

Supplement: Supplemental Information 1 [file peerj-11-15325-s001.zip › Raw Data/Results of 179 clinical samples of septicemia by membrane microarray (grayscale)-1/601621.tif]

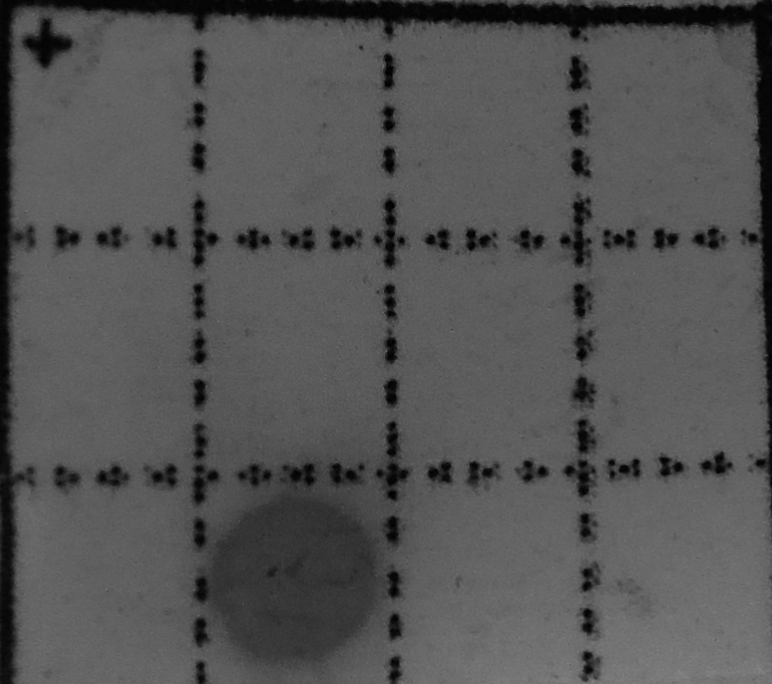

Supplement: Supplemental Information 1 [file peerj-11-15325-s001.zip › Raw Data/Results of 179 clinical samples of septicemia by membrane microarray (grayscale)-1/601991.tif]

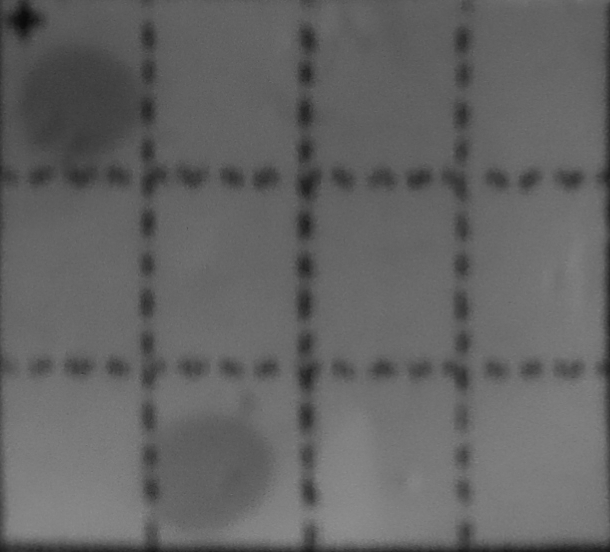

Supplement: Supplemental Information 1 [file peerj-11-15325-s001.zip › Raw Data/Results of 179 clinical samples of septicemia by membrane microarray (grayscale)-1/602261.tif]

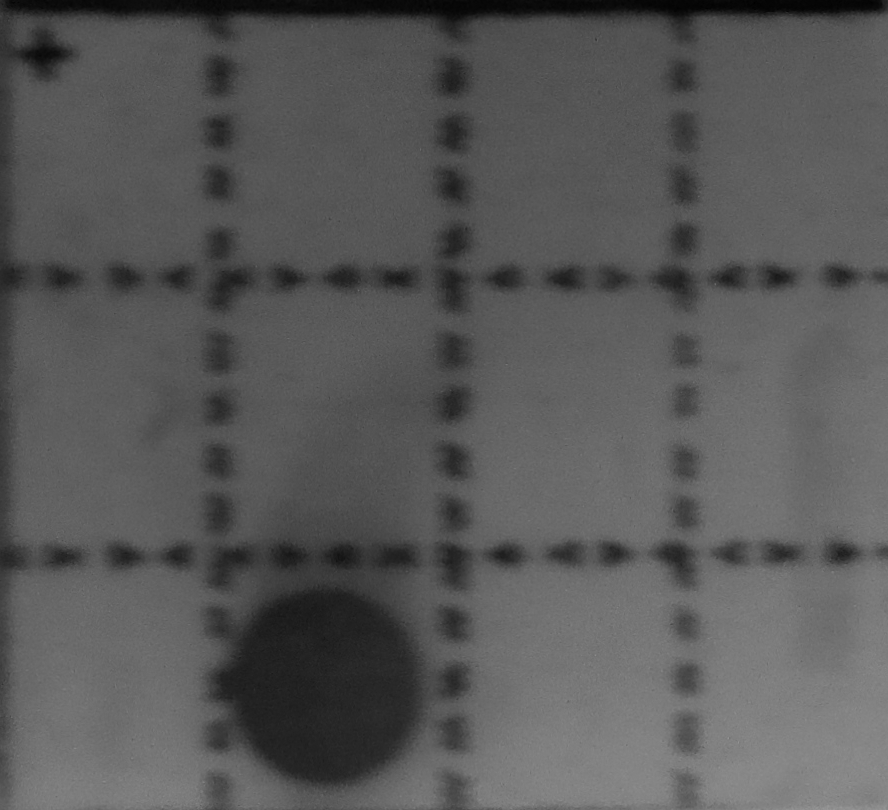

Supplement: Supplemental Information 1 [file peerj-11-15325-s001.zip › Raw Data/Results of 179 clinical samples of septicemia by membrane microarray (grayscale)-1/602492.tif]

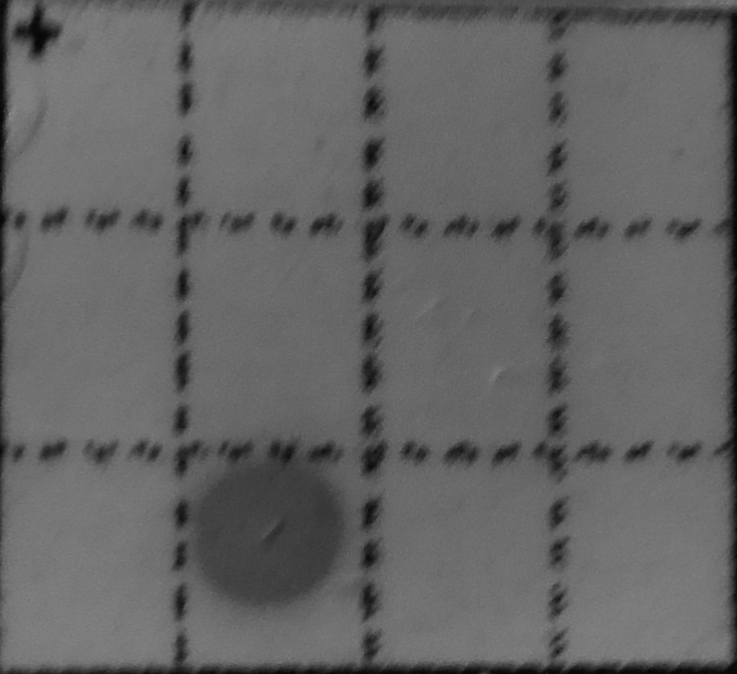

Supplement: Supplemental Information 1 [file peerj-11-15325-s001.zip › Raw Data/Results of 179 clinical samples of septicemia by membrane microarray (grayscale)-1/602549.tif]

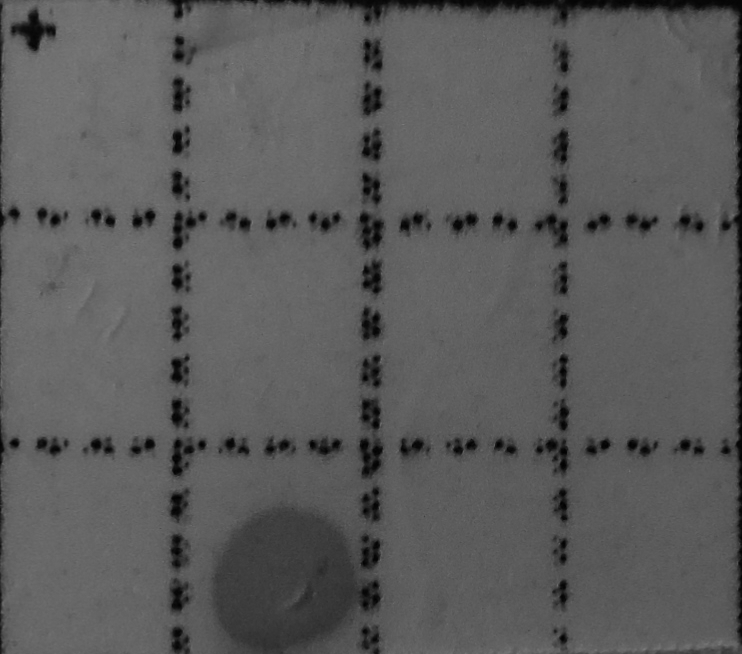

Supplement: Supplemental Information 1 [file peerj-11-15325-s001.zip › Raw Data/Results of 179 clinical samples of septicemia by membrane microarray (grayscale)-1/603977.tif]

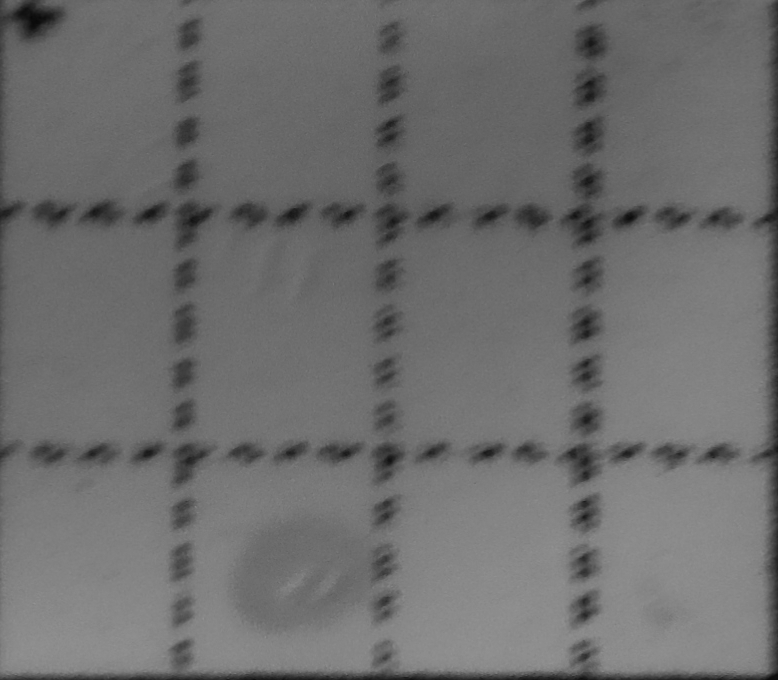

Supplement: Supplemental Information 1 [file peerj-11-15325-s001.zip › Raw Data/Results of 179 clinical samples of septicemia by membrane microarray (grayscale)-1/605643.tif]

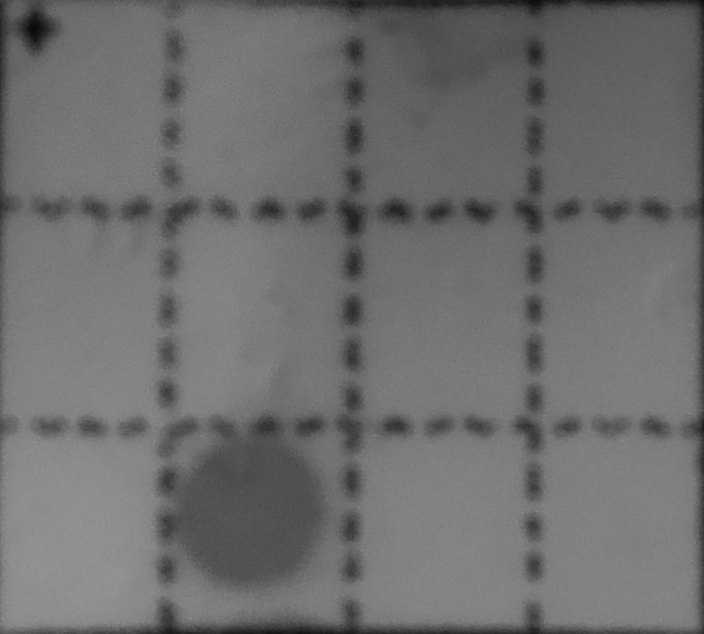

Supplement: Supplemental Information 1 [file peerj-11-15325-s001.zip › Raw Data/Results of 179 clinical samples of septicemia by membrane microarray (grayscale)-1/605742.tif]

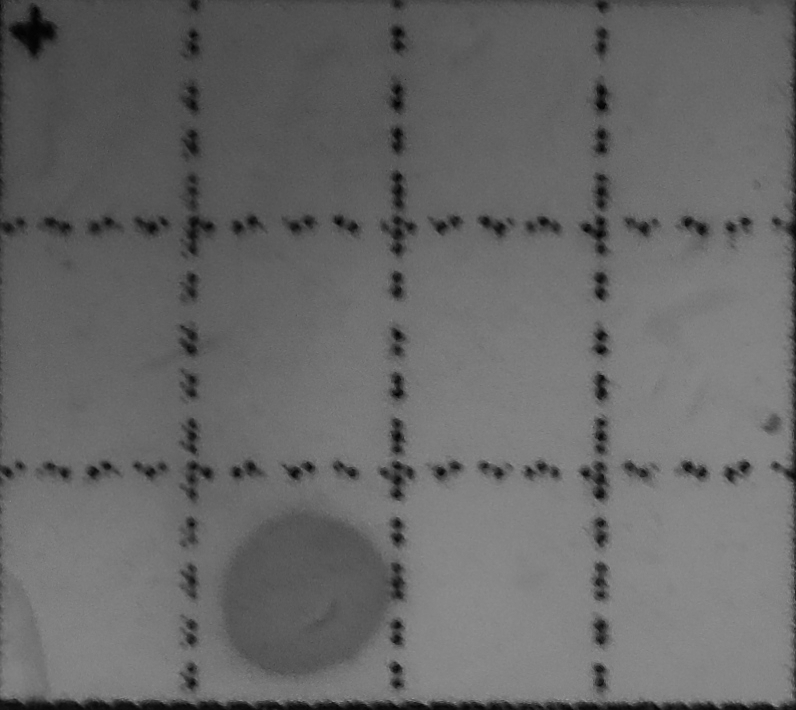

Supplement: Supplemental Information 1 [file peerj-11-15325-s001.zip › Raw Data/Results of 179 clinical samples of septicemia by membrane microarray (grayscale)-1/606486.tif]

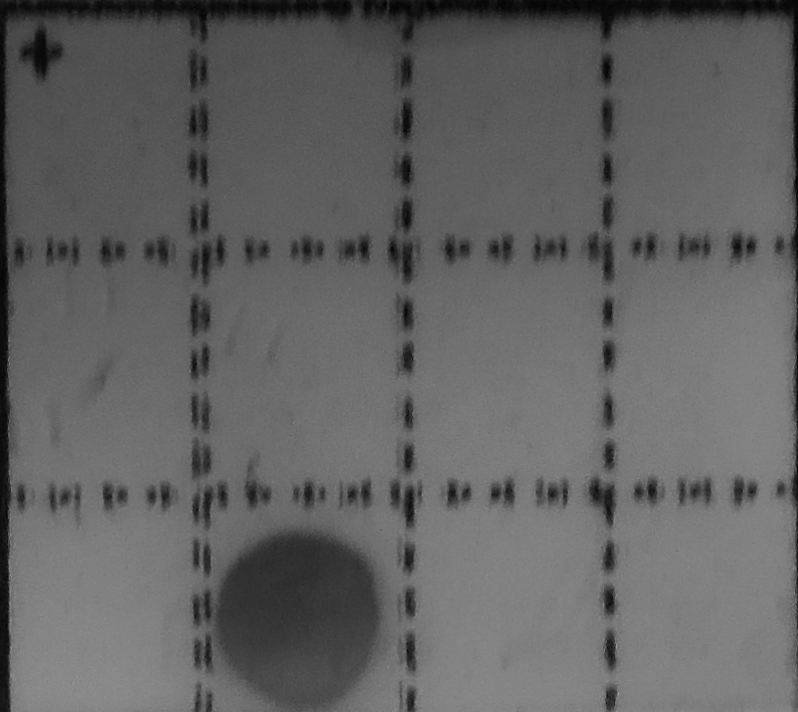

Supplement: Supplemental Information 1 [file peerj-11-15325-s001.zip › Raw Data/Results of 179 clinical samples of septicemia by membrane microarray (grayscale)-1/609230.tif]

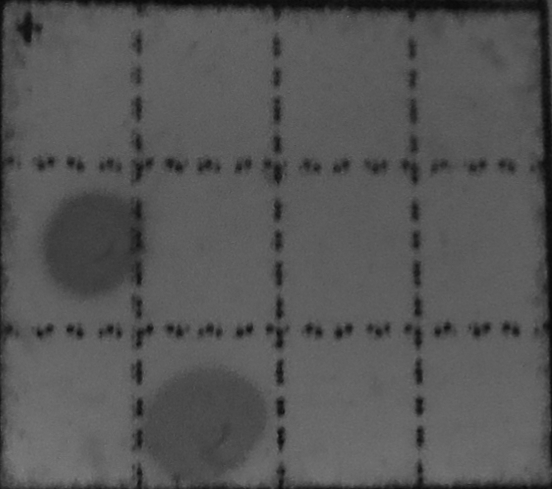

Supplement: Supplemental Information 1 [file peerj-11-15325-s001.zip › Raw Data/Results of 179 clinical samples of septicemia by membrane microarray (grayscale)-1/610806.tif]

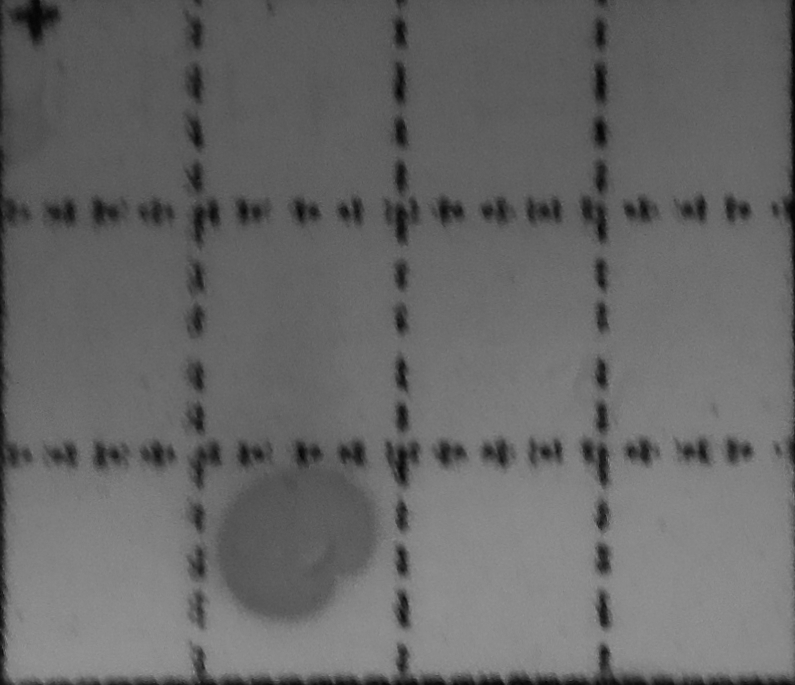

Supplement: Supplemental Information 1 [file peerj-11-15325-s001.zip › Raw Data/Results of 179 clinical samples of septicemia by membrane microarray (grayscale)-1/610860.tif]

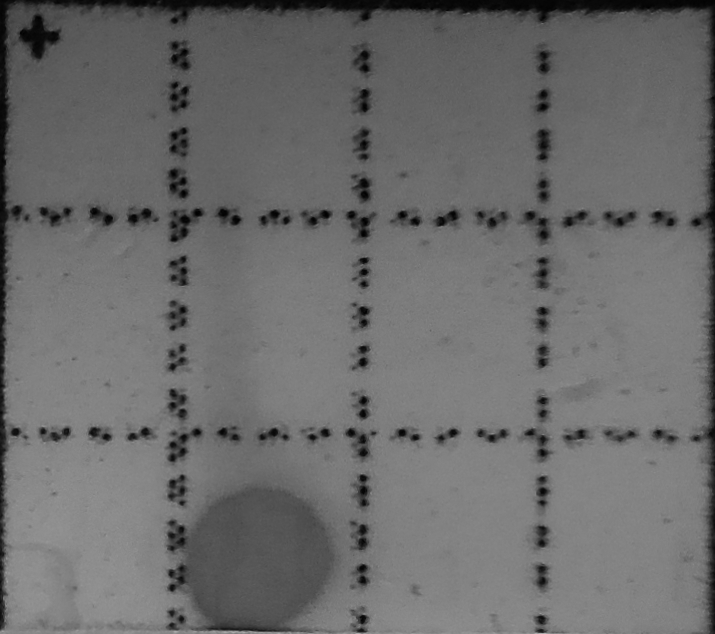

Supplement: Supplemental Information 1 [file peerj-11-15325-s001.zip › Raw Data/Results of 179 clinical samples of septicemia by membrane microarray (grayscale)-1/614012.tif]

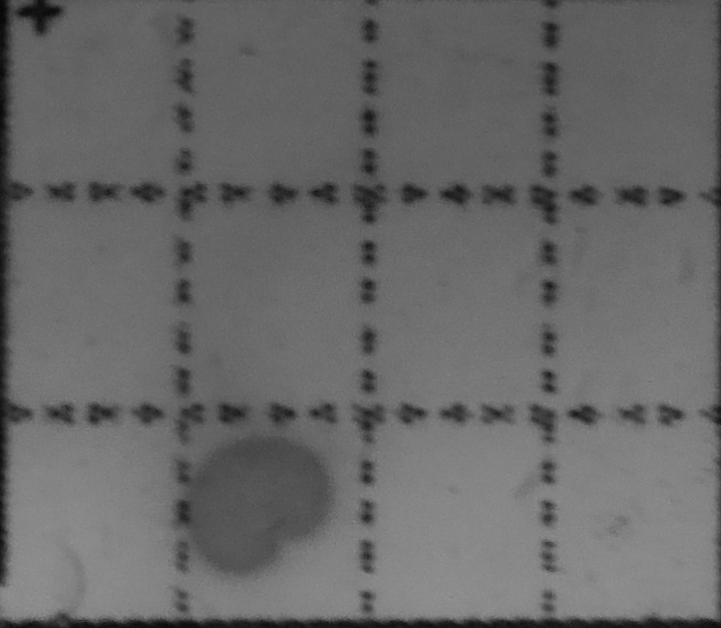

Supplement: Supplemental Information 1 [file peerj-11-15325-s001.zip › Raw Data/Results of 179 clinical samples of septicemia by membrane microarray (grayscale)-1/614116.tif]

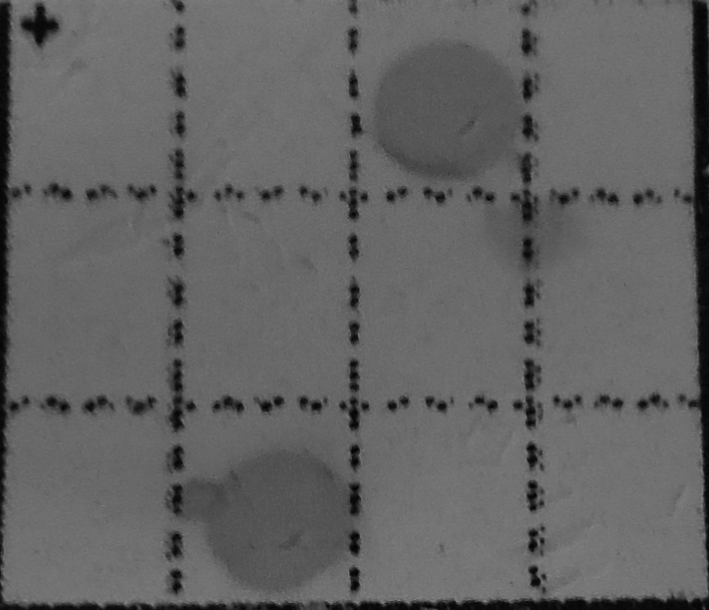

Supplement: Supplemental Information 2 [file peerj-11-15325-s002.zip › Raw Data-2/Results of 179 clinical samples of septicemia by membrane microarray (grayscale)-2/110783.tif]

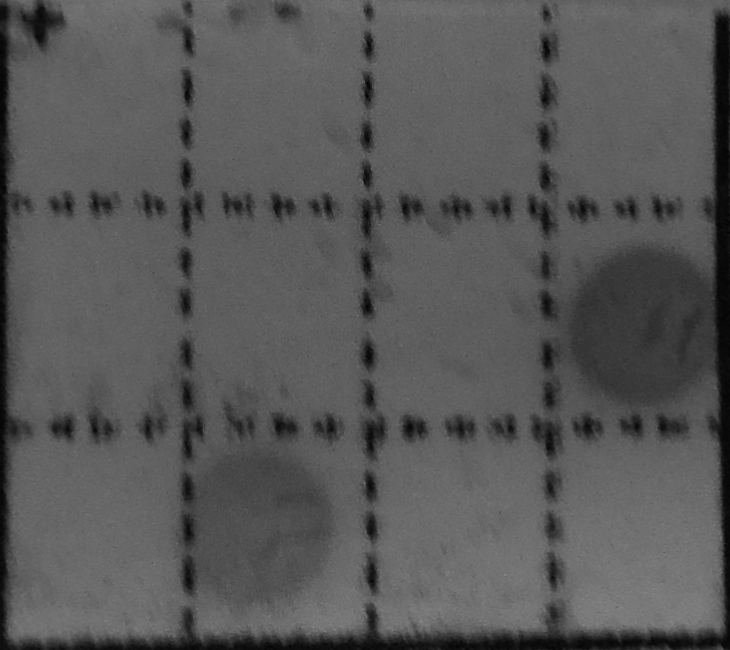

Supplement: Supplemental Information 2 [file peerj-11-15325-s002.zip › Raw Data-2/Results of 179 clinical samples of septicemia by membrane microarray (grayscale)-2/123776.tif]

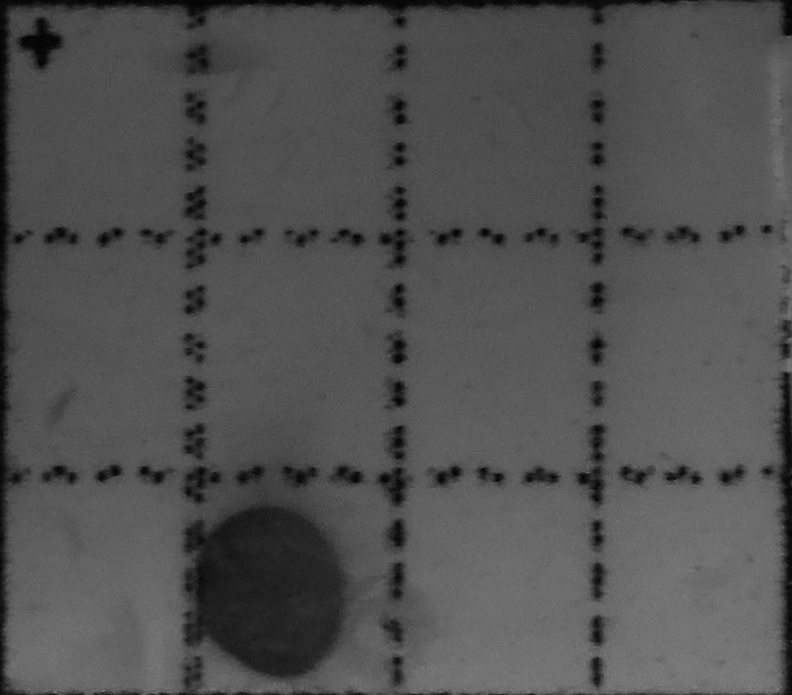

Supplement: Supplemental Information 2 [file peerj-11-15325-s002.zip › Raw Data-2/Results of 179 clinical samples of septicemia by membrane microarray (grayscale)-2/144431.tif]

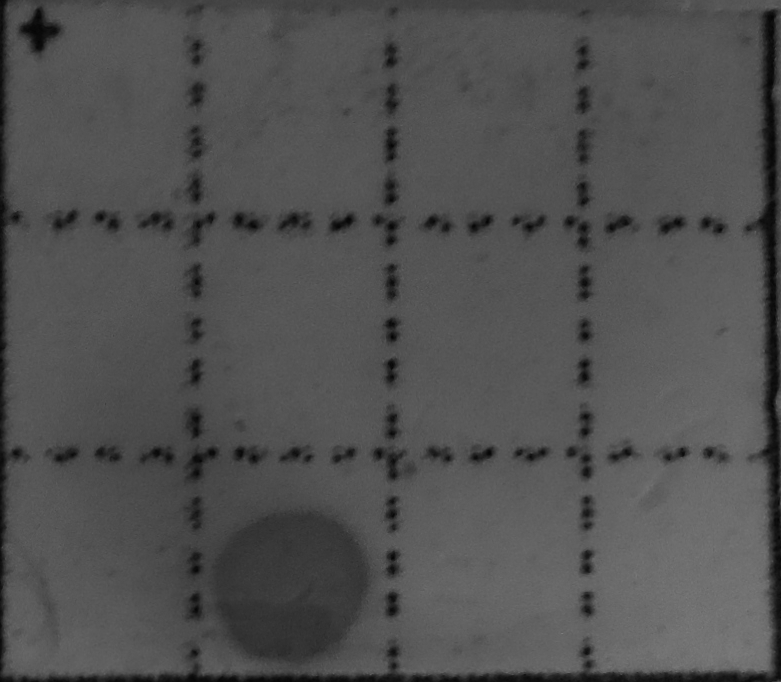

Supplement: Supplemental Information 2 [file peerj-11-15325-s002.zip › Raw Data-2/Results of 179 clinical samples of septicemia by membrane microarray (grayscale)-2/147881.tif]

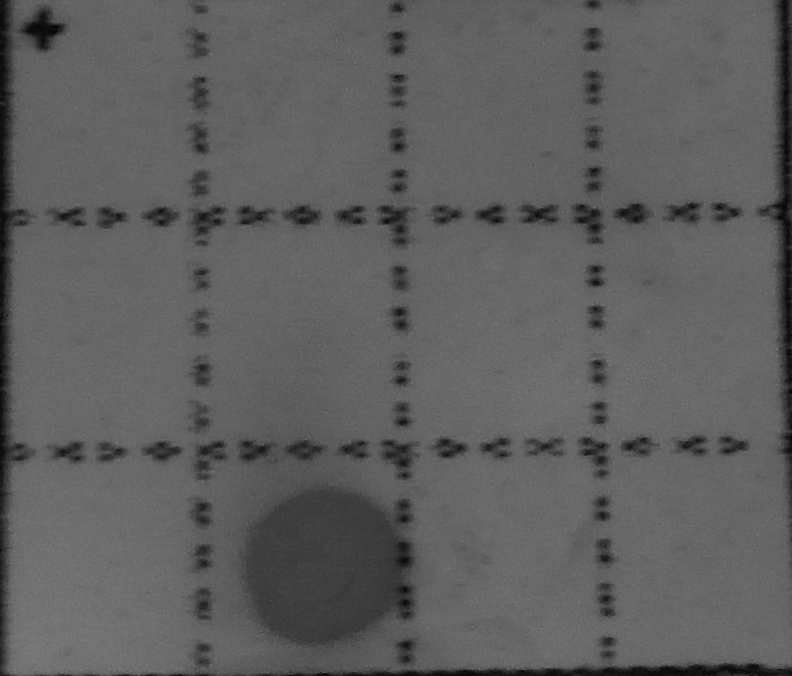

Supplement: Supplemental Information 2 [file peerj-11-15325-s002.zip › Raw Data-2/Results of 179 clinical samples of septicemia by membrane microarray (grayscale)-2/179447.tif]
