# Supplementary figures and images for: A new application of multiplex PCR combined with membrane biochip assay for rapid detection of 9 common pathogens in sepsis (part 2 of 2)
Source: PeerJ. 2023 May 12;11:e15325. doi: 10.7717/peerj.15325 (PMC10184654; doi:10.7717/peerj.15325)

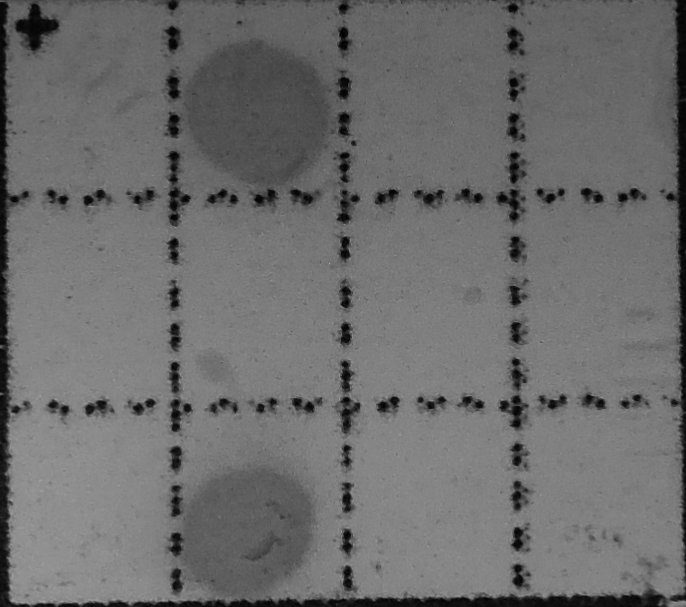

Supplement: Supplemental Information 2 [file peerj-11-15325-s002.zip › Raw Data-2/Results of 179 clinical samples of septicemia by membrane microarray (grayscale)-2/182996.tif]

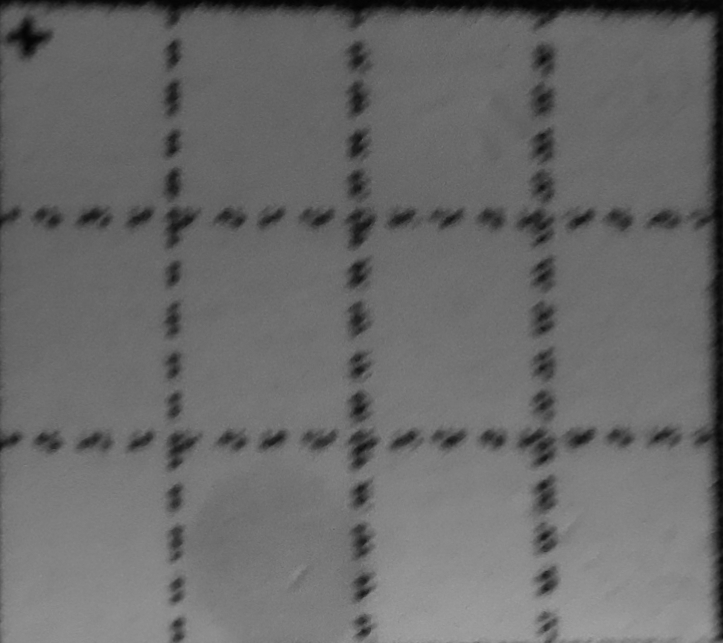

Supplement: Supplemental Information 2 [file peerj-11-15325-s002.zip › Raw Data-2/Results of 179 clinical samples of septicemia by membrane microarray (grayscale)-2/189211.tif]

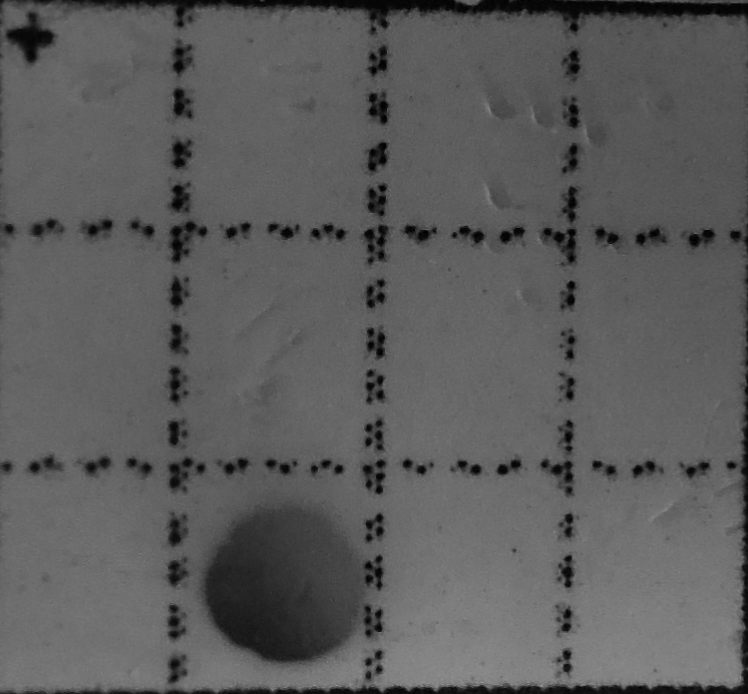

Supplement: Supplemental Information 2 [file peerj-11-15325-s002.zip › Raw Data-2/Results of 179 clinical samples of septicemia by membrane microarray (grayscale)-2/203840.tif]

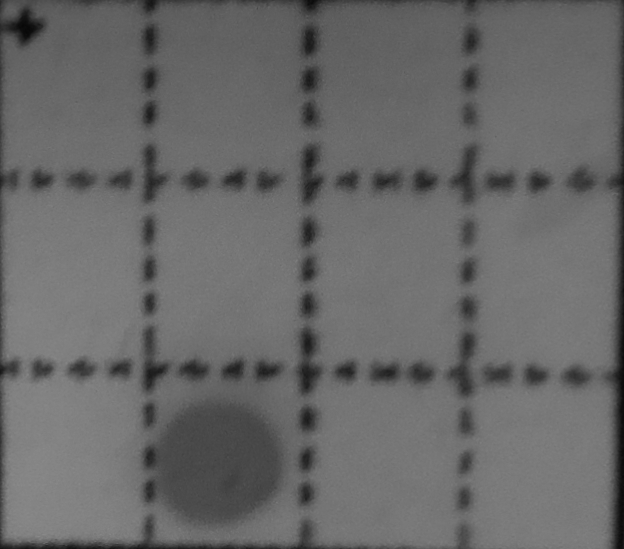

Supplement: Supplemental Information 2 [file peerj-11-15325-s002.zip › Raw Data-2/Results of 179 clinical samples of septicemia by membrane microarray (grayscale)-2/206018.tif]

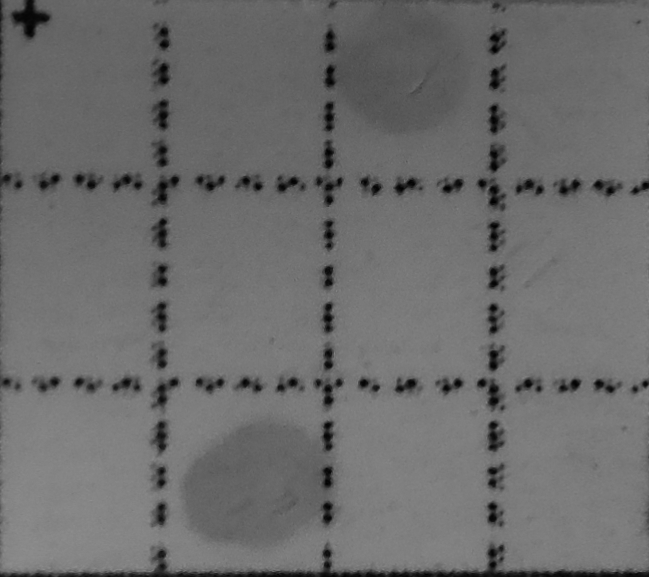

Supplement: Supplemental Information 2 [file peerj-11-15325-s002.zip › Raw Data-2/Results of 179 clinical samples of septicemia by membrane microarray (grayscale)-2/219946.tif]

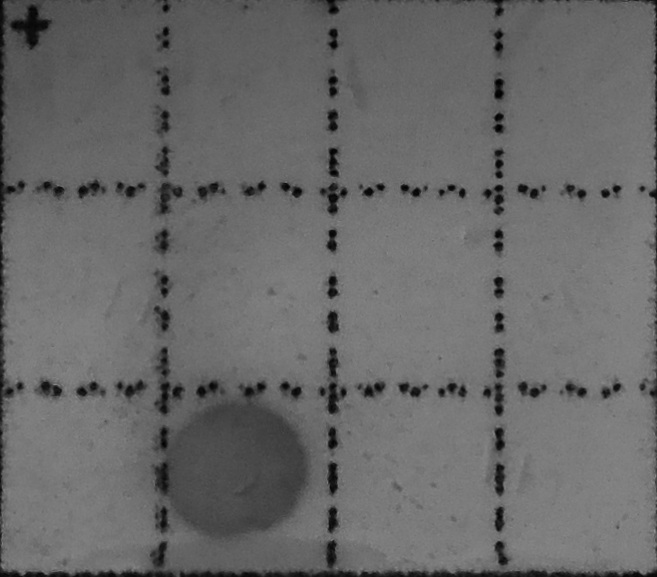

Supplement: Supplemental Information 2 [file peerj-11-15325-s002.zip › Raw Data-2/Results of 179 clinical samples of septicemia by membrane microarray (grayscale)-2/223431.tif]

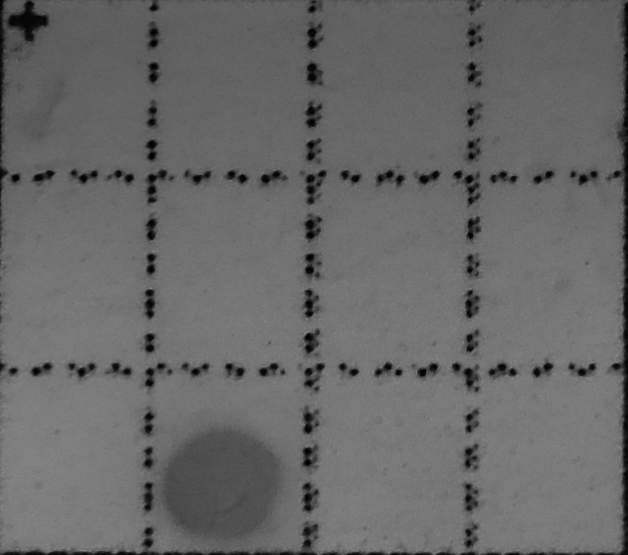

Supplement: Supplemental Information 2 [file peerj-11-15325-s002.zip › Raw Data-2/Results of 179 clinical samples of septicemia by membrane microarray (grayscale)-2/230871.tif]

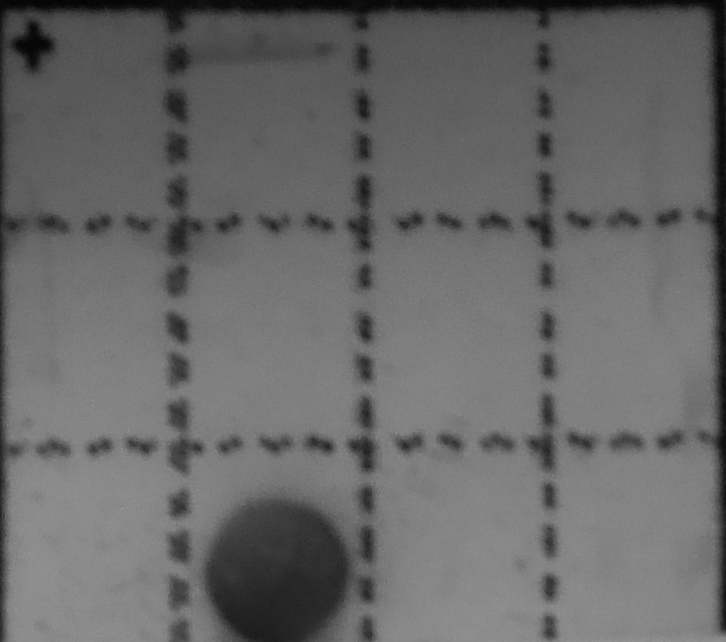

Supplement: Supplemental Information 2 [file peerj-11-15325-s002.zip › Raw Data-2/Results of 179 clinical samples of septicemia by membrane microarray (grayscale)-2/236368.tif]

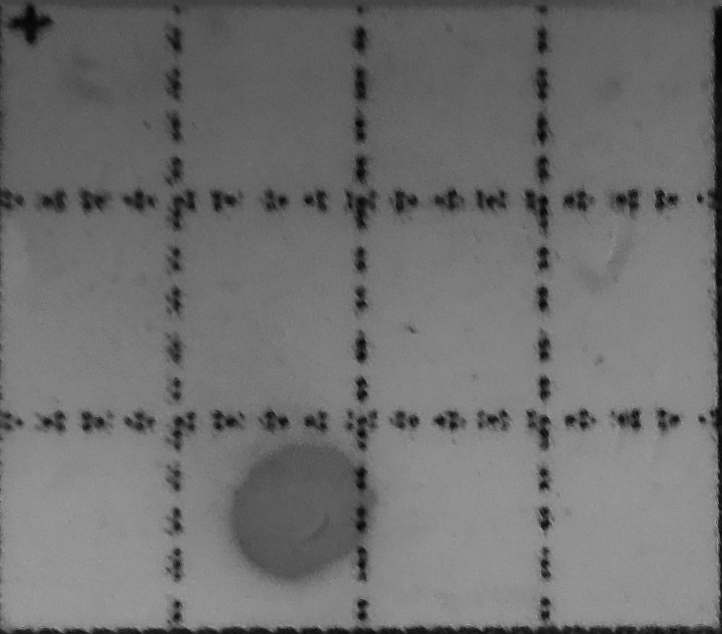

Supplement: Supplemental Information 2 [file peerj-11-15325-s002.zip › Raw Data-2/Results of 179 clinical samples of septicemia by membrane microarray (grayscale)-2/241717.tif]

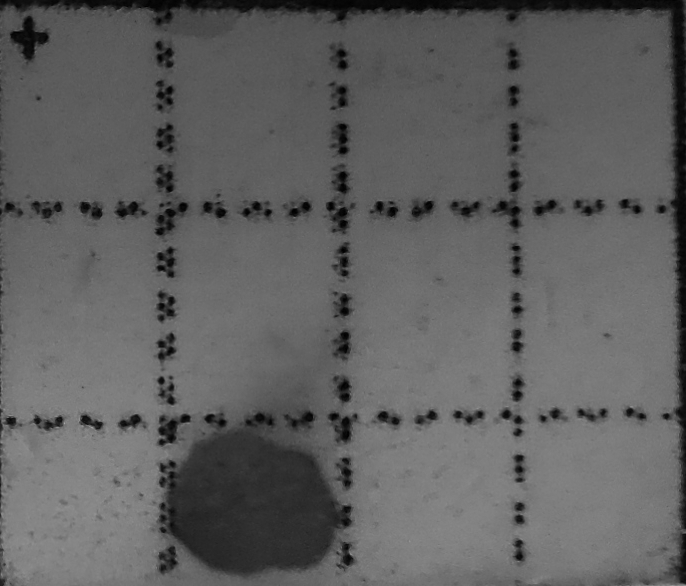

Supplement: Supplemental Information 2 [file peerj-11-15325-s002.zip › Raw Data-2/Results of 179 clinical samples of septicemia by membrane microarray (grayscale)-2/243424.tif]

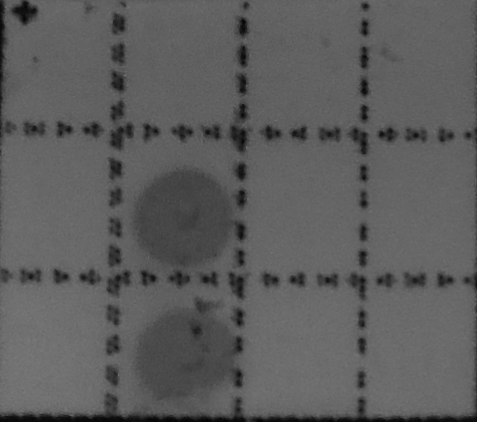

Supplement: Supplemental Information 2 [file peerj-11-15325-s002.zip › Raw Data-2/Results of 179 clinical samples of septicemia by membrane microarray (grayscale)-2/253525.tif]

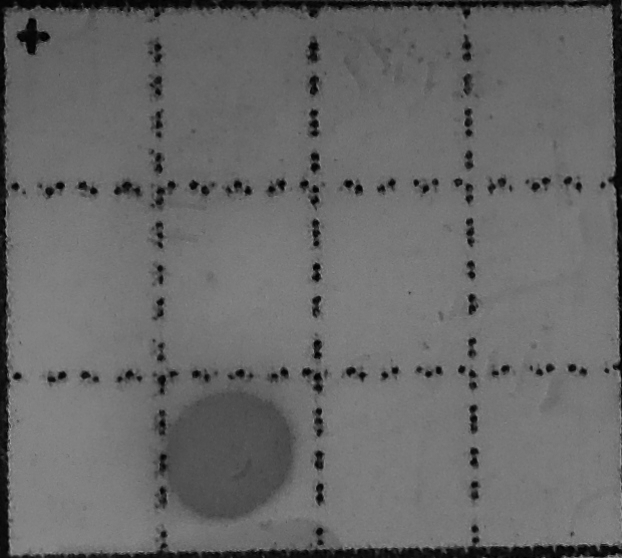

Supplement: Supplemental Information 2 [file peerj-11-15325-s002.zip › Raw Data-2/Results of 179 clinical samples of septicemia by membrane microarray (grayscale)-2/259639.tif]

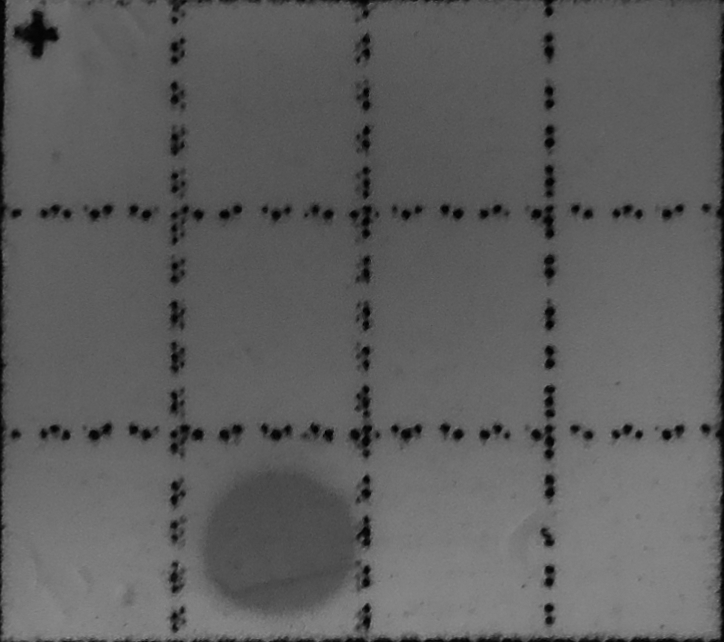

Supplement: Supplemental Information 2 [file peerj-11-15325-s002.zip › Raw Data-2/Results of 179 clinical samples of septicemia by membrane microarray (grayscale)-2/266648.tif]

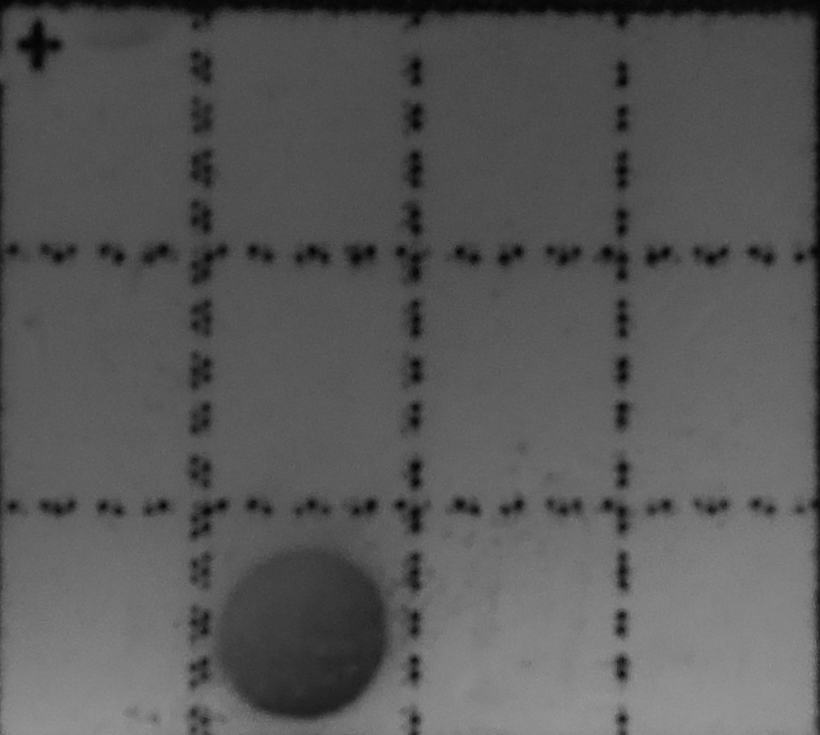

Supplement: Supplemental Information 2 [file peerj-11-15325-s002.zip › Raw Data-2/Results of 179 clinical samples of septicemia by membrane microarray (grayscale)-2/267052.tif]

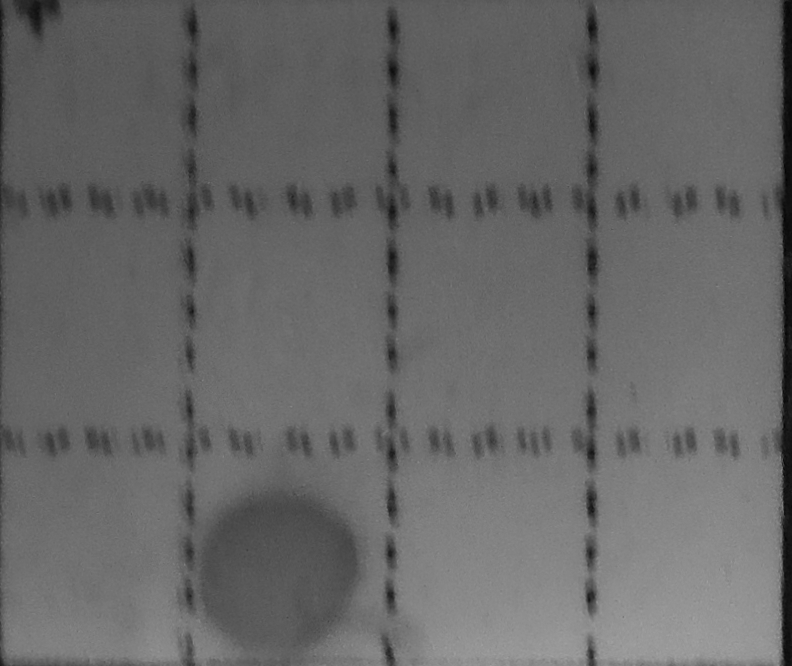

Supplement: Supplemental Information 2 [file peerj-11-15325-s002.zip › Raw Data-2/Results of 179 clinical samples of septicemia by membrane microarray (grayscale)-2/269125.tif]

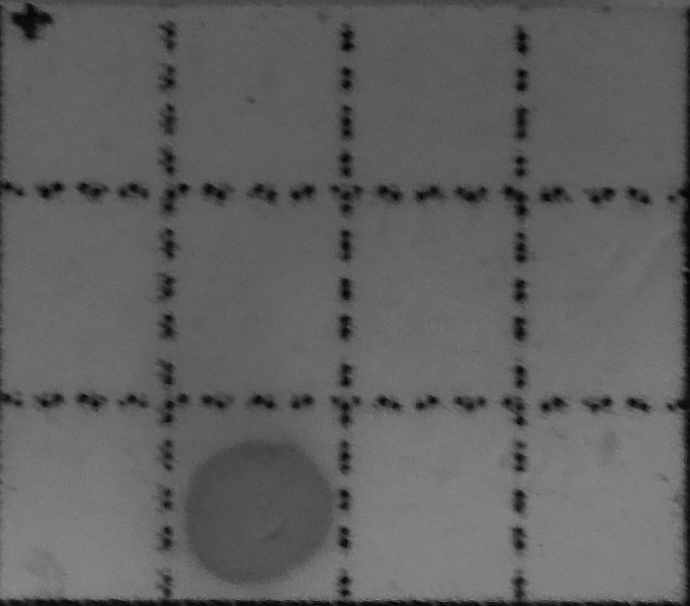

Supplement: Supplemental Information 2 [file peerj-11-15325-s002.zip › Raw Data-2/Results of 179 clinical samples of septicemia by membrane microarray (grayscale)-2/269726.tif]

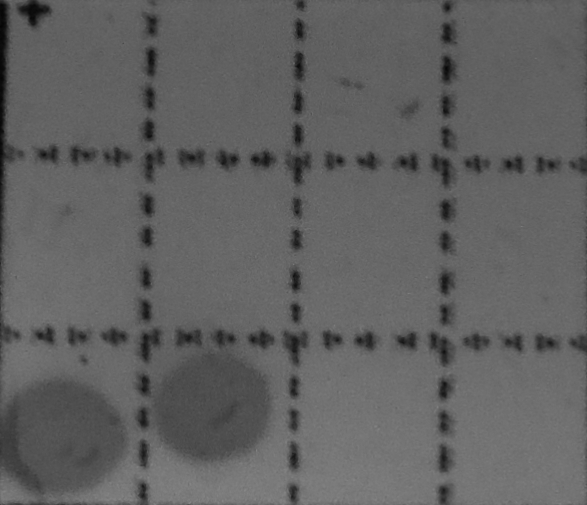

Supplement: Supplemental Information 2 [file peerj-11-15325-s002.zip › Raw Data-2/Results of 179 clinical samples of septicemia by membrane microarray (grayscale)-2/273990.tif]

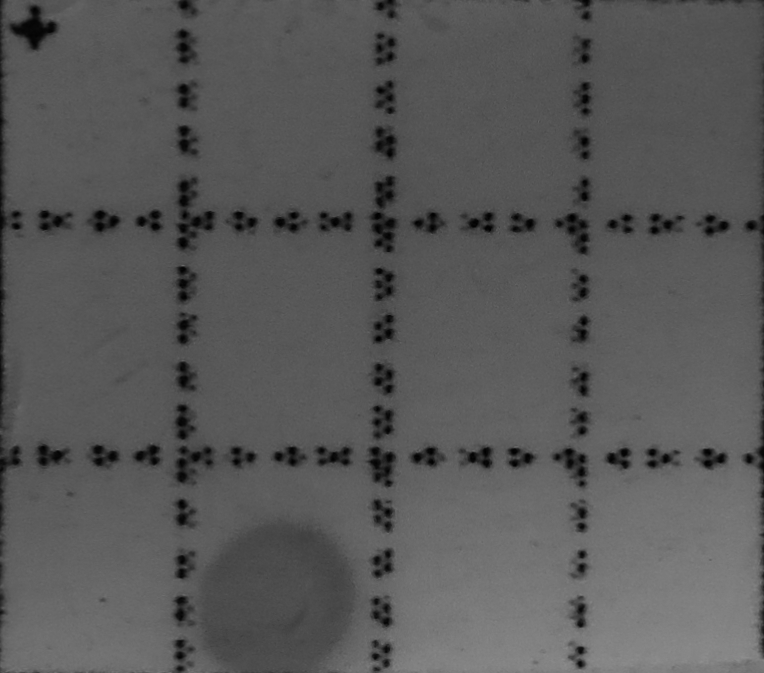

Supplement: Supplemental Information 2 [file peerj-11-15325-s002.zip › Raw Data-2/Results of 179 clinical samples of septicemia by membrane microarray (grayscale)-2/295480.tif]

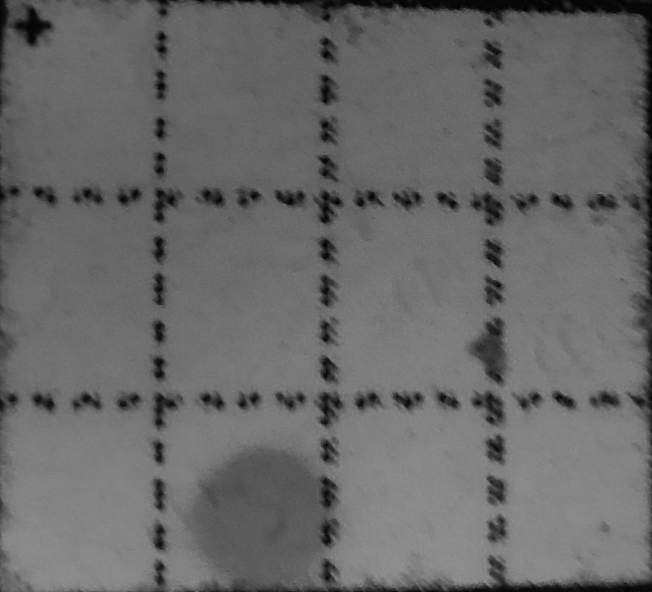

Supplement: Supplemental Information 2 [file peerj-11-15325-s002.zip › Raw Data-2/Results of 179 clinical samples of septicemia by membrane microarray (grayscale)-2/304683.tif]

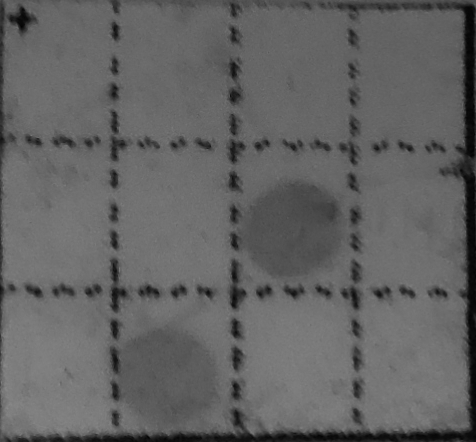

Supplement: Supplemental Information 2 [file peerj-11-15325-s002.zip › Raw Data-2/Results of 179 clinical samples of septicemia by membrane microarray (grayscale)-2/310183.tif]

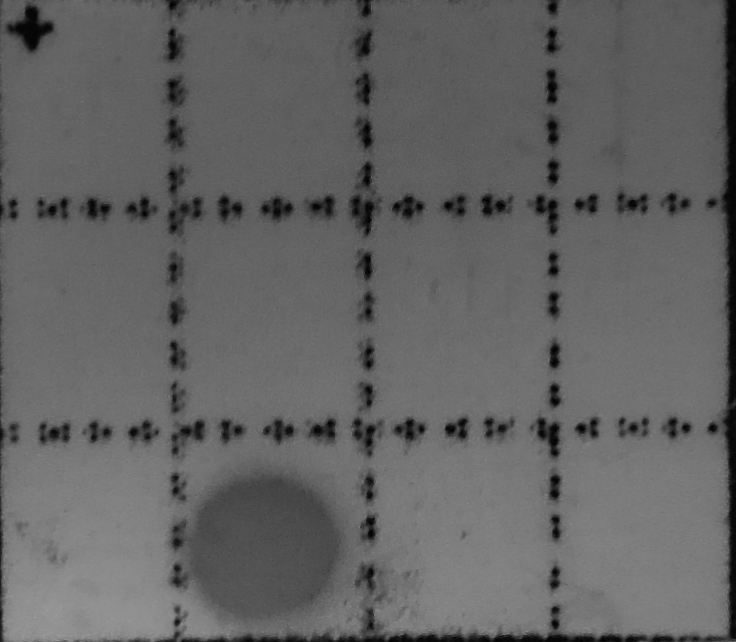

Supplement: Supplemental Information 2 [file peerj-11-15325-s002.zip › Raw Data-2/Results of 179 clinical samples of septicemia by membrane microarray (grayscale)-2/323230.tif]

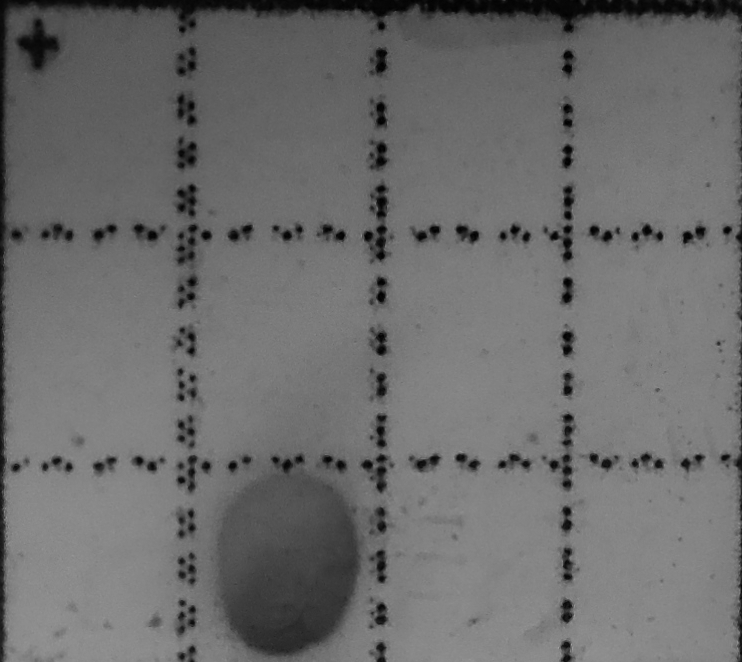

Supplement: Supplemental Information 2 [file peerj-11-15325-s002.zip › Raw Data-2/Results of 179 clinical samples of septicemia by membrane microarray (grayscale)-2/329486.tif]

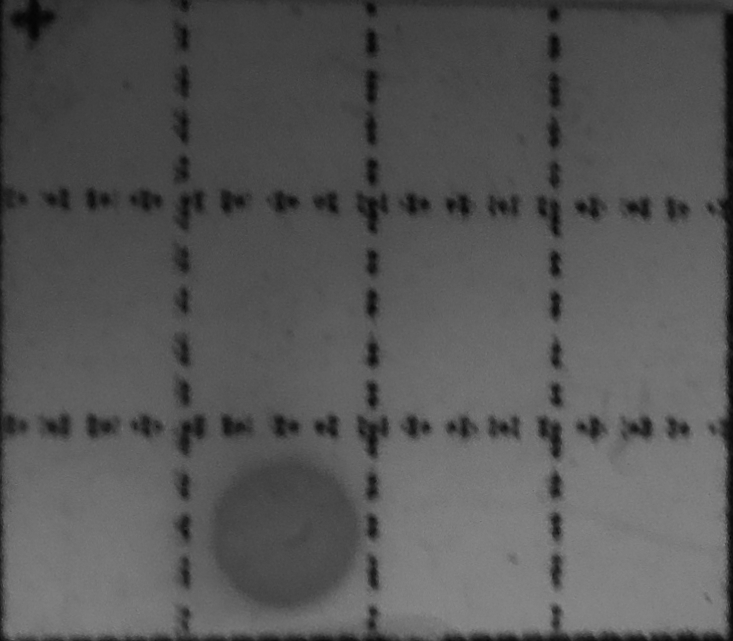

Supplement: Supplemental Information 2 [file peerj-11-15325-s002.zip › Raw Data-2/Results of 179 clinical samples of septicemia by membrane microarray (grayscale)-2/329631.tif]

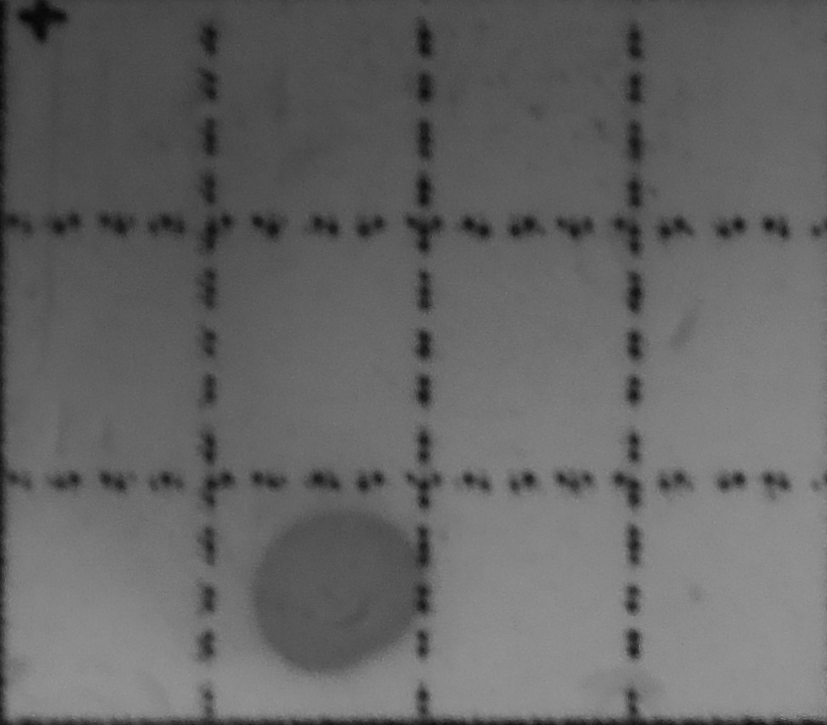

Supplement: Supplemental Information 2 [file peerj-11-15325-s002.zip › Raw Data-2/Results of 179 clinical samples of septicemia by membrane microarray (grayscale)-2/341598.tif]

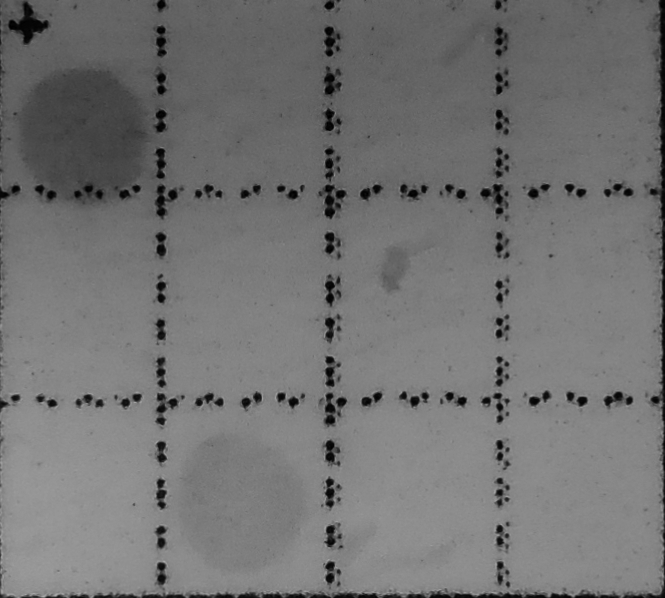

Supplement: Supplemental Information 2 [file peerj-11-15325-s002.zip › Raw Data-2/Results of 179 clinical samples of septicemia by membrane microarray (grayscale)-2/363214.tif]

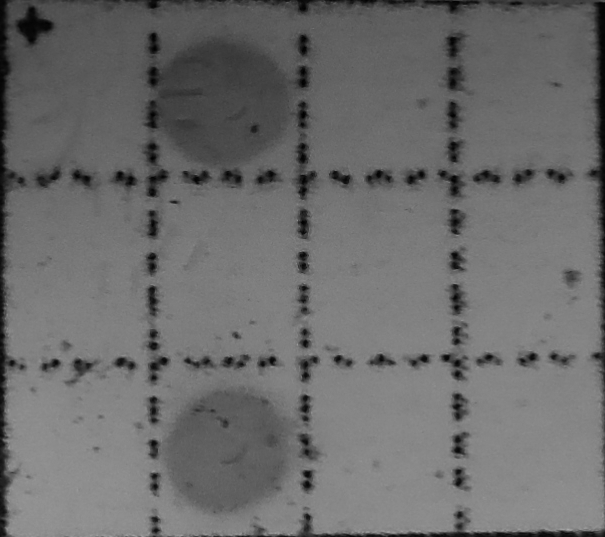

Supplement: Supplemental Information 2 [file peerj-11-15325-s002.zip › Raw Data-2/Results of 179 clinical samples of septicemia by membrane microarray (grayscale)-2/369411.tif]

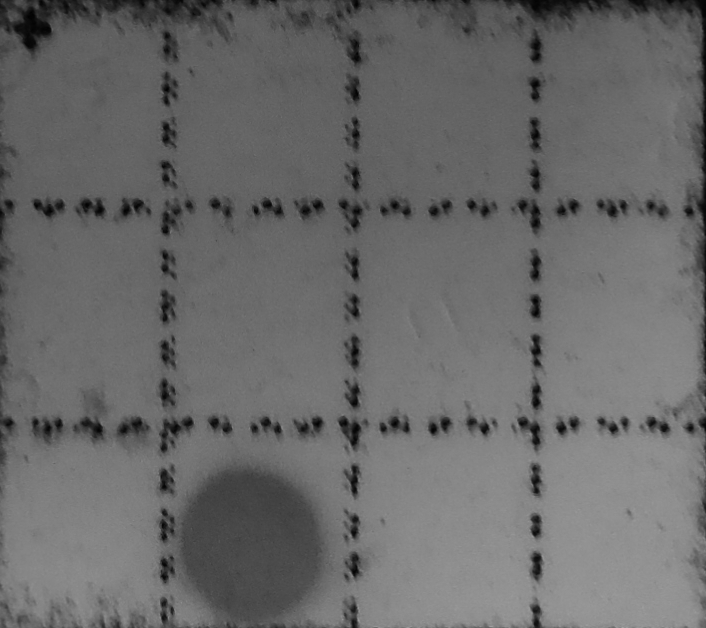

Supplement: Supplemental Information 2 [file peerj-11-15325-s002.zip › Raw Data-2/Results of 179 clinical samples of septicemia by membrane microarray (grayscale)-2/384846.tif]

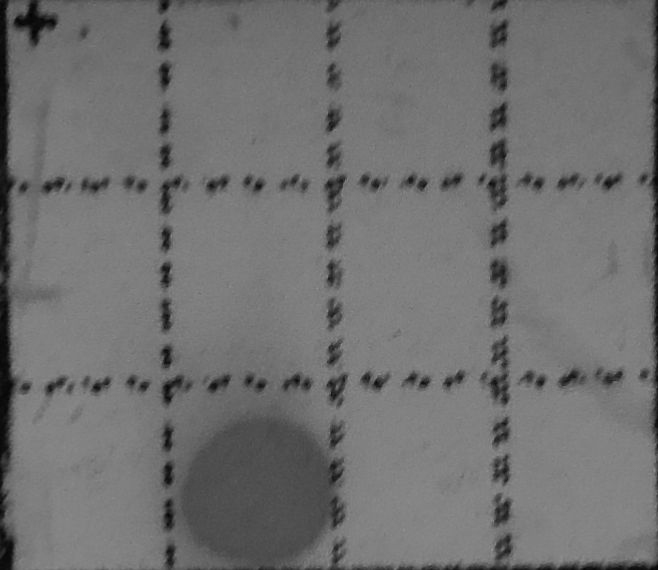

Supplement: Supplemental Information 2 [file peerj-11-15325-s002.zip › Raw Data-2/Results of 179 clinical samples of septicemia by membrane microarray (grayscale)-2/387525.tif]

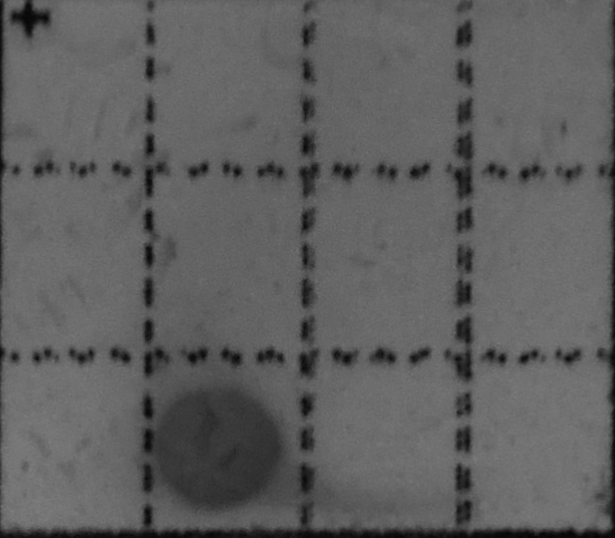

Supplement: Supplemental Information 2 [file peerj-11-15325-s002.zip › Raw Data-2/Results of 179 clinical samples of septicemia by membrane microarray (grayscale)-2/396874.tif]

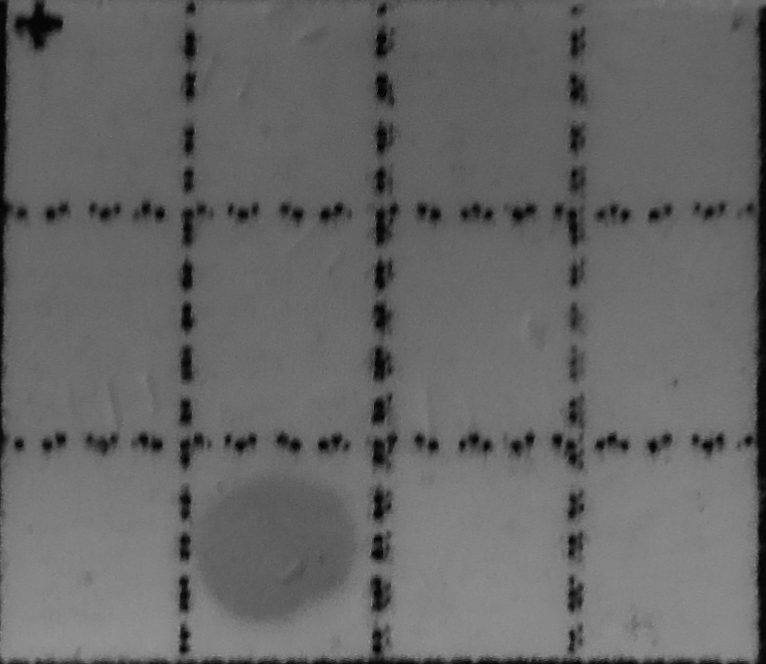

Supplement: Supplemental Information 2 [file peerj-11-15325-s002.zip › Raw Data-2/Results of 179 clinical samples of septicemia by membrane microarray (grayscale)-2/400747.tif]

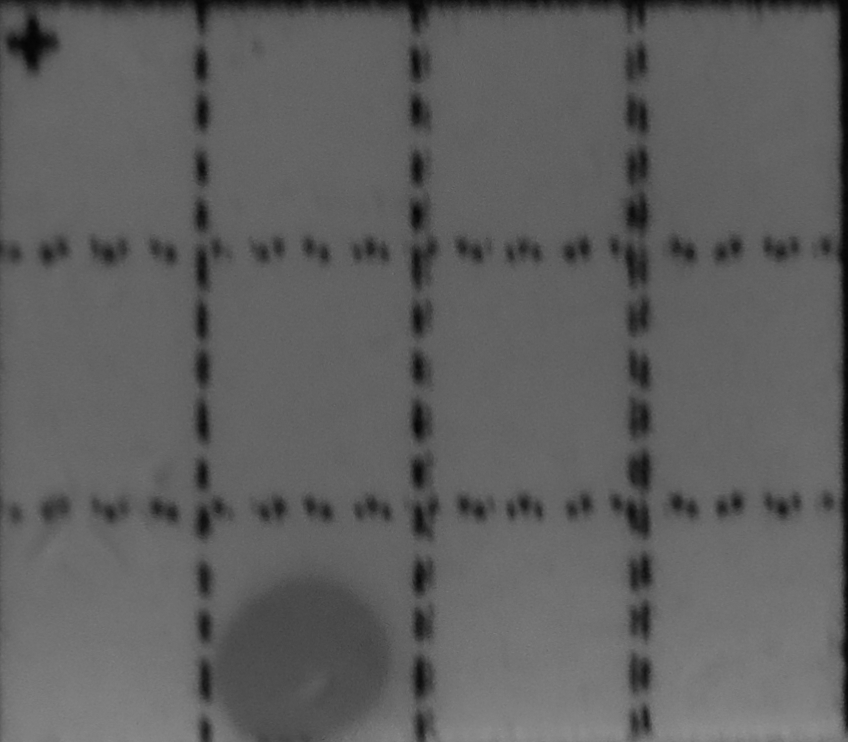

Supplement: Supplemental Information 2 [file peerj-11-15325-s002.zip › Raw Data-2/Results of 179 clinical samples of septicemia by membrane microarray (grayscale)-2/426680.tif]

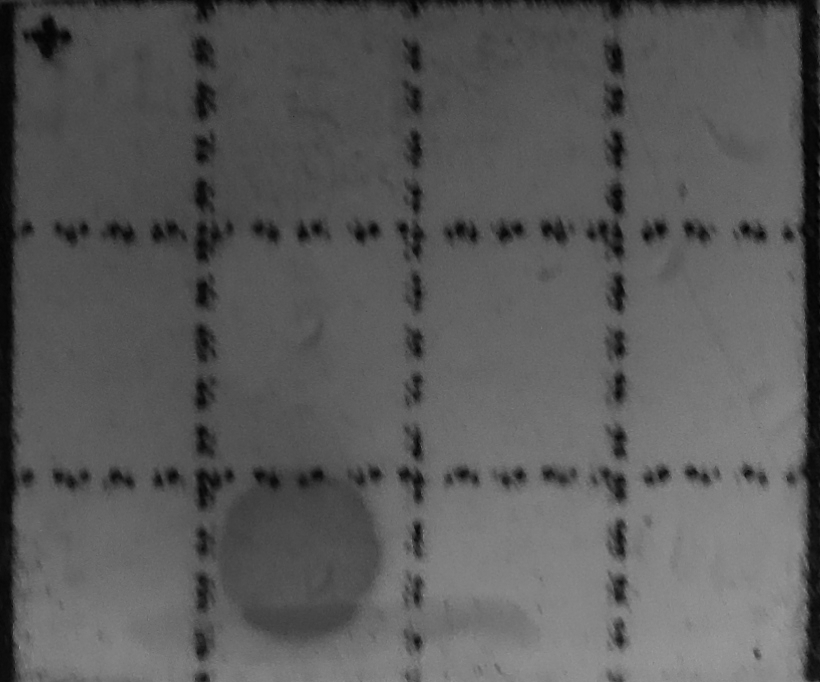

Supplement: Supplemental Information 2 [file peerj-11-15325-s002.zip › Raw Data-2/Results of 179 clinical samples of septicemia by membrane microarray (grayscale)-2/434635.tif]

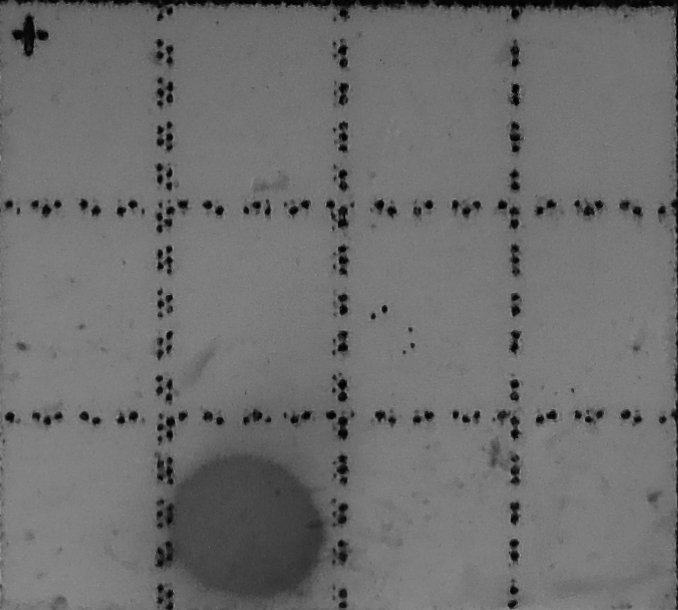

Supplement: Supplemental Information 2 [file peerj-11-15325-s002.zip › Raw Data-2/Results of 179 clinical samples of septicemia by membrane microarray (grayscale)-2/436281.tif]

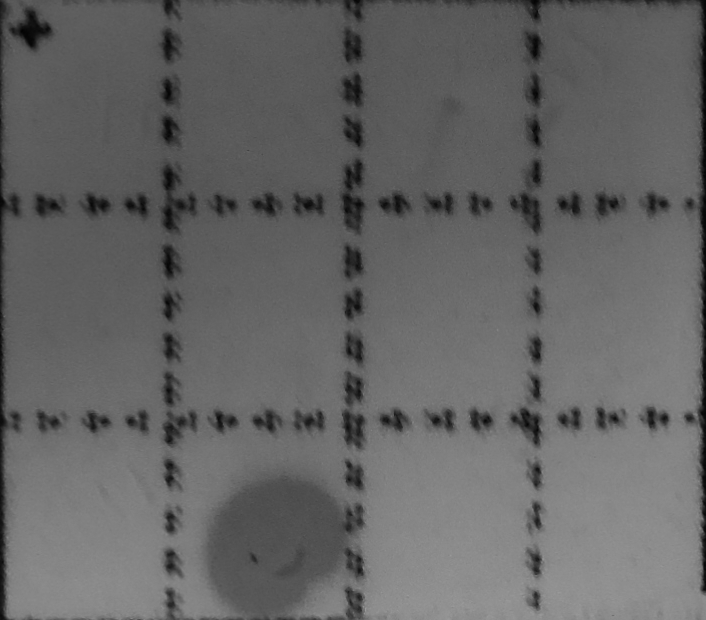

Supplement: Supplemental Information 2 [file peerj-11-15325-s002.zip › Raw Data-2/Results of 179 clinical samples of septicemia by membrane microarray (grayscale)-2/448992.tif]

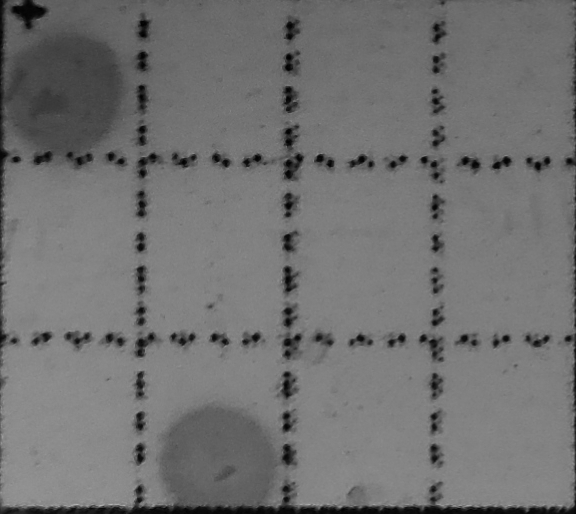

Supplement: Supplemental Information 2 [file peerj-11-15325-s002.zip › Raw Data-2/Results of 179 clinical samples of septicemia by membrane microarray (grayscale)-2/458603.tif]

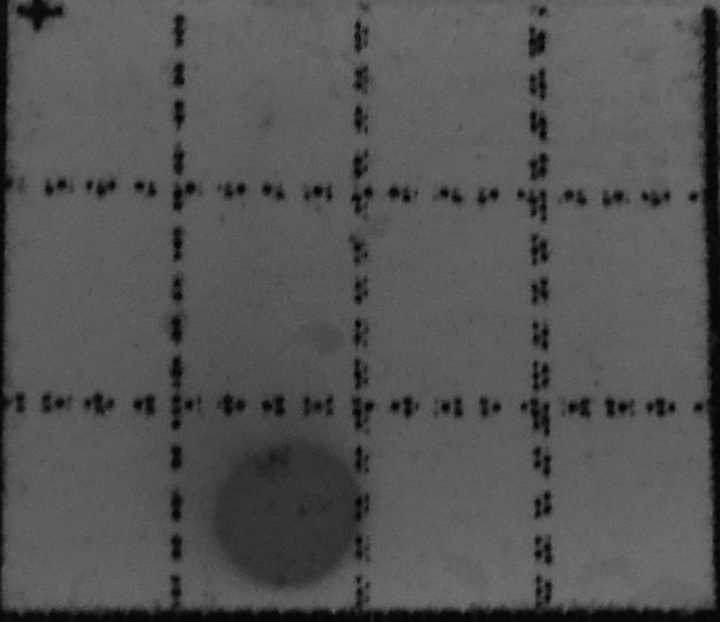

Supplement: Supplemental Information 2 [file peerj-11-15325-s002.zip › Raw Data-2/Results of 179 clinical samples of septicemia by membrane microarray (grayscale)-2/474526.tif]

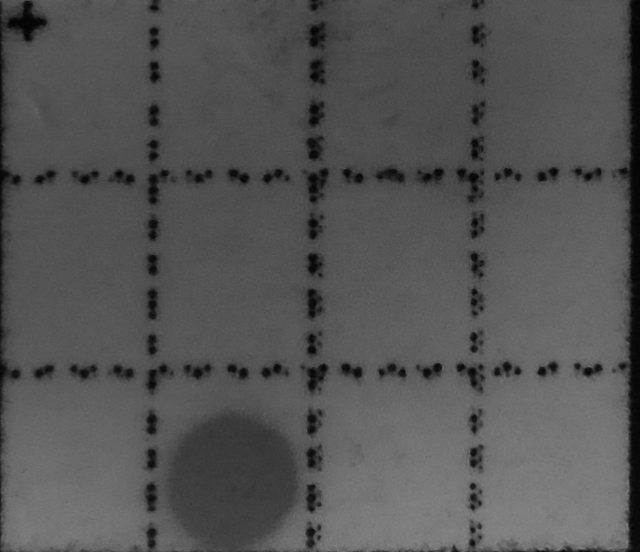

Supplement: Supplemental Information 2 [file peerj-11-15325-s002.zip › Raw Data-2/Results of 179 clinical samples of septicemia by membrane microarray (grayscale)-2/474540.tif]

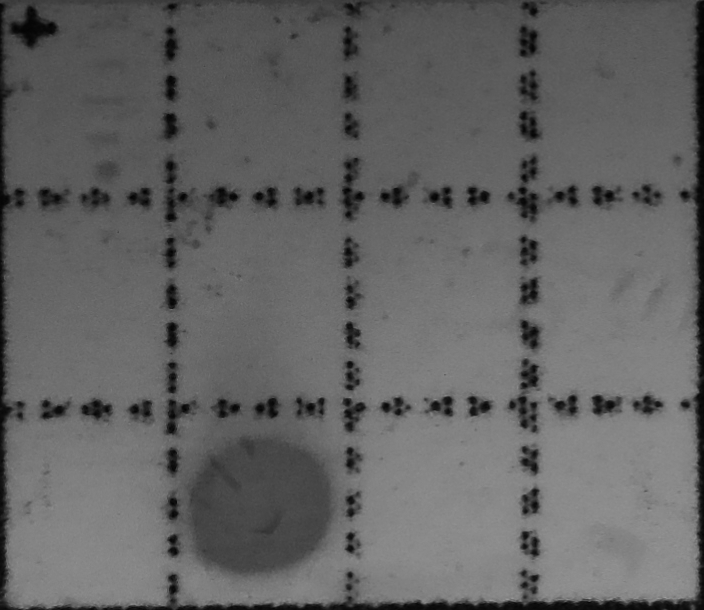

Supplement: Supplemental Information 2 [file peerj-11-15325-s002.zip › Raw Data-2/Results of 179 clinical samples of septicemia by membrane microarray (grayscale)-2/476184.tif]

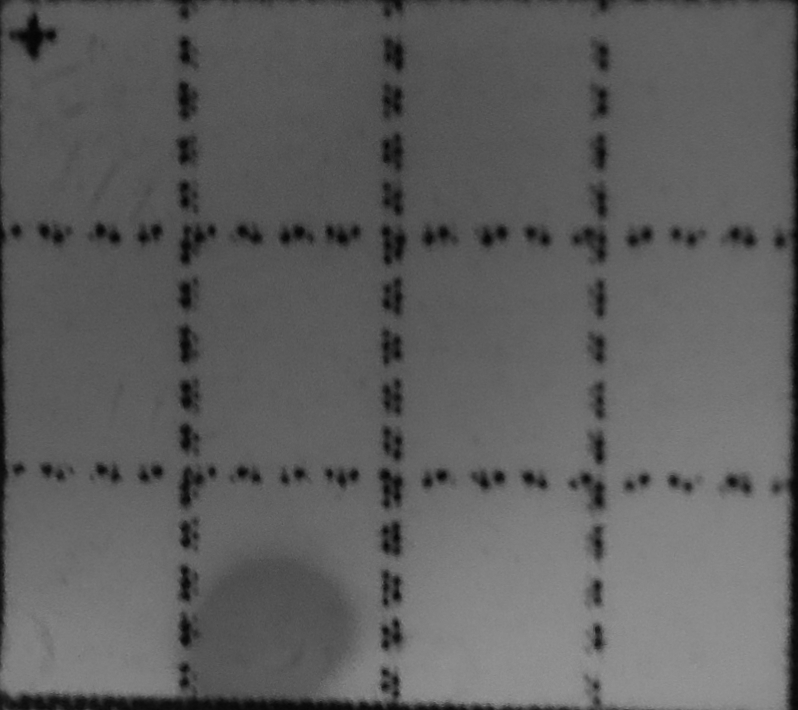

Supplement: Supplemental Information 2 [file peerj-11-15325-s002.zip › Raw Data-2/Results of 179 clinical samples of septicemia by membrane microarray (grayscale)-2/483988.tif]

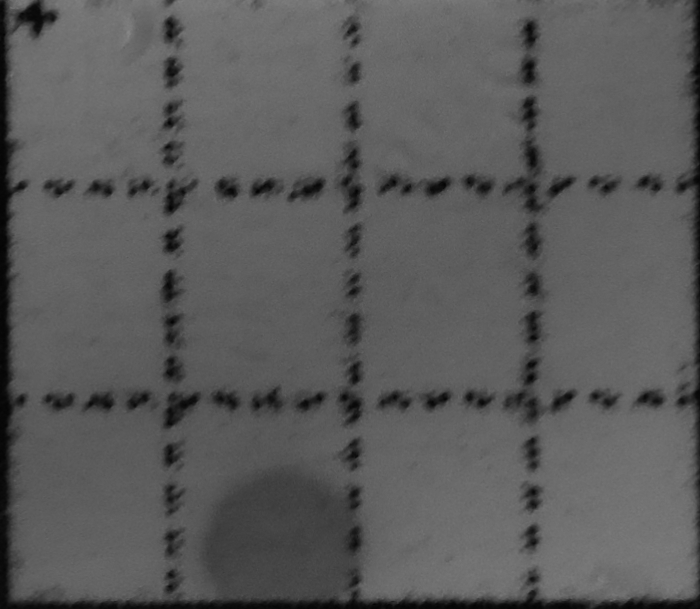

Supplement: Supplemental Information 2 [file peerj-11-15325-s002.zip › Raw Data-2/Results of 179 clinical samples of septicemia by membrane microarray (grayscale)-2/484698.tif]

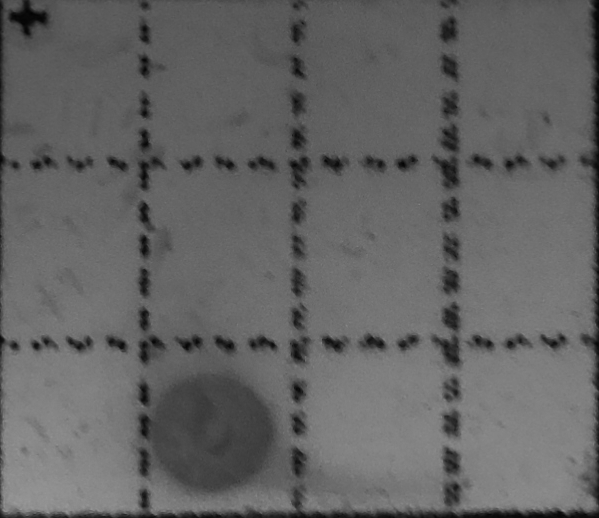

Supplement: Supplemental Information 2 [file peerj-11-15325-s002.zip › Raw Data-2/Results of 179 clinical samples of septicemia by membrane microarray (grayscale)-2/484998.tif]

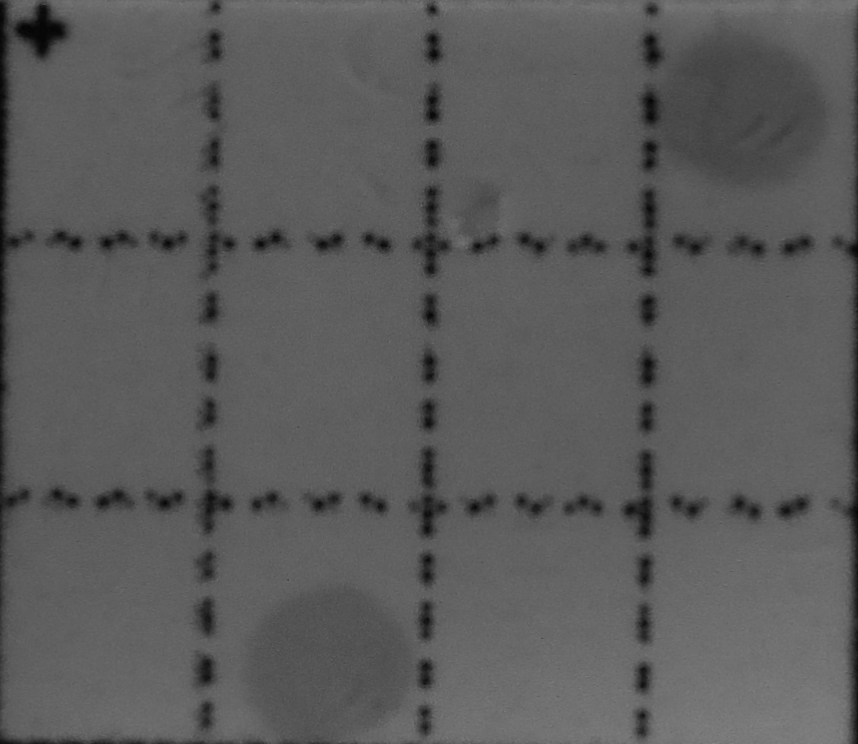

Supplement: Supplemental Information 2 [file peerj-11-15325-s002.zip › Raw Data-2/Results of 179 clinical samples of septicemia by membrane microarray (grayscale)-2/487493.tif]

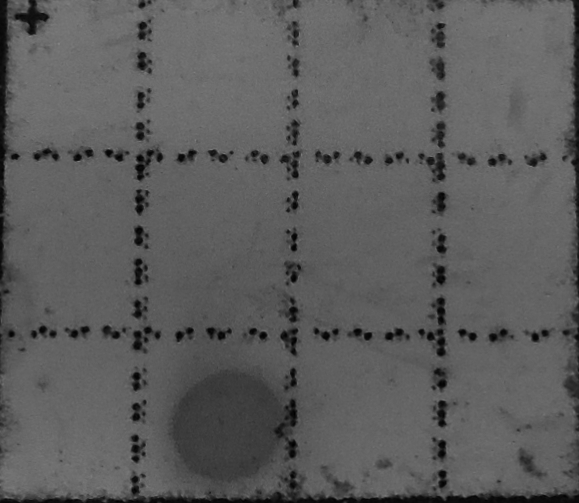

Supplement: Supplemental Information 2 [file peerj-11-15325-s002.zip › Raw Data-2/Results of 179 clinical samples of septicemia by membrane microarray (grayscale)-2/506348.tif]

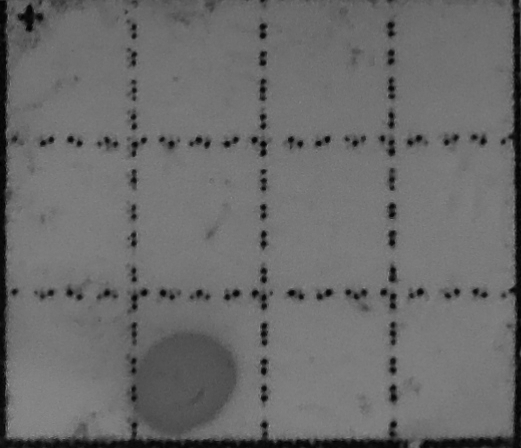

Supplement: Supplemental Information 2 [file peerj-11-15325-s002.zip › Raw Data-2/Results of 179 clinical samples of septicemia by membrane microarray (grayscale)-2/506608.tif]

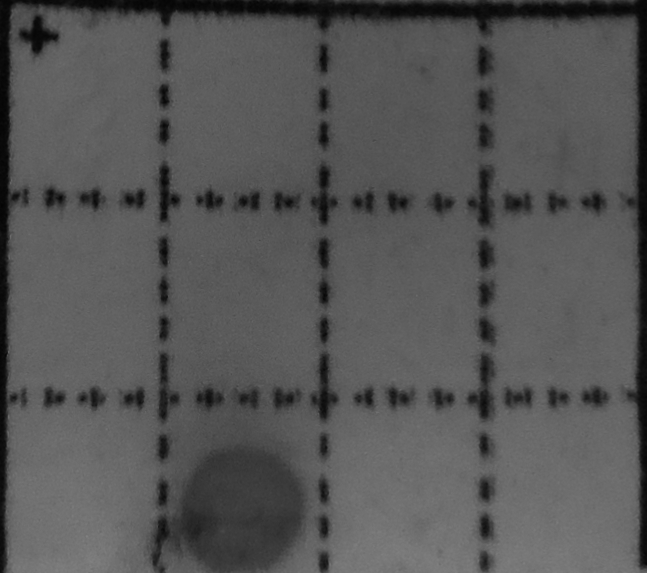

Supplement: Supplemental Information 2 [file peerj-11-15325-s002.zip › Raw Data-2/Results of 179 clinical samples of septicemia by membrane microarray (grayscale)-2/509536.tif]

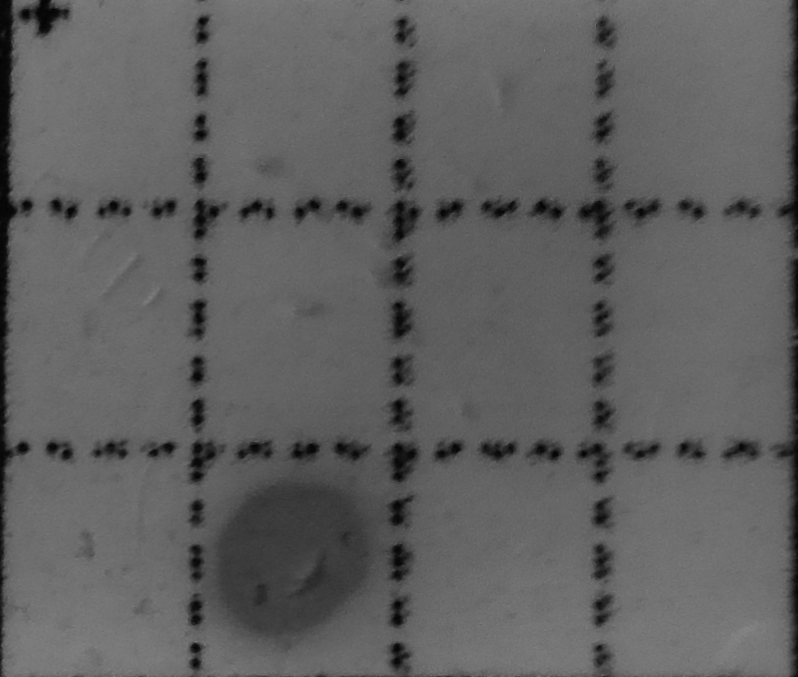

Supplement: Supplemental Information 2 [file peerj-11-15325-s002.zip › Raw Data-2/Results of 179 clinical samples of septicemia by membrane microarray (grayscale)-2/516080.tif]

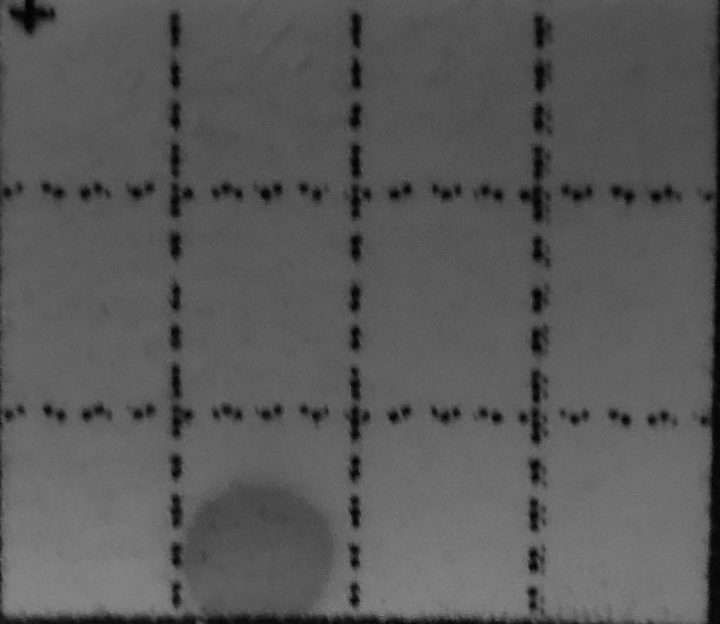

Supplement: Supplemental Information 2 [file peerj-11-15325-s002.zip › Raw Data-2/Results of 179 clinical samples of septicemia by membrane microarray (grayscale)-2/517652.tif]

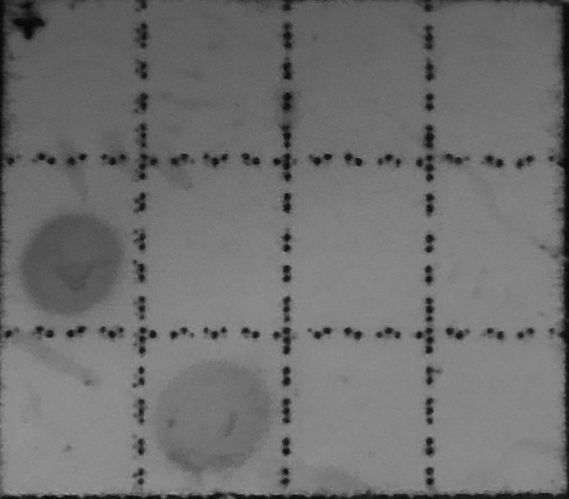

Supplement: Supplemental Information 2 [file peerj-11-15325-s002.zip › Raw Data-2/Results of 179 clinical samples of septicemia by membrane microarray (grayscale)-2/519285.tif]

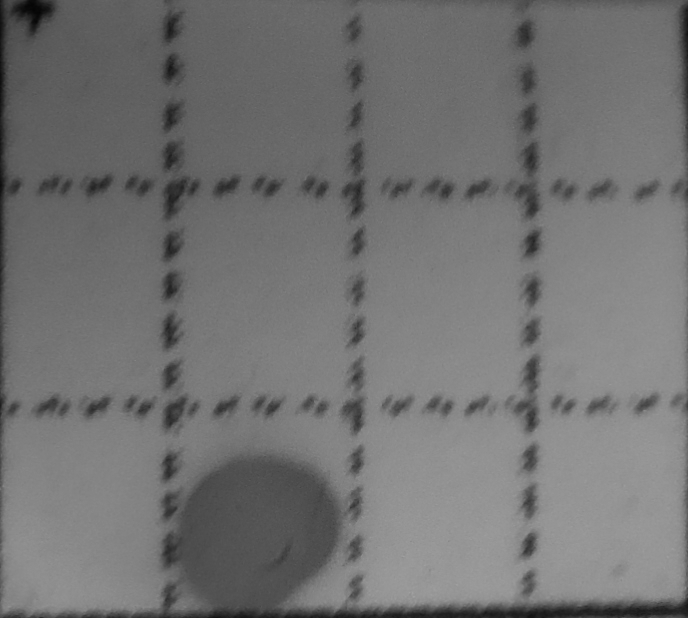

Supplement: Supplemental Information 2 [file peerj-11-15325-s002.zip › Raw Data-2/Results of 179 clinical samples of septicemia by membrane microarray (grayscale)-2/521356.tif]

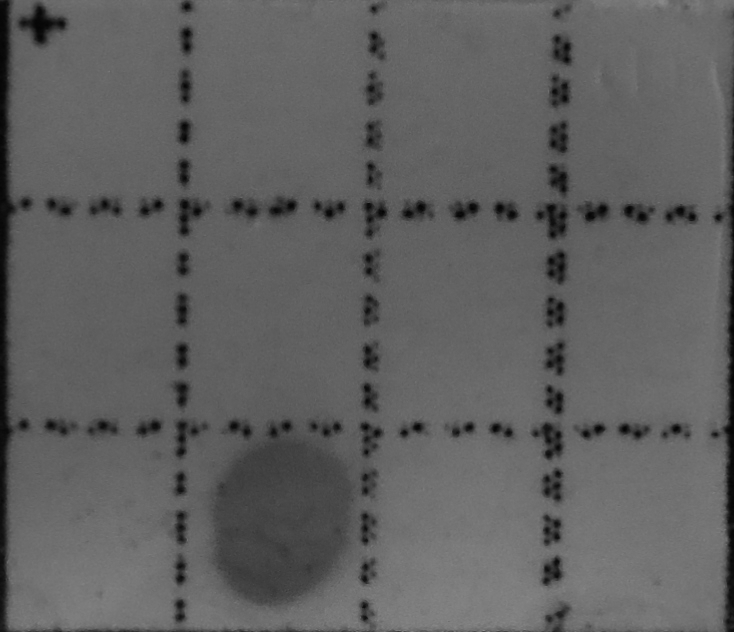

Supplement: Supplemental Information 2 [file peerj-11-15325-s002.zip › Raw Data-2/Results of 179 clinical samples of septicemia by membrane microarray (grayscale)-2/522880.tif]

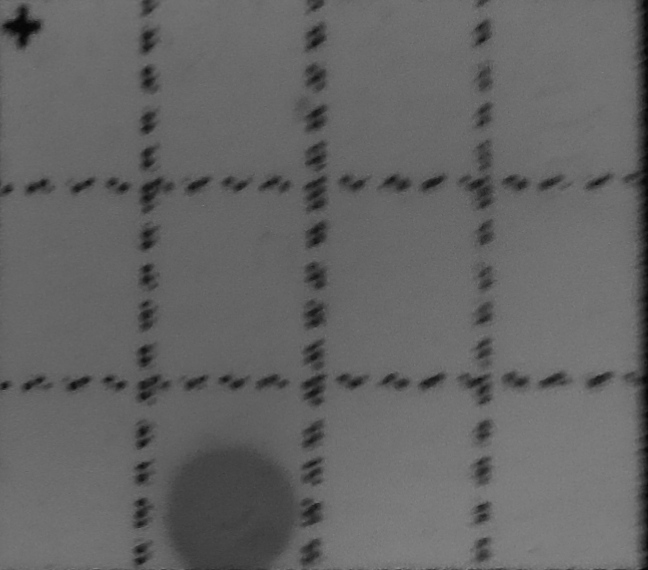

Supplement: Supplemental Information 2 [file peerj-11-15325-s002.zip › Raw Data-2/Results of 179 clinical samples of septicemia by membrane microarray (grayscale)-2/525970.tif]

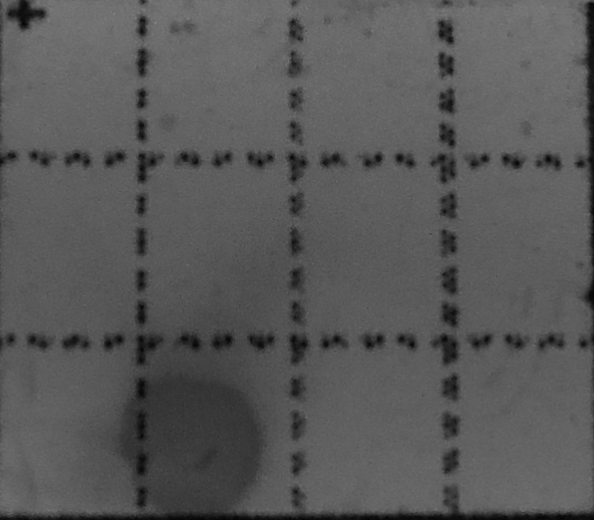

Supplement: Supplemental Information 2 [file peerj-11-15325-s002.zip › Raw Data-2/Results of 179 clinical samples of septicemia by membrane microarray (grayscale)-2/529415.tif]

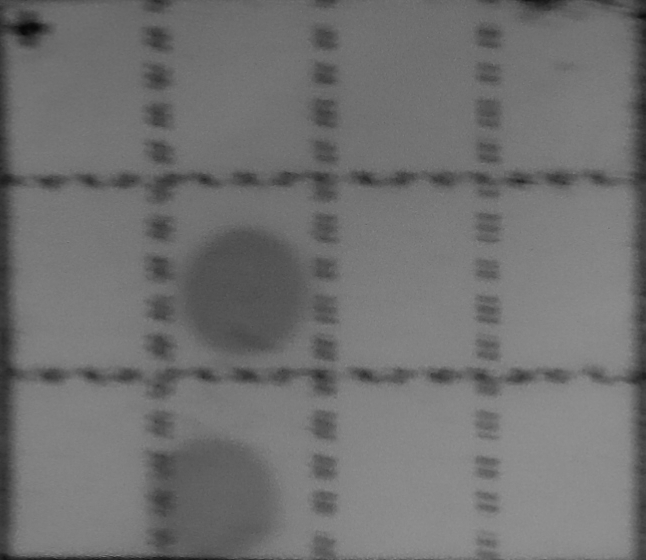

Supplement: Supplemental Information 2 [file peerj-11-15325-s002.zip › Raw Data-2/Results of 179 clinical samples of septicemia by membrane microarray (grayscale)-2/529971.tif]

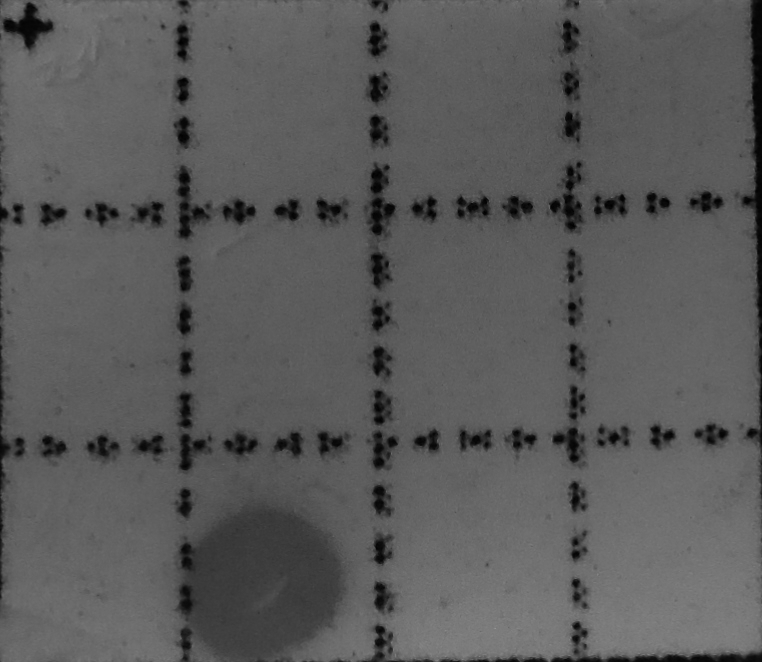

Supplement: Supplemental Information 2 [file peerj-11-15325-s002.zip › Raw Data-2/Results of 179 clinical samples of septicemia by membrane microarray (grayscale)-2/532929.tif]

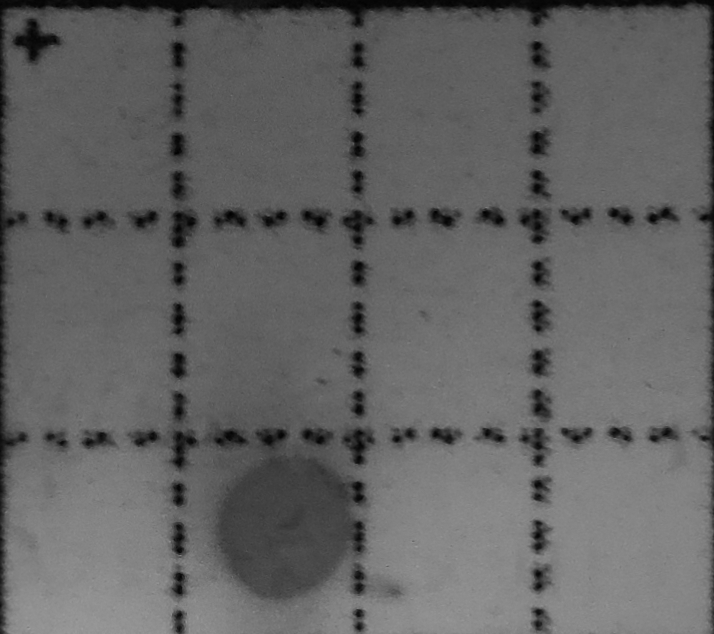

Supplement: Supplemental Information 2 [file peerj-11-15325-s002.zip › Raw Data-2/Results of 179 clinical samples of septicemia by membrane microarray (grayscale)-2/533737.tif]

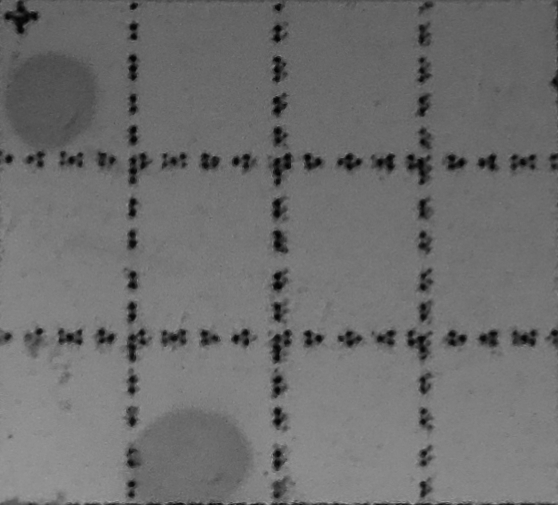

Supplement: Supplemental Information 2 [file peerj-11-15325-s002.zip › Raw Data-2/Results of 179 clinical samples of septicemia by membrane microarray (grayscale)-2/534747.tif]

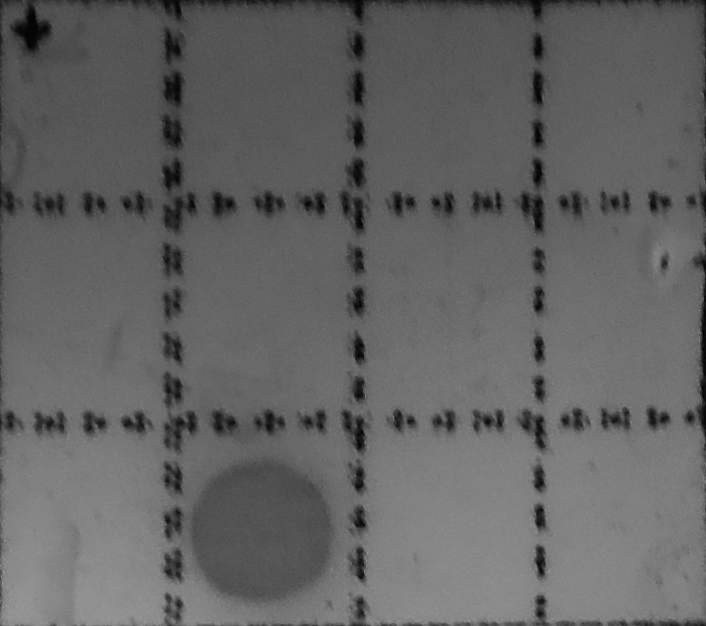

Supplement: Supplemental Information 2 [file peerj-11-15325-s002.zip › Raw Data-2/Results of 179 clinical samples of septicemia by membrane microarray (grayscale)-2/535367.tif]

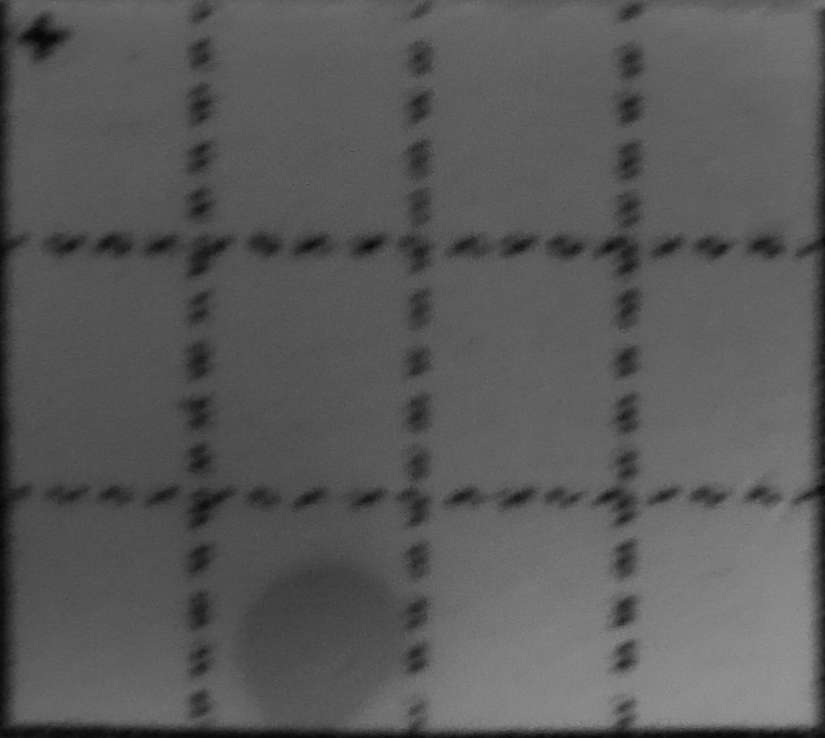

Supplement: Supplemental Information 2 [file peerj-11-15325-s002.zip › Raw Data-2/Results of 179 clinical samples of septicemia by membrane microarray (grayscale)-2/535504.tif]

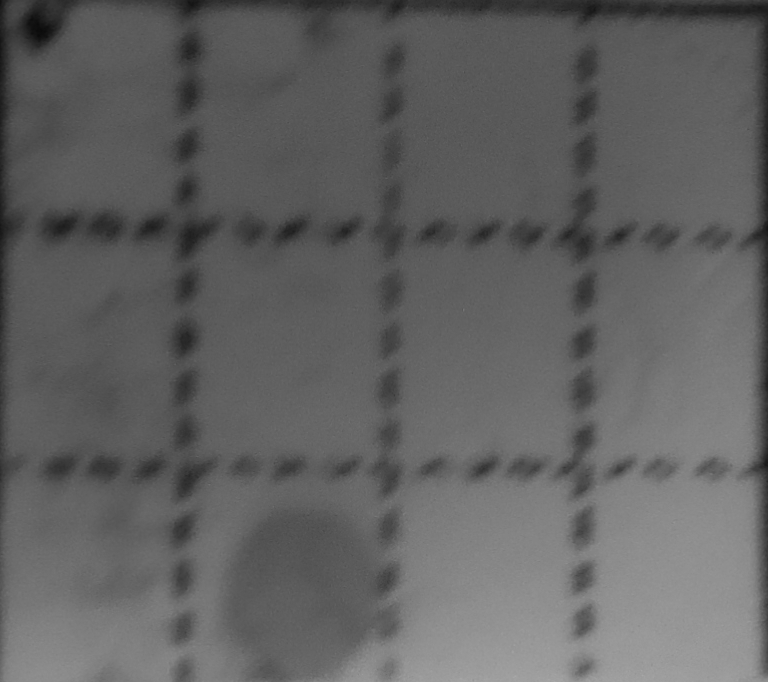

Supplement: Supplemental Information 2 [file peerj-11-15325-s002.zip › Raw Data-2/Results of 179 clinical samples of septicemia by membrane microarray (grayscale)-2/539499.tif]

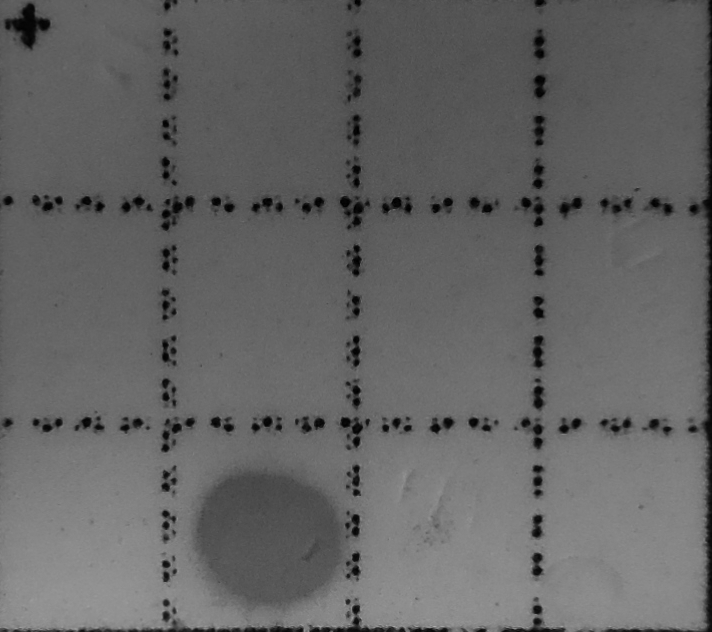

Supplement: Supplemental Information 2 [file peerj-11-15325-s002.zip › Raw Data-2/Results of 179 clinical samples of septicemia by membrane microarray (grayscale)-2/540494.tif]

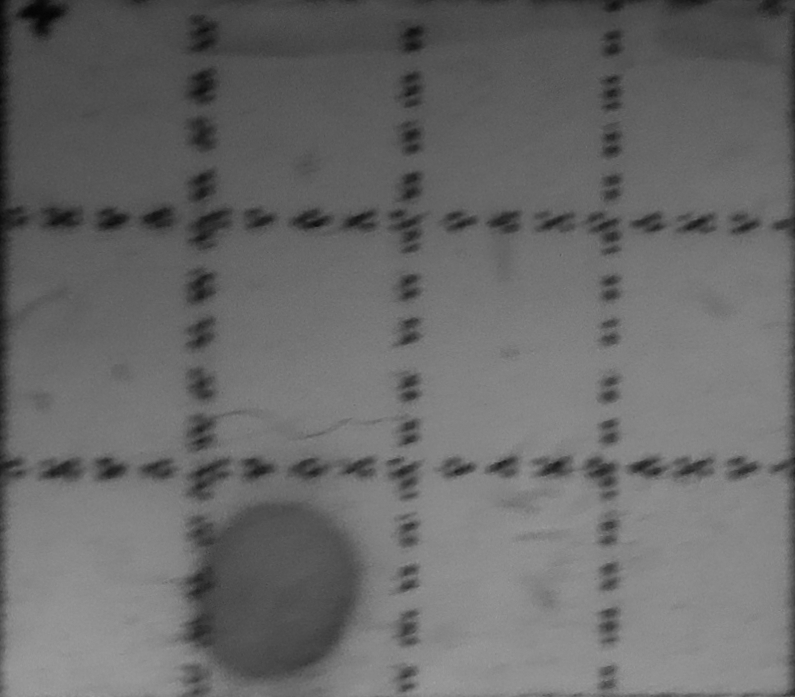

Supplement: Supplemental Information 2 [file peerj-11-15325-s002.zip › Raw Data-2/Results of 179 clinical samples of septicemia by membrane microarray (grayscale)-2/543000.tif]

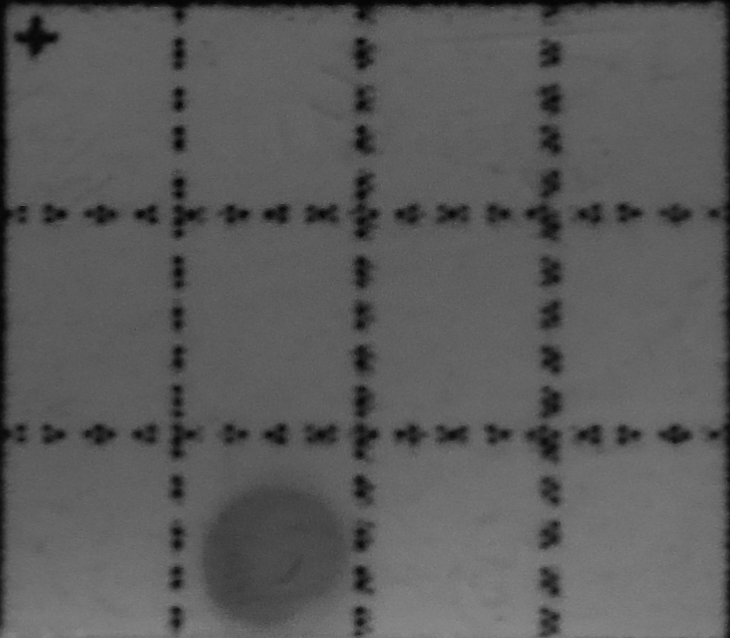

Supplement: Supplemental Information 2 [file peerj-11-15325-s002.zip › Raw Data-2/Results of 179 clinical samples of septicemia by membrane microarray (grayscale)-2/545021.tif]

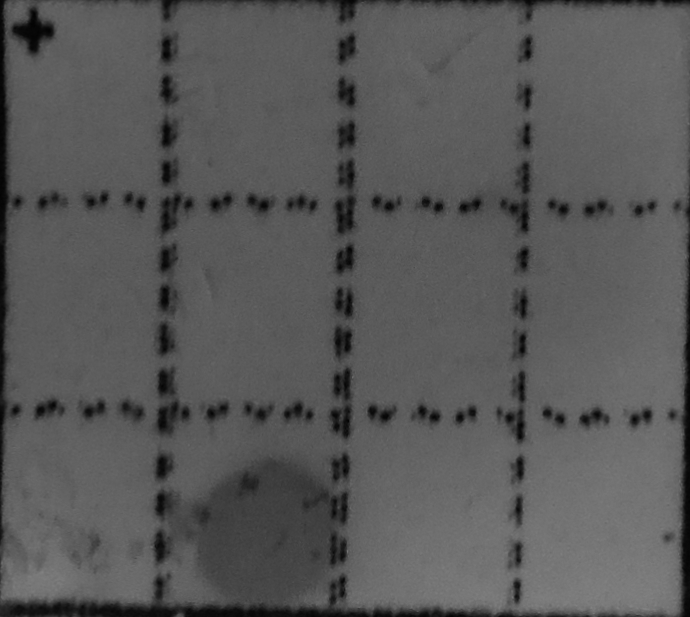

Supplement: Supplemental Information 2 [file peerj-11-15325-s002.zip › Raw Data-2/Results of 179 clinical samples of septicemia by membrane microarray (grayscale)-2/545242.tif]

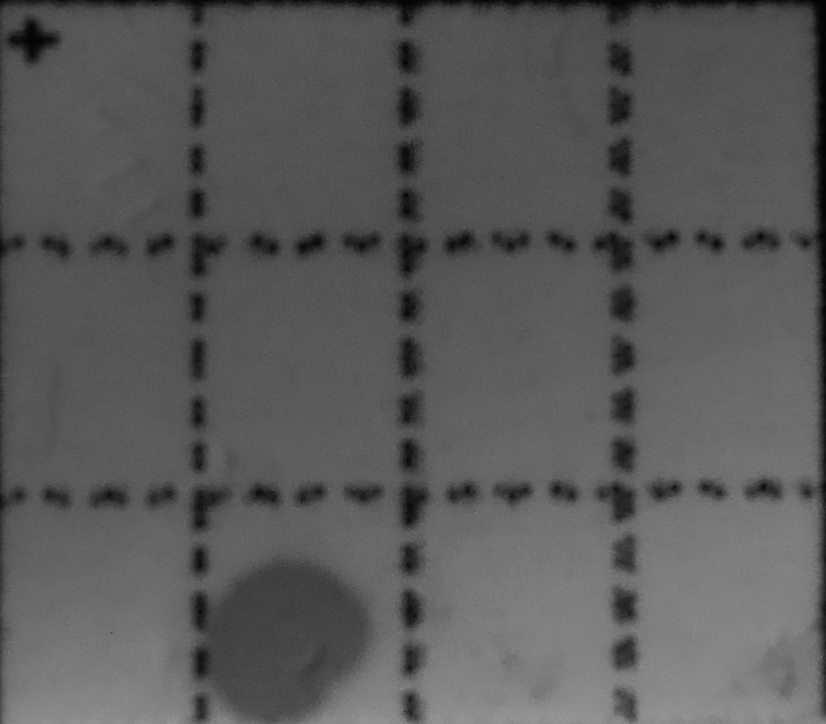

Supplement: Supplemental Information 2 [file peerj-11-15325-s002.zip › Raw Data-2/Results of 179 clinical samples of septicemia by membrane microarray (grayscale)-2/545936.tif]

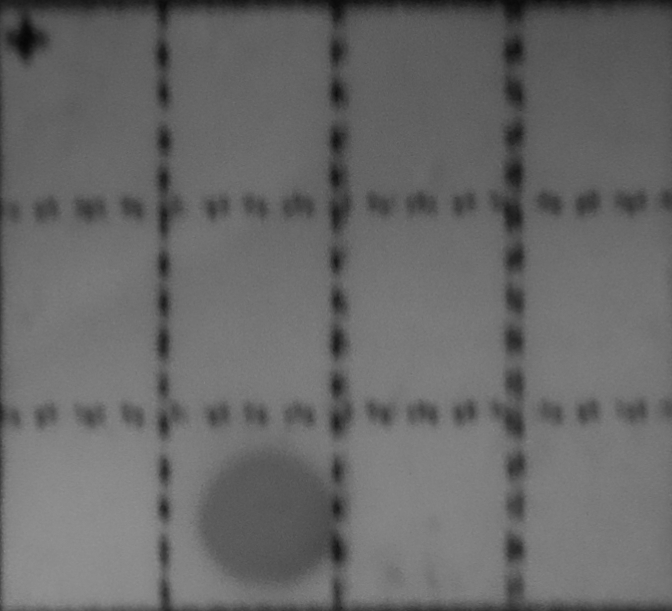

Supplement: Supplemental Information 2 [file peerj-11-15325-s002.zip › Raw Data-2/Results of 179 clinical samples of septicemia by membrane microarray (grayscale)-2/549769.tif]

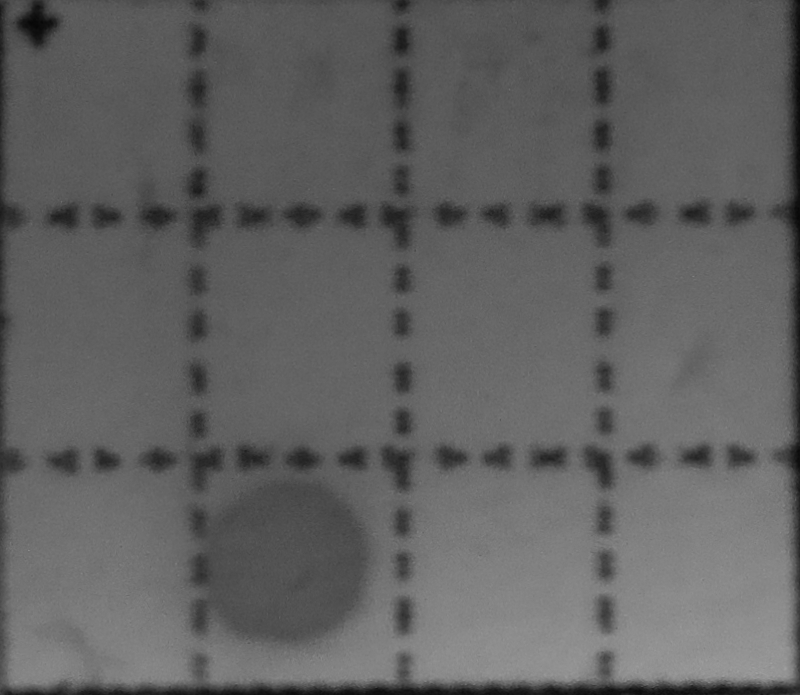

Supplement: Supplemental Information 2 [file peerj-11-15325-s002.zip › Raw Data-2/Results of 179 clinical samples of septicemia by membrane microarray (grayscale)-2/554162.tif]

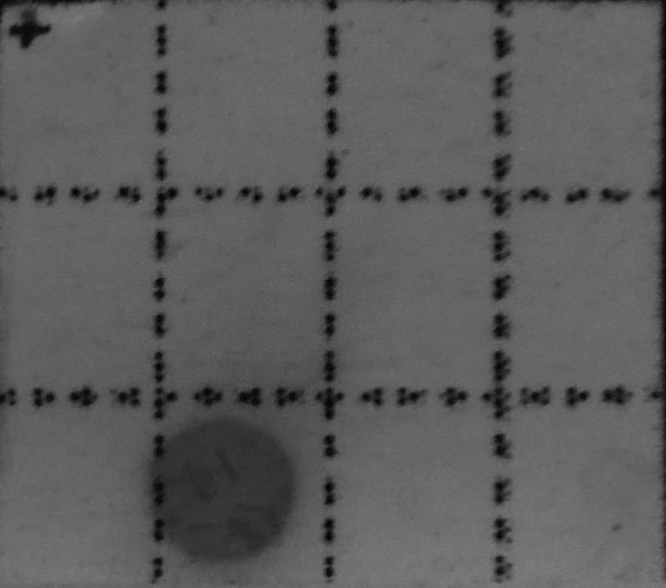

Supplement: Supplemental Information 2 [file peerj-11-15325-s002.zip › Raw Data-2/Results of 179 clinical samples of septicemia by membrane microarray (grayscale)-2/554871.tif]

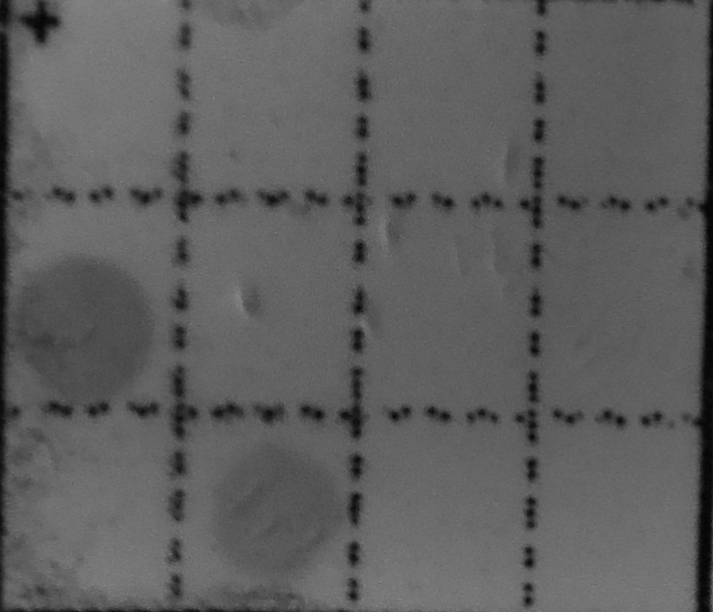

Supplement: Supplemental Information 2 [file peerj-11-15325-s002.zip › Raw Data-2/Results of 179 clinical samples of septicemia by membrane microarray (grayscale)-2/555406.tif]

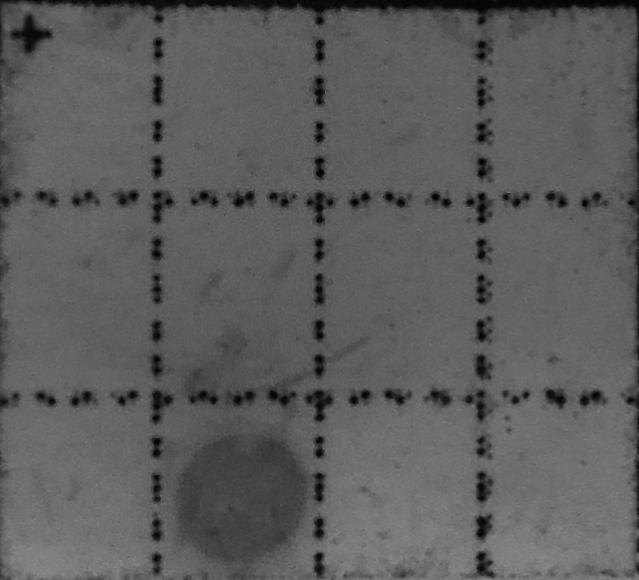

Supplement: Supplemental Information 2 [file peerj-11-15325-s002.zip › Raw Data-2/Results of 179 clinical samples of septicemia by membrane microarray (grayscale)-2/555473.tif]

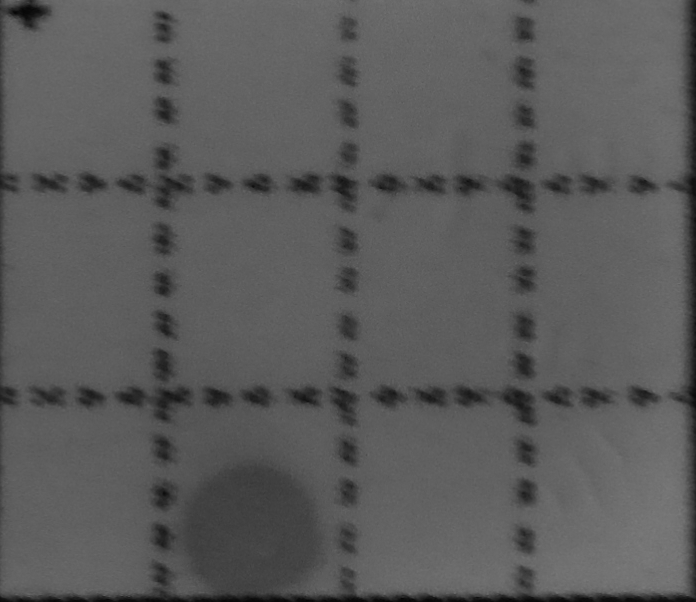

Supplement: Supplemental Information 2 [file peerj-11-15325-s002.zip › Raw Data-2/Results of 179 clinical samples of septicemia by membrane microarray (grayscale)-2/556530.tif]

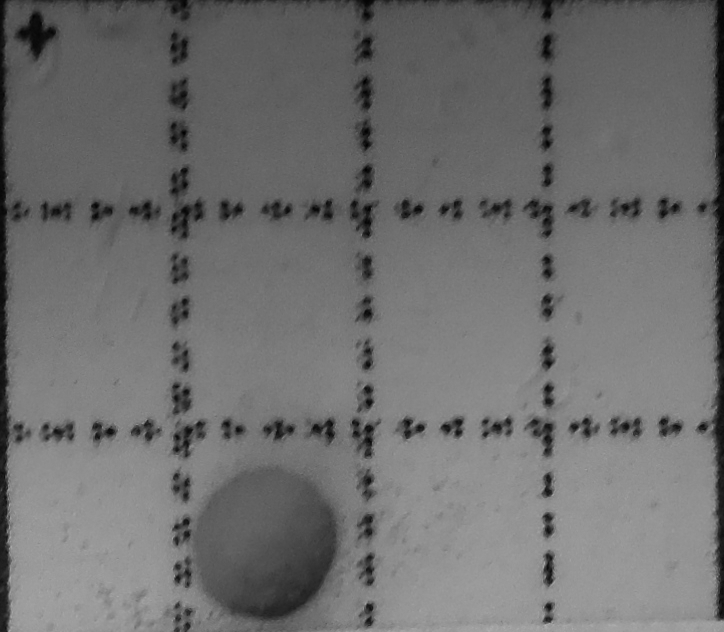

Supplement: Supplemental Information 2 [file peerj-11-15325-s002.zip › Raw Data-2/Results of 179 clinical samples of septicemia by membrane microarray (grayscale)-2/557644.tif]

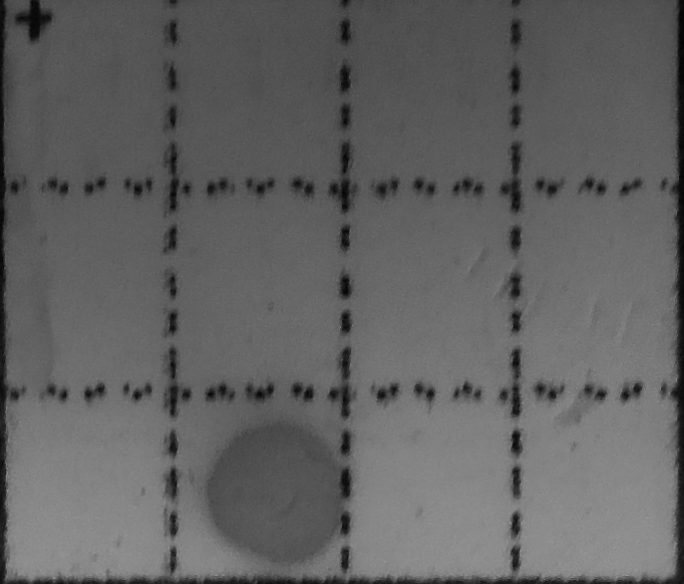

Supplement: Supplemental Information 2 [file peerj-11-15325-s002.zip › Raw Data-2/Results of 179 clinical samples of septicemia by membrane microarray (grayscale)-2/558094.tif]

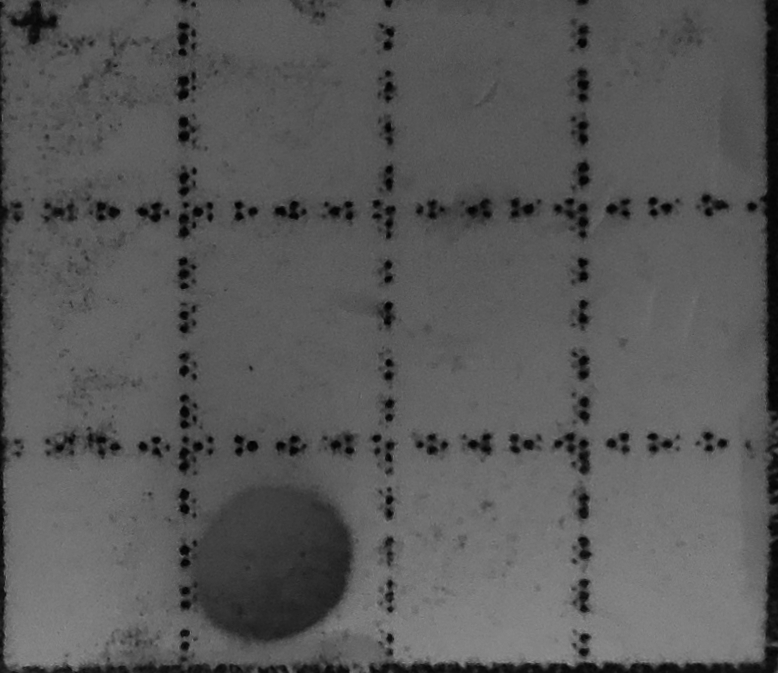

Supplement: Supplemental Information 2 [file peerj-11-15325-s002.zip › Raw Data-2/Results of 179 clinical samples of septicemia by membrane microarray (grayscale)-2/558598.tif]

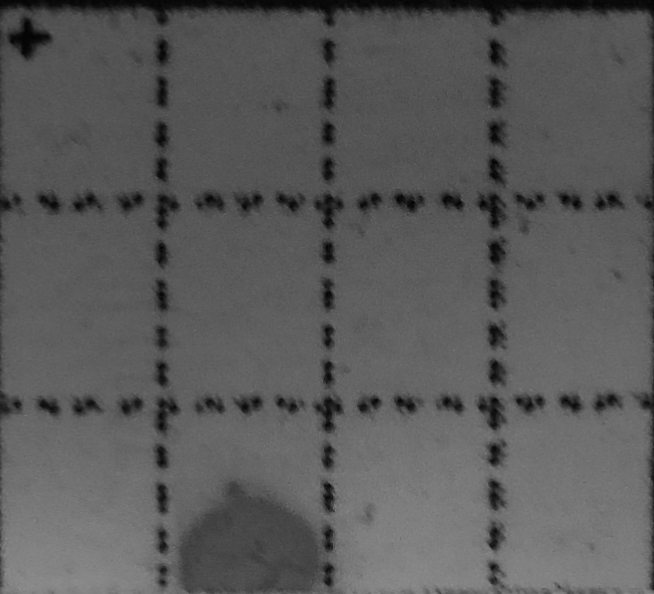

Supplement: Supplemental Information 2 [file peerj-11-15325-s002.zip › Raw Data-2/Results of 179 clinical samples of septicemia by membrane microarray (grayscale)-2/559099.tif]

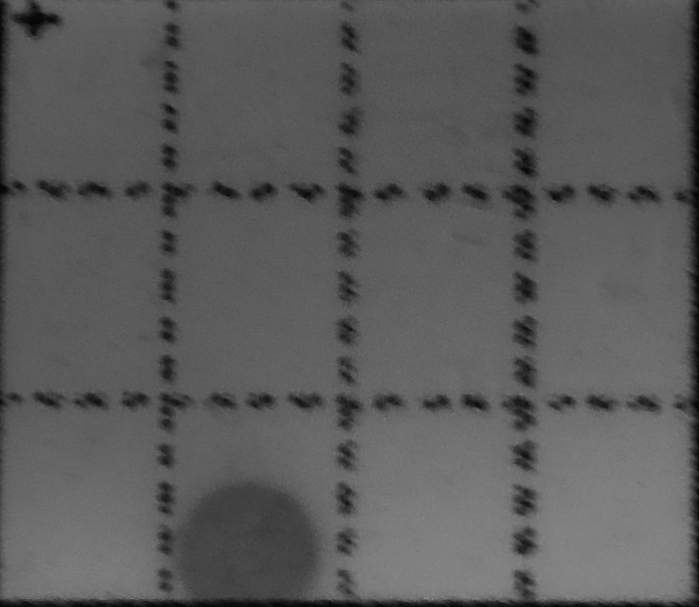

Supplement: Supplemental Information 2 [file peerj-11-15325-s002.zip › Raw Data-2/Results of 179 clinical samples of septicemia by membrane microarray (grayscale)-2/559638.tif]

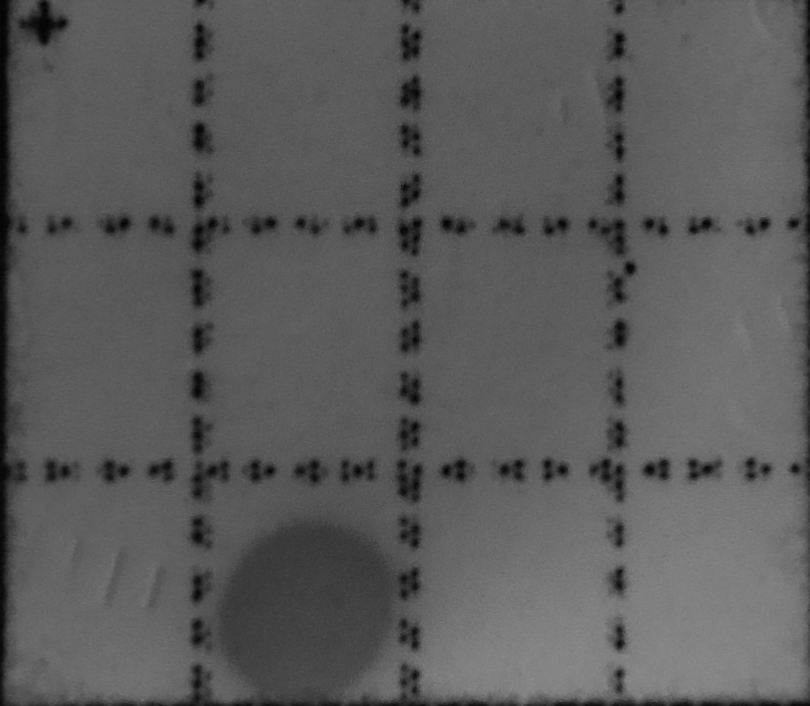

Supplement: Supplemental Information 2 [file peerj-11-15325-s002.zip › Raw Data-2/Results of 179 clinical samples of septicemia by membrane microarray (grayscale)-2/559722.tif]

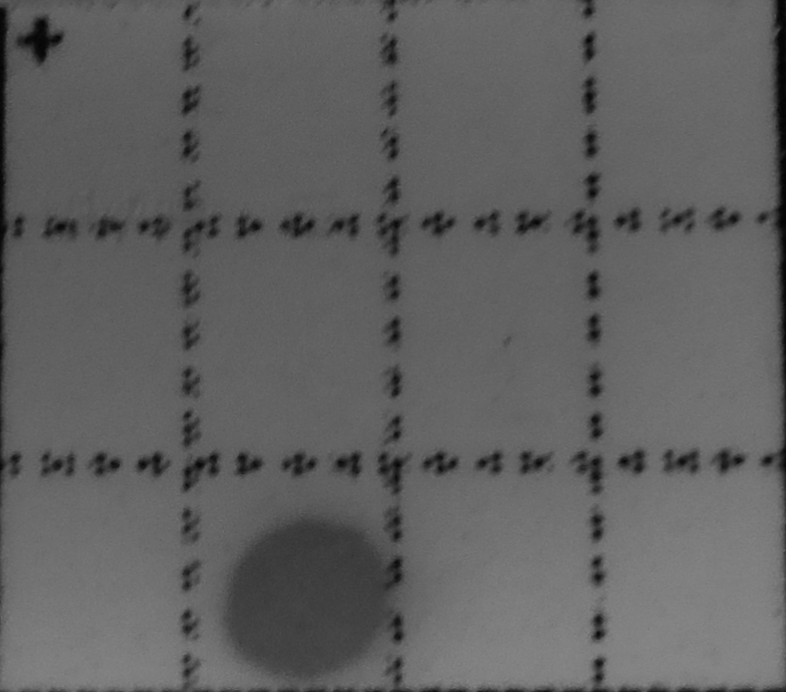

Supplement: Supplemental Information 2 [file peerj-11-15325-s002.zip › Raw Data-2/Results of 179 clinical samples of septicemia by membrane microarray (grayscale)-2/58274.tif]

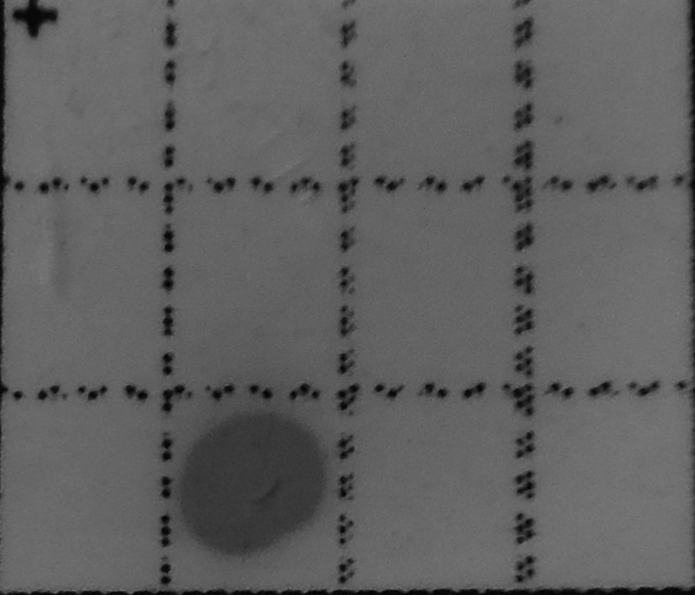

Supplement: Supplemental Information 2 [file peerj-11-15325-s002.zip › Raw Data-2/Results of 179 clinical samples of septicemia by membrane microarray (grayscale)-2/6833.tif]

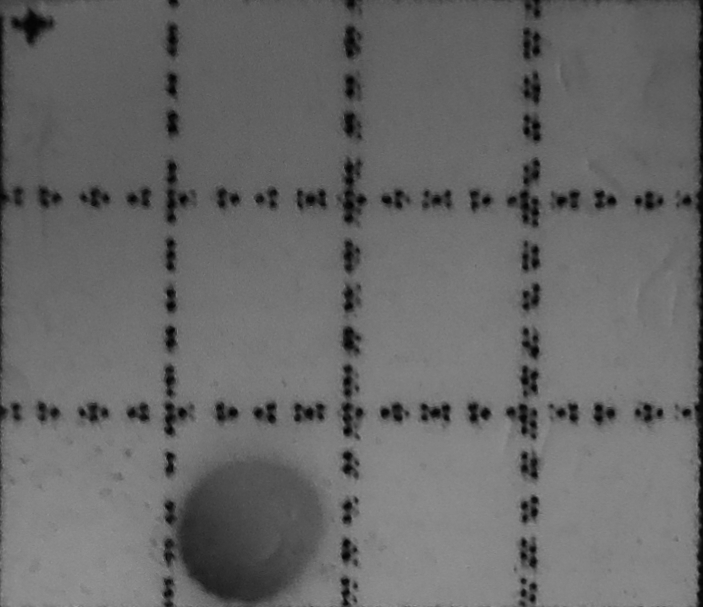

Supplement: Supplemental Information 2 [file peerj-11-15325-s002.zip › Raw Data-2/Results of 179 clinical samples of septicemia by membrane microarray (grayscale)-2/75004.tif]
