# Supplementary material for: A new application of multiplex PCR combined with membrane biochip assay for rapid detection of 9 common pathogens in sepsis
Source: PeerJ. 2023 May 12;11:e15325. doi: 10.7717/peerj.15325 (PMC10184654; doi:10.7717/peerj.15325)
Supplement: Supplemental Information 3 [file peerj-11-15325-s003.docx]

**Table S1:** The detective results of membrane chip assay and blood culture.

| **Number** | **Gender** | **Age** | **Results of**  **membrane chip assay** | **Results of**  **multiplex PCR†** | **Results of**  **blood culture** |
| --- | --- | --- | --- | --- | --- |
| WF-001 | male | 70 | negative | No | negative |
| WF-002 | female | 24 | negative | No | negative |
| WF-003 | male | 46 | *Klebsiella pneumoniae* | Yes | *Klebsiella pneumoniae* |
| WF-004 | female | 25 | negative | No | negative |
| WF-005 | male | 47 | negative | No | negative |
| WF-006 | female | 53 | negative | No | negative |
| WF-007 | male | 37 | *Streptococcus pneumoniae* | Yes | *Streptococcus pneumoniae* |
| WF-008 | male | 66 | negative | No | negative |
| WF-009 | male | 32 | negative | No | negative |
| WF-010 | female | 44 | negative | No | negative |
| WF-011 | male | 46 | negative | No | negative |
| WF-012 | male | 43 | negative | No | negative |
| WF-013 | female | 32 | *Escherichia coli* | Yes | *Escherichia coli* |
| WF-014 | female | 55 | negative | No | negative |
| WF-015 | female | 26 | negative | No | negative |
| WF-016 | female | 52 | negative | No | negative |
| WF-017 | male | 21 | negative | No | negative |
| WF-018 | female | 58 | negative | No | negative |
| WF-019 | male | 55 | negative | No | *Klebsiella ornithinolytica* |
| WF-020 | male | 45 | negative | No | negative |
| WF-021 | male | 29 | negative | No | negative |
| WF-022 | male | 25 | negative | No | negative |
| WF-023 | male | 82 | negative | No | negative |
| WF-024 | male | 63 | negative | No | negative |
| WF-025 | male | 17 | negative | No | *Enterococcus faecium* |
| WF-026 | male | 35 | negative | No | negative |
| WF-027 | female | 55 | negative | No | negative |
| WF-028 | male | 52 | negative | No | negative |
| WF-029 | male | 59 | negative | No | negative |
| WF-030 | male | 75 | negative | No | negative |
| WF-031 | male | 77 | *Staphylococcus epidermidis* | Yes | *Staphylococcus epidermidis* |
| WF-032 | female | 62 | negative | No | negative |
| WF-033 | female | 52 | negative | No | negative |
| WF-034 | male | 74 | negative | No | negative |
| WF-035 | female | 92 | *Escherichia coli* | Yes | *Escherichia coli* |
| WF-036 | male | 62 | negative | No | negative |
| WF-037 | male | 46 | *Escherichia coli* | Yes | *Escherichia coli* |
| WF-038 | female | 27 | negative | No | negative |
| WF-039 | male | 16 | negative | No | negative |
| WF-040 | male | 66 | negative | No | negative |
| WF-041 | female | 15 | negative | No | negative |
| WF-042 | male | 21 | negative | No | negative |
| WF-043 | male | 47 | negative | No | negative |
| WF-044 | female | 21 | negative | No | negative |
| WF-045 | male | 62 | negative | No | negative |
| WF-046 | male | 53 | *Escherichia coli* | Yes | *Escherichia coli* |
| WF-047 | male | 59 | negative | No | negative |
| WF-048 | female | 61 | negative | No | negative |
| WF-049 | male | 87 | negative | No | negative |
| WF-050 | male | 21 | negative | No | negative |
| WF-051 | male | 28 | negative | No | negative |
| WF-052 | female | 55 | negative | No | negative |
| WF-053 | female | 33 | *Klebsiella pneumoniae* | Yes | *Klebsiella pneumoniae* |
| WF-054 | male | 27 | negative | No | negative |
| WF-055 | male | 84 | negative | No | negative |
| WF-056 | female | 55 | *Escherichia coli* | Yes | negative |
| WF-057 | male | 49 | negative | No | negative |
| WF-058 | male | 15 | negative | No | negative |
| WF-059 | female | 58 | negative | No | negative |
| WF-060 | male | 36 | negative | No | negative |
| WF-061 | male | 57 | negative | No | *Enterobacter aerogenes* |
| WF-062 | female | 36 | negative | No | negative |
| WF-063 | male | 47 | *Acinetobacter baumannii* | Yes | *Acinetobacter baumannii* |
| WF-064 | female | 63 | negative | No | negative |
| WF-065 | female | 44 | negative | No | *Stenotrophomonas maltophilia* |
| WF-066 | male | 51 | negative | No | negative |
| WF-067 | male | 60 | negative | No | negative |
| WF-068 | female | 29 | negative | No | negative |
| WF-069 | male | 59 | negative | No | negative |
| WF-070 | female | 40 | negative | No | negative |
| WF-071 | male | 14 | *Enterococcus faecalis* | Yes | *Enterococcus faecalis* |
| WF-072 | male | 57 | negative | No | negative |
| WF-073 | male | 44 | negative | No | *Escherichia coli* |
| WF-074 | female | 14 | negative | No | negative |
| WF-075 | male | 31 | *Pseudomonas aeruginosa* | Yes | *Pseudomonas aeruginosa* |
| WF-076 | female | 63 | *Klebsiella pneumoniae* | Yes | negative |
| WF-077 | male | 42 | negative | No | negative |
| WF-078 | female | 45 | negative | No | negative |
| WF-079 | male | 38 | negative | No | negative |
| WF-080 | female | 26 | negative | No | negative |
| WF-081 | female | 36 | negative | No | negative |
| WF-082 | male | 34 | *Staphylococcus epidermidis* | Yes | *Staphylococcus epidermidis* |
| WF-083 | male | 38 | negative | No | negative |
| WF-084 | female | 55 | negative | No | negative |
| WF-085 | female | 81 | negative | No | negative |
| WF-086 | male | 44 | negative | No | negative |
| WF-087 | female | 51 | *Staphylococcus aureus* | Yes | *Staphylococcus aureus* |
| WF-088 | female | 54 | negative | No | negative |
| WF-089 | male | 55 | negative | No | negative |
| WF-090 | female | 51 | negative | No | negative |
| WF-091 | male | 44 | *Candida albicans* | Yes | *Candida albicans* |
| WF-092 | female | 78 | negative | No | negative |
| WF-093 | female | 30 | negative | No | negative |
| WF-094 | male | 15 | negative | No | negative |
| WF-095 | male | 32 | negative | No | negative |
| WF-096 | male | 34 | negative | No | negative |
| WF-097 | male | 19 | negative | No | negative |
| WF-098 | male | 55 | *Staphylococcus epidermidis* | Yes | *Staphylococcus epidermidis* |
| WF-099 | female | 30 | negative | No | negative |
| WF-100 | male | 41 | negative | No | negative |
| WF-101 | female | 47 | *Staphylococcus aureus* | Yes | *Staphylococcus aureus* |
| WF-102 | male | 63 | *Pseudomonas aeruginosa* | Yes | *Pseudomonas aeruginosa* |
| WF-103 | male | 25 | negative | No | negative |
| WF-104 | male | 71 | negative | No | negative |
| WF-105 | male | 37 | negative | No | negative |
| WF-106 | male | 58 | negative | No | negative |
| WF-107 | female | 33 | negative | No | negative |
| WF-108 | male | 61 | negative | No | negative |
| WF-109 | female | 61 | negative | No | negative |
| WF-110 | female | 31 | *Acinetobacter baumannii* | Yes | *Acinetobacter baumannii* |
| WF-111 | male | 70 | negative | No | negative |
| WF-112 | female | 60 | negative | No | negative |
| WF-113 | female | 55 | *Enterococcus faecalis* | Yes | *Enterococcus faecalis* |
| WF-114 | male | 36 | negative | No | negative |
| WF-115 | female | 31 | negative | No | negative |
| WF-116 | male | 71 | negative | No | *Escherichia hermannii* |
| WF-117 | female | 22 | negative | No | negative |
| WF-118 | male | 27 | negative | No | negative |
| WF-119 | male | 28 | negative | No | negative |
| WF-120 | male | 75 | negative | No | *Klebsiella pneumoniae* |
| WF-121 | female | 67 | negative | No | negative |
| WF-122 | female | 54 | negative | No | negative |
| WF-123 | male | 65 | *Klebsiella pneumoniae* | Yes | *Klebsiella pneumoniae* |
| WF-124 | female | 43 | negative | No | negative |
| WF-125 | female | 55 | negative | No | negative |
| WF-126 | male | 69 | negative | No | negative |
| WF-127 | female | 47 | negative | No | negative |
| WF-128 | male | 70 | negative | No | negative |
| WF-129 | female | 52 | *Acinetobacter baumannii* | Yes | *Acinetobacter baumannii* |
| WF-130 | male | 67 | negative | No | negative |
| WF-131 | male | 26 | negative | No | negative |
| WF-132 | female | 28 | negative | No | negative |
| WF-133 | male | 60 | negative | No | negative |
| WF-134 | female | 71 | negative | No | negative |
| WF-135 | male | 54 | *Pseudomonas aeruginosa* | Yes | *Pseudomonas aeruginosa* |
| WF-136 | female | 64 | negative | No | negative |
| WF-137 | female | 37 | negative | No | negative |
| WF-138 | female | 57 | negative | No | negative |
| WF-139 | male | 48 | *Staphylococcus aureus* | Yes | *Staphylococcus aureus* |
| WF-140 | male | 54 | negative | No | negative |
| WF-141 | male | 69 | negative | No | negative |
| WF-142 | male | 60 | negative | No | negative |
| WF-143 | male | 50 | negative | No | negative |
| WF-144 | female | 58 | negative | No | negative |
| WF-145 | male | 44 | negative | No | negative |
| WF-146 | female | 35 | *Escherichia coli* | Yes | *Escherichia coli* |
| WF-147 | female | 61 | negative | No | negative |
| WF-148 | male | 62 | negative | No | negative |
| WF-149 | female | 55 | negative | No | negative |
| WF-150 | male | 54 | negative | No | negative |
| WF-151 | male | 54 | negative | No | negative |
| WF-152 | female | 29 | negative | No | negative |
| WF-153 | female | 54 | negative | No | negative |
| WF-154 | male | 64 | negative | No | negative |
| WF-155 | male | 50 | *Klebsiella pneumoniae* | Yes | *Klebsiella pneumoniae* |
| WF-156 | male | 54 | negative | No | negative |
| WF-157 | male | 67 | negative | No | negative |
| WF-158 | male | 52 | negative | No | negative |
| WF-159 | female | 46 | negative | No | negative |
| WF-160 | male | 41 | negative | No | negative |
| WF-161 | male | 75 | negative | No | negative |
| WF-162 | male | 64 | *Acinetobacter baumannii* | Yes | *Acinetobacter baumannii* |
| WF-163 | female | 24 | negative | No | negative |
| WF-164 | female | 65 | negative | No | negative |
| WF-165 | male | 55 | negative | No | negative |
| WF-166 | male | 68 | negative | No | negative |
| WF-167 | male | 55 | negative | No | negative |
| WF-168 | female | 61 | negative | No | negative |
| WF-169 | male | 52 | *Streptococcus pneumoniae* | Yes | negative |
| WF-170 | male | 67 | *Escherichia coli* | Yes | negative |
| WF-171 | female | 49 | negative | No | negative |
| WF-172 | female | 71 | *Acinetobacter baumannii* | Yes | negative |
| WF-173 | male | 28 | *Staphylococcus aureus* | Yes | negative |
| WF-174 | female | 65 | *Enterococcus faecalis* | Yes | negative |
| WF-175 | female | 22 | negative | No | negative |
| WF-176 | male | 65 | negative | No | negative |
| WF-177 | male | 55 | *Staphylococcus epidermidis* | Yes | negative |
| WF-178 | male | 60 | *Klebsiella pneumoniae* | Yes | negative |
| WF-179 | male | 50 | *Escherichia coli* | Yes | negative |

†, The "No" indicates that no electrophoretic bands were produced using multiplex PCR, while "Yes" indicates that electrophoretic bands were produced.

**Table S2:** Comparison of clinical sensitivity and specificity in different articles.

|  | **In this paper (%)** | **In previous paper (%) †** | ***P*-value** |
| --- | --- | --- | --- |
| **Sensitivity** | | | |
| Positive result | 26 (92.9%) | 63 (95.5%) | 0.608 |
| Negative result | 2 (7.1%) | 3 (4.5%) |  |
| **Specificity** | | | |
| Positive result | 10 (6.8%) | 0 (0%) | 0.295 |
| Negative result | 136 (93.2%) | 15 (100%) |  |

P-value, Person test; *, P-value < 0.05; **†**, Wang *et al*. 2016.
